# Convergence, divergence, and macroevolutionary constraint as revealed by anatomical network analysis of the squamate skull, with an emphasis on snakes

**DOI:** 10.1038/s41598-022-18649-z

**Published:** 2022-08-25

**Authors:** Catherine R. C. Strong, Mark D. Scherz, Michael W. Caldwell

**Affiliations:** 1 Department of Biological Sciences, University of Alberta, Edmonton, Canada; 2 Natural History Museum of Denmark, University of Copenhagen, Universitetsparken 15, 2100 Copenhagen Ø, Denmark; 3 Department of Earth and Atmospheric Sciences, University of Alberta, Edmonton, Canada; 4 Present Address: Museum of Comparative Zoology and Department of Organismic and Evolutionary Biology, Harvard University, 26 Oxford Street, Cambridge, MA 02138 USA

**Keywords:** Herpetology, Evolution, Modularity

## Abstract

Traditionally considered the earliest-diverging group of snakes, scolecophidians are central to major evolutionary paradigms regarding squamate feeding mechanisms and the ecological origins of snakes. However, quantitative analyses of these phenomena remain scarce. Herein, we therefore assess skull modularity in squamates via anatomical network analysis, focusing on the interplay between ‘microstomy’ (small-gaped feeding), fossoriality, and miniaturization in scolecophidians. Our analyses reveal distinctive patterns of jaw connectivity across purported ‘microstomatans’, thus supporting a more complex scenario of jaw evolution than traditionally portrayed. We also find that fossoriality and miniaturization each define a similar region of topospace (i.e., connectivity-based morphospace), with their combined influence imposing further evolutionary constraint on skull architecture. These results ultimately indicate convergence among scolecophidians, refuting widespread perspectives of these snakes as fundamentally plesiomorphic and morphologically homogeneous. This network-based examination of skull modularity—the first of its kind for snakes, and one of the first to analyze squamates—thus provides key insights into macroevolutionary trends among squamates, with particular implications for snake origins and evolution.

## Introduction

Scolecophidians (‘blindsnakes’) have traditionally been considered fundamentally plesiomorphic among snakes, and thus have featured prominently in centuries-long controversies regarding the ecological and phylogenetic origins of this group^1^. The miniaturized and fossorial ecomorphology of scolecophidians is often viewed as reflecting the ancestral snake condition (e.g., Refs.^2–7^), although this perspective is not universal. Over the past several decades, many authors (e.g., Refs.^8–13^) have instead suggested that scolecophidians may be a highly autapomorphic group not strictly reflecting an ancestral snake morphology; notably, though, only recently has this latter hypothesis been examined in detail and strongly advocated (e.g., Refs.^1,14–17^). This dissenting perspective focuses largely on the combined roles of miniaturization, fossoriality, and heterochrony in misleading existing perspectives on snake evolution^1,14–16^. Indeed, fossoriality and miniaturization are widely recognized as major sources of convergence in vertebrates^18–20^, and particularly squamates^9,14,15,19,21–34^, which has contributed greatly to ongoing conflicts in hypotheses of squamate evolution^19,35^.

**Figure 1:**
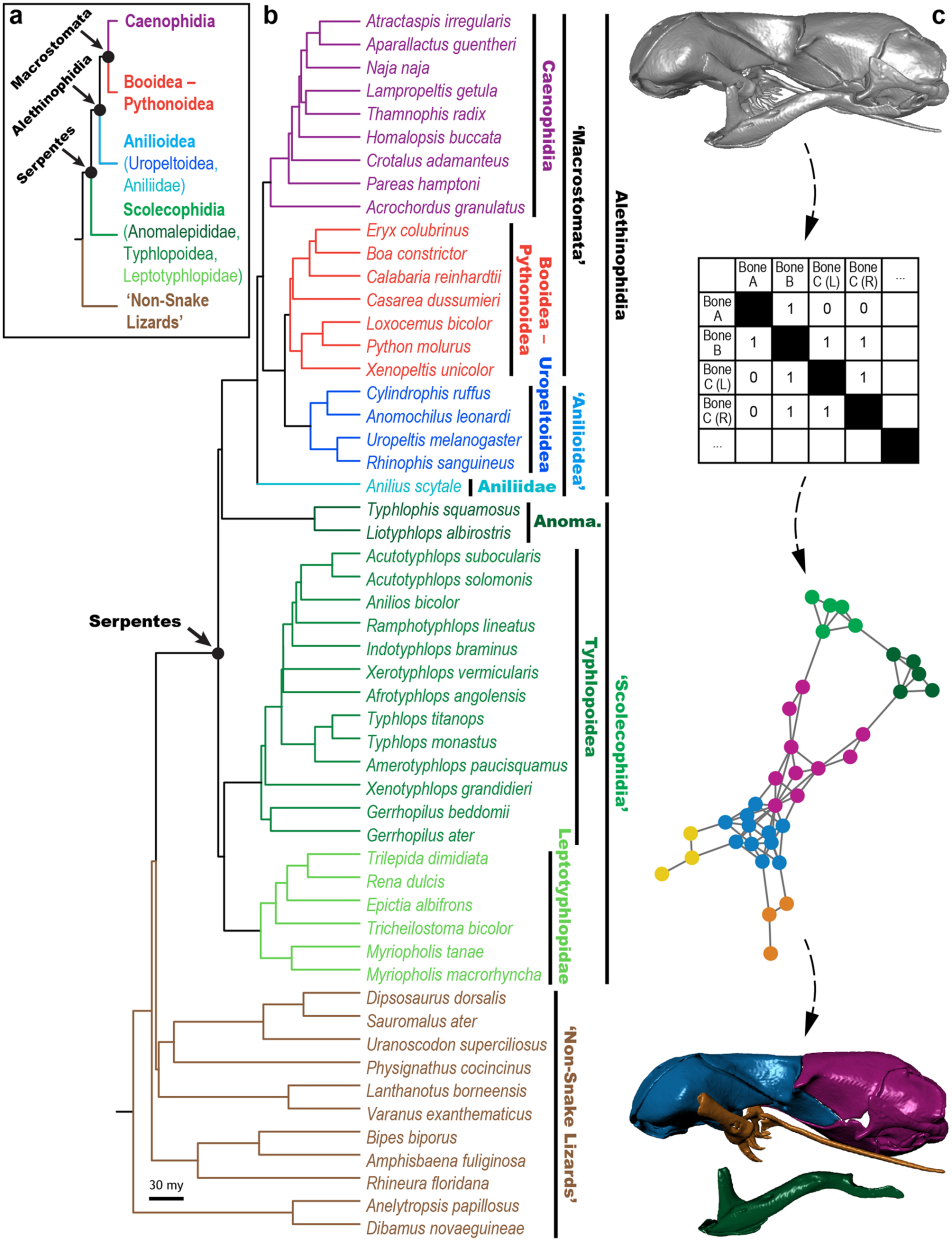
Phylogenetic context and methodological overview. (**a**) Traditional and (**b**) recent phylogenies of Squamata, derived from Rieppel^9^ and Zheng and Wiens^135^, respectively. Major differences include the paraphyly of ‘Scolecophidia’ and polyphyly of ‘Anilioidea’ and ‘Macrostomata’ in molecular phylogenies (**b**), as opposed to their respective monophyly under traditional, morphology-based views of snake evolution (**a**). The phylogeny in (**b**) also provides phylogenetic context for the specimens examined herein (see also Supplementary Table S1); note, however, that our analysis also examined four other anomalepidids—*Anomalepis mexicanus*, *Helminthophis praeocularis*, *Liotyphlops argaleus*, and *L. beui*—that are not included in (**b**) because equivalent taxa were not sampled in the source phylogeny^135^. (**c**) Overview of network modelling during anatomical network analysis (AnNA), showing how this method conceptualizes an anatomical system (in this case, the skull) as a set of interconnected ‘nodes’ (in this case, skull bones). First, the skeletal anatomy is coded into an adjacency matrix, in which scores of ‘1’ *versus* ‘0’ reflect the presence *versus* absence of a connection between two given bones; this allows the skull to be treated purely in terms of the topological relations among its constituent elements. This network is then analyzed via a clustering algorithm and partitioned into modules (see “Methods” section), each of which comprises a group of skull bones that interconnect more thoroughly among each other than they do with other such groups. Colours in (**a**–**b**) indicate corresponding higher taxa, and in (**c**) indicate corresponding modules. *Anoma.* Anomalepididae. Phylogeny in (**b**) and network in (**c**) generated in R [v.4.0.3]^122^ and RStudio [v.1.3.1093]^123^; specimen visualized in Dragonfly [v.4.1]^114^. MCZ scan data used by permission of the Museum of Comparative Zoology, Harvard University.

Prominent among the purportedly plesiomorphic conditions exhibited by scolecophidians—and in turn playing a major role in recent re-examinations of snake evolution and hypotheses of convergence (e.g., Refs.^15,16^)—is the feeding mechanism of ‘microstomy’. Snakes have traditionally been divided into two categories based on jaw mechanics: ‘macrostomy’ and ‘microstomy’ (see Refs.^1,9^ and historical overviews therein). Reflecting the ability of a snake to consume prey items larger than its own head^1,36–38^, ‘macrostomy’ has historically been considered a synapomorphic condition uniting ‘advanced’ snakes (i.e., booid-pythonoids and caenophidians) into the clade Macrostomata (Fig. 1a). In contrast, ‘microstomy’ is the inability to consume these proportionally large prey items^1,9^. Traditionally considered present in early-diverging snakes such as scolecophidians and anilioids, as well as in non-snake lizards (Fig. 1), the ‘microstomatan’ feeding mechanisms of these taxa are typically viewed as homologous, with scolecophidians in particular portrayed as retaining the non-snake lizard condition (e.g., Refs.^6,37^).

However, this traditional morphofunctional categorization has faced recent criticism. ‘Macrostomatan’ snakes have increasingly been recovered as non-monophyletic in molecular (Fig. 1b; e.g., Refs.^7,39–42^) and combined-data (e.g., Ref.^29^) analyses, with booid-pythonoids and caenophidians also undergoing different ontogenetic trajectories before reaching their respective endpoint ‘macrostomatan’ morphologies^35,43^. Similarly, recent authors (e.g., Refs.^1,15,16,38^) have strongly argued for the non-homology of ‘microstomy’, based on fundamental anatomical differences across supposedly ‘microstomatan’ squamates. These findings altogether indicate that the squamate jaw complex may have a much more complicated evolutionary history—including much more widespread convergence—than the traditional paradigm of derived ‘macrostomy’ *versus* plesiomorphic ‘microstomy’ would suggest^1,14–16,36,38,39,42–44^.

However, even though these analyses present numerous arguments regarding squamate evolution, they are all mainly qualitative in nature. Except for a few ancestral state reconstructions (e.g., Refs.^6,12,15,25,38^) and geometric morphometric (GM) analyses (e.g., Refs.^25,30,43,45,46^), snake skull evolution—including the question of jaw structure—has yet to be thoroughly examined from a quantitative anatomical perspective. This is particularly true regarding skull modularity and integration, with only a handful of studies^25,45–49^ examining these phenomena in squamates. One method capable of addressing this gap is the recently developed technique of anatomical network analysis (AnNA)^50^. Based on the mathematical discipline of graph theory, AnNA assesses morphological integration through the lens of organizational modularity; in other words, by assessing patterns of topological connectivity among the components of a complex anatomical system (e.g., patterns of articulations among bones), AnNA ultimately breaks this system down into a series of modules, each comprising a set of components that interact more closely with each other than with the components of other such sets^50–55^ (Fig. 1c). Since its formalization in 2014^50^, AnNA has been used to study modularity across vertebrates, from early tetrapods^56,57^, to archosaurs^58–60^, to synapsids^61–64^; however, snakes have never been analyzed using this method, and only one study has examined squamates in any detail (preprint Ref.^65^).

Indeed, AnNA is in many ways better suited than GM for addressing questions around the evolution of snake skull kinesis and micro- *versus* macrostomy. From an anatomical perspective, the states involved in these conditions typically constitute major rearrangements of various bones and their interarticulations—especially when considering feeding mechanisms^15^—with this spatial reorganization being just as important as changes to the size and shape of individual elements, despite being far less thoroughly studied. From a methodological perspective, AnNA also provides several advantages over GM. For example, the specific identity of the anatomical components being analyzed does not play a role in AnNA, meaning that, in contrast to GM^47,66,67^, this approach to modularity analysis is not affected by assessments of element homology^56^. This is especially important when incorporating bones whose homology is debated (e.g., the angular in anomalepidids, the circumorbital ossifications among various squamates, several skull elements in amphisbaenians; see Refs.^68–70^). Similarly, because AnNA assesses patterns of connectivity independent of element shape or identity, bones that are absent or highly aberrant in some study taxa do not have to be excluded a priori from the overall analysis^57^; in contrast, such structures interfere with the landmark correspondence required for GM, and thus these non-universal landmarks—or the specimens lacking them—would typically have to be excluded from GM-based analyses^66,67,71^. This latter point is particularly salient when studying scolecophidians, due to the drastic variation in the shape and even the fundamental presence/absence of various skull elements across these snakes (see overview in Ref.^15^).

Finally, and most importantly, underlying these methodological differences is the entirely distinct manner in which AnNA- *versus* GM-based analyses of modularity fundamentally conceptualize this phenomenon. As noted above, AnNA reflects the concept of ‘organizational modularity’, with modules being defined for each individual anatomical system based on how—and how thoroughly—the different components of that system interconnect with each other^50,55,72^. Each of these ‘organizational modules’ therefore comprises “a group of elements that establish more and/or stronger interactions within the group than outside it” (Ref.^55^, p. 962), with elements being more ‘integrated’ the more closely they topologically interconnect^50,55^. In contrast, GM-based analyses reflect the notion of ‘variational modularity’^55,72^. Whereas AnNA calculates modules at the level of the individual, this latter approach instead perceives anatomical modularity through the lens of population-level variation, with each ‘variational module’ comprising a set of elements that covary in shape and size across a group of organisms relatively independently of other such sets^55,72^. In this context, the notion of ‘integration’ no longer refers to how strongly elements interact within an individual (as for organizational modularity), but rather to how closely they covary across the ontogeny and/or phylogeny of an overall group^55,72,73^.

Through these conceptual and methodological differences, AnNA therefore provides a complementary but fundamentally distinct perspective on modularity and integration compared to covariation-based analyses of these phenomena^50–52,55,64^. The concepts of mathematical or network-based topology underlying this method in turn evoke classical conceptions of anatomical topography as a fundamental arbiter of homology^50–52,55^ (e.g., topological relations and the ‘Test of Similarity’^74,75^), thus uniquely situating AnNA as a quantitative complement to hypotheses of morphological homology (e.g., the aforementioned debates around jaw evolution). Altogether, AnNA therefore presents a promising avenue for research into the evolutionary morphology not only of squamates—and especially snakes—but indeed of any organism or morphofunctional system comprising complexly articulated structures (cf. e.g., biomechanical modelling of linkage networks within the skulls of fishes^76–78^ and salamanders^79^).

In light of the opportunities afforded by this analytical framework, we therefore present two major hypotheses to be assessed herein. First, following recent arguments of the non-homology of ‘microstomy’ across squamates^1,15,16,38^, we hypothesize that the major groups of ‘microstomatans’ (i.e., non-snake lizards, anilioids, anomalepidids, leptotyphlopids, and typhlopoids; Fig. 1) will exhibit different patterns of skull modularity, particularly in relation to the jaw elements. We test this hypothesis using the network dendrograms produced by AnNA, focusing on the modularity of the upper jaw elements because the mandibles tend to form consistent modules across all vertebrates (cf. e.g., Refs.^58,59^). Furthermore, considering previous suggestions of fossoriality- and miniaturization-driven convergence among scolecophidians^1,15,16,38^—and indeed among squamates more broadly^9,14,15,19,21–34^—we also hypothesize that the overall network architectures recovered herein will carry a signal of such convergence, particularly for the major scolecophidian lineages. We test this hypothesis using the patterns of topospace occupation produced by principal component analysis (PCA) and phylogenetic PCA (pPCA) of various anatomical network parameters. ‘Topospace’ in this context refers to a morphospace based not on raw anatomy, but rather on patterns of connectivity and spatial relations; although equivalent to the “connectivity space” of Ref.^51^, we prefer the present terminology due to its emphasis on the notion of ‘topology’, the core concept linking the mathematical and biological aspects of AnNA^50–52^ (i.e., graph theory and anatomical connectivity, respectively; consider also the centrality of ‘topological connectivity’ in classical assessments of primary homology^74,75,80^).

By applying AnNA to snakes for the first time, this study directly addresses the dearth of quantitative analyses related to the anatomical modularity and evolutionary morphology of this group. Focusing on snakes and especially scolecophidians, this network analysis ultimately provides novel quantitative insight into the anatomy and evolution of the squamate skull.

## Results

### Skull modularity

Our analyses recover each major squamate group as exhibiting a distinctive pattern of skull element connectivity, as described below (Figs. 2, 3, 4, 5, 6, 7, 8; see Supplementary Data File 1 for network adjacency matrices, Supplementary Data File 2 for network analysis R script, Supplementary Figs. S1–S57 for all anatomical network dendrograms, and Supplementary Fig. S58 for labelled network diagrams of the representative skulls from Figs. 2, 3, 4, 5, 6, 7, 8). These different patterns are particularly evident in the connectivity and modularity of the palatomaxillary elements (ectopterygoid, maxilla, palatine, and pterygoid); as such, we preface each subsection below with a brief description of the palatomaxillary anatomy of the group in question (see Ref.^15^ for more detailed comparative anatomical descriptions). Because the goal of this study was to examine ‘microstomatan’ squamates, we focus on these taxa in the ensuing Results and Discussion, but also present preliminary insights regarding ‘macrostomy’ based on our comparative sample of booid-pythonoid and caenophidian snakes.

**Figure 2:**
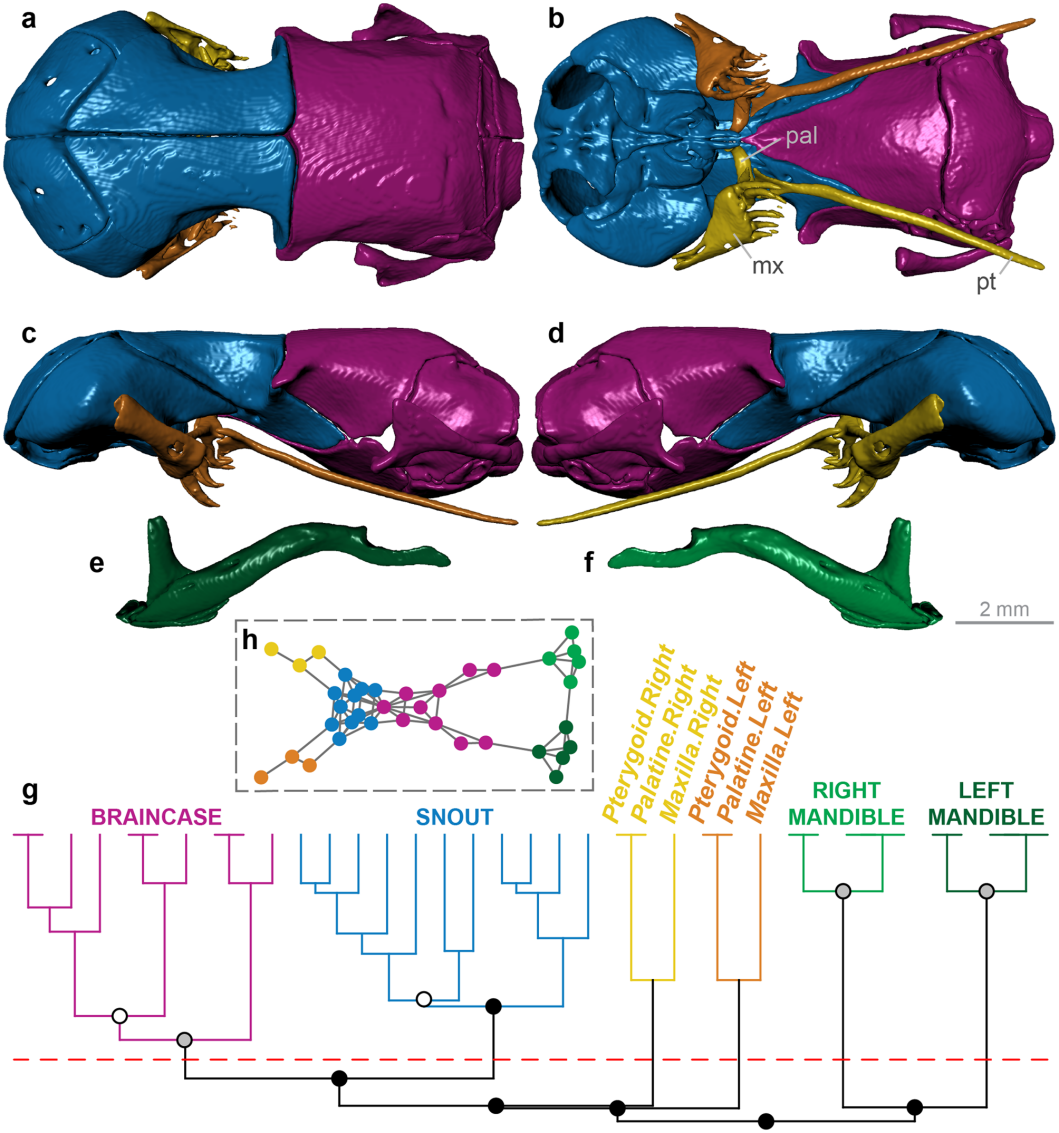
Skull modularity of typhlopoid scolecophidians. Typhlopoids exhibit a highly uniform network structure, including consistent formation of left and right palatomaxillary modules (in italicized boldface). (**a–f**) Typical pattern of typhlopoid skull modularity, illustrated using *Afrotyphlops angolensis* (MCZ R-170385) in (**a**) dorsal, (**b**) ventral, (**c**) left lateral, and (**d**) right lateral views of the skull, and (**e**) left lateral and (**f**) right lateral views of the mandible. (**g**) Network dendrogram of *Xenotyphlops grandidieri* (ZSM 2194/2007), reflecting this general typhlopoid network structure. Q-modules are indicated by Q_max_ (represented by the red dotted line). S-modules are indicated by black (p < 0.001), grey (0.001 ≤ p < 0.01), or white (0.01 ≤ p < 0.05) circles. (**h**) Network representation of the skull of *Xenotyphlops* (see Supplementary Fig. S58 for labelled version). *mx* maxilla, *pal* palatine, *pt* pterygoid. Dendrogram and network generated in R [v.4.0.3]^122^ and RStudio [v.1.3.1093]^123^ (see “Methods” section); specimen visualized in Dragonfly [v.4.1]^114^. MCZ scan data used by permission of the Museum of Comparative Zoology, Harvard University.

**Figure 3:**
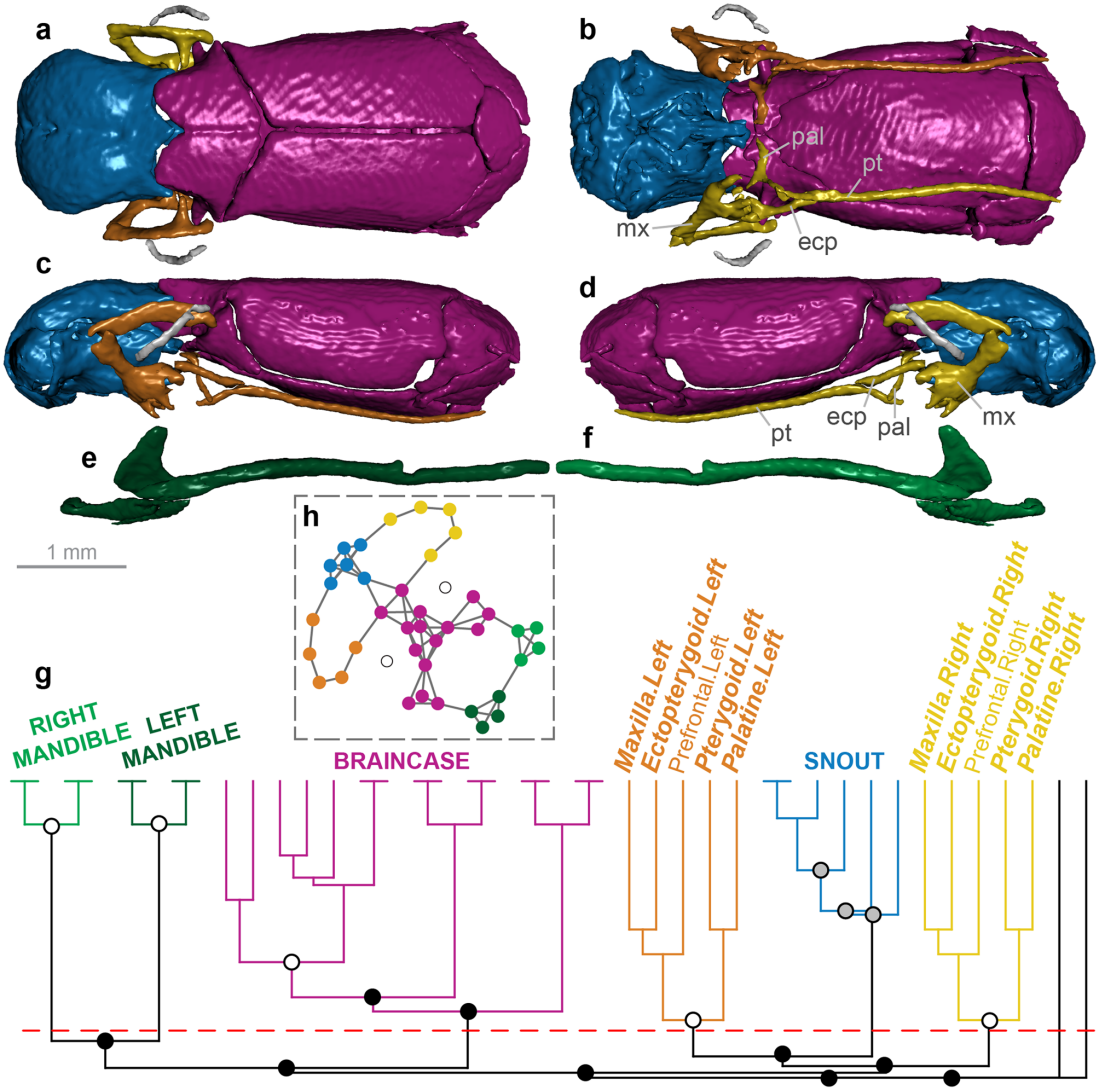
Skull modularity of anomalepidid scolecophidians. In anomalepidids, the ectopterygoids and maxillae always form left and right modules, typically alongside the other palatomaxillary elements (in italicized boldface) and the prefrontals. (**a–f**) Typical pattern of anomalepidid skull modularity, illustrated using *Liotyphlops argaleus* (MCZ R-67933) in (**a**) dorsal, (**b**) ventral, (**c**) left lateral, and (**d**) right lateral views of the skull, and (**e**) left lateral and (**f**) right lateral views of the mandible. (**g**) Network dendrogram of *L. argaleus*, reflecting this general anomalepidid network structure. Q-modules are indicated by Q_max_ (represented by the red dotted line). S-modules are indicated by black (p < 0.001), grey (0.001 ≤ p < 0.01), or white (0.01 ≤ p < 0.05) circles. (**h**) Network representation of the skull of *L. argaleus* (see Supplementary Fig. S58 for labelled version). *ecp* ectopterygoid, *mx* maxilla, *pal* palatine, *pt* pterygoid. Dendrogram and network generated in R [v.4.0.3]^122^ and RStudio [v.1.3.1093]^123^ (see “Methods” section); specimen visualized in Dragonfly [v.4.1]^114^. MCZ scan data used by permission of the Museum of Comparative Zoology, Harvard University.

**Figure 4:**
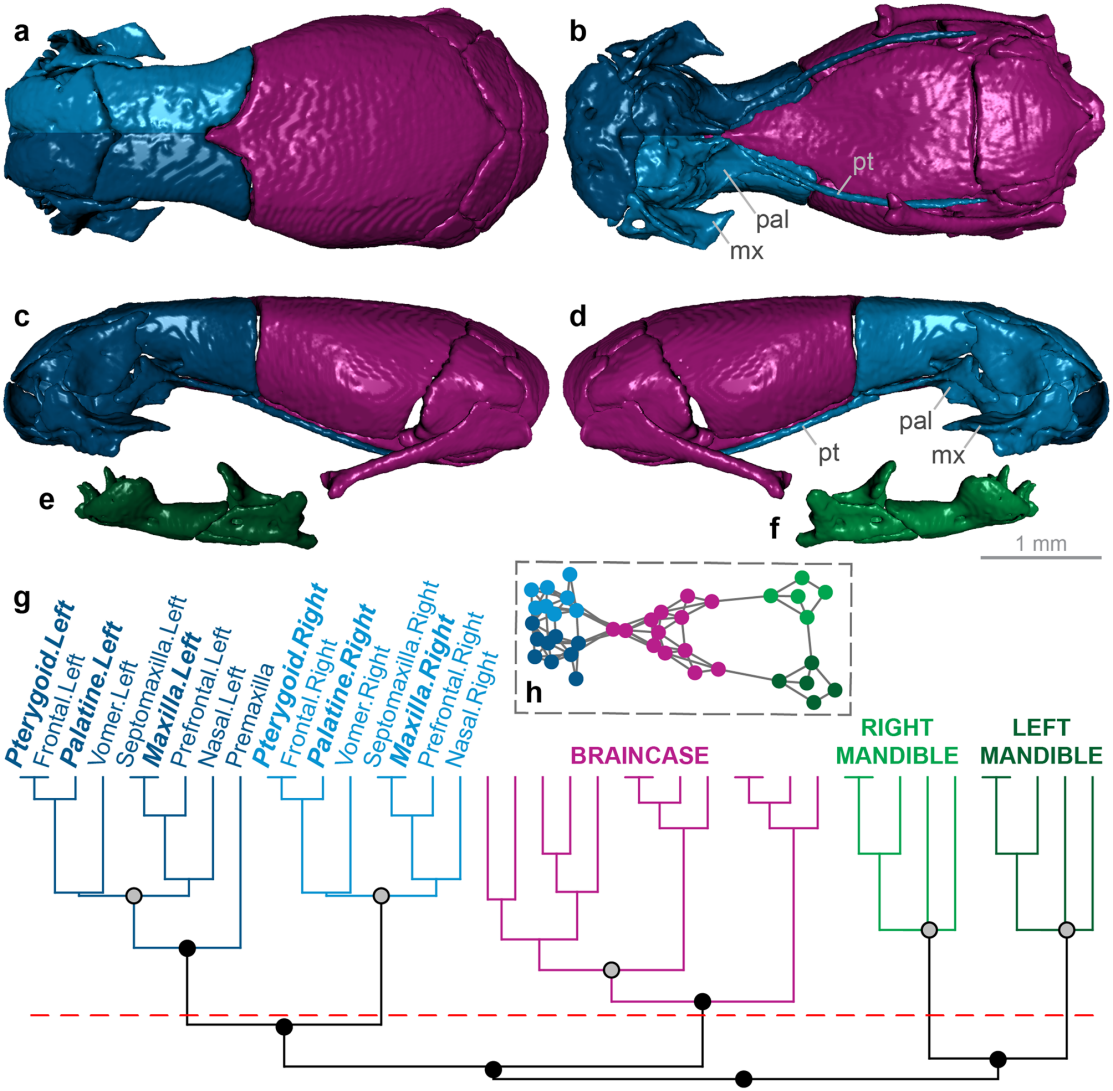
Skull modularity of leptotyphlopid scolecophidians. Leptotyphlopids exhibit a highly consistent pattern of modularity, in which the palatomaxillary elements (in italicized boldface) are thoroughly integrated with the snout. (**a–f**) Typical pattern of leptotyphlopid skull modularity, illustrated using *Epictia albifrons* (MCZ R-2885) in (**a**) dorsal, (**b**) ventral, (**c**) left lateral, and (**d**) right lateral views of the skull, and (**e**) left lateral and (**f**) right lateral views of the mandible. (**g**) Network dendrogram of *Epictia*, reflecting this general leptotyphlopid network structure. Q-modules are indicated by Q_max_ (represented by the red dotted line). S-modules are indicated by black (p < 0.001), grey (0.001 ≤ p < 0.01), or white (0.01 ≤ p < 0.05) circles. (**h**) Network representation of the skull of *Epictia* (see Supplementary Fig. S58 for labelled version). *mx* maxilla, *pal* palatine, *pt* pterygoid. Dendrogram and network generated in R [v.4.0.3]^122^ and RStudio [v.1.3.1093]^123^ (see “Methods” section); specimen visualized in Dragonfly [v.4.1]^114^. MCZ scan data used by permission of the Museum of Comparative Zoology, Harvard University.

**Figure 5:**
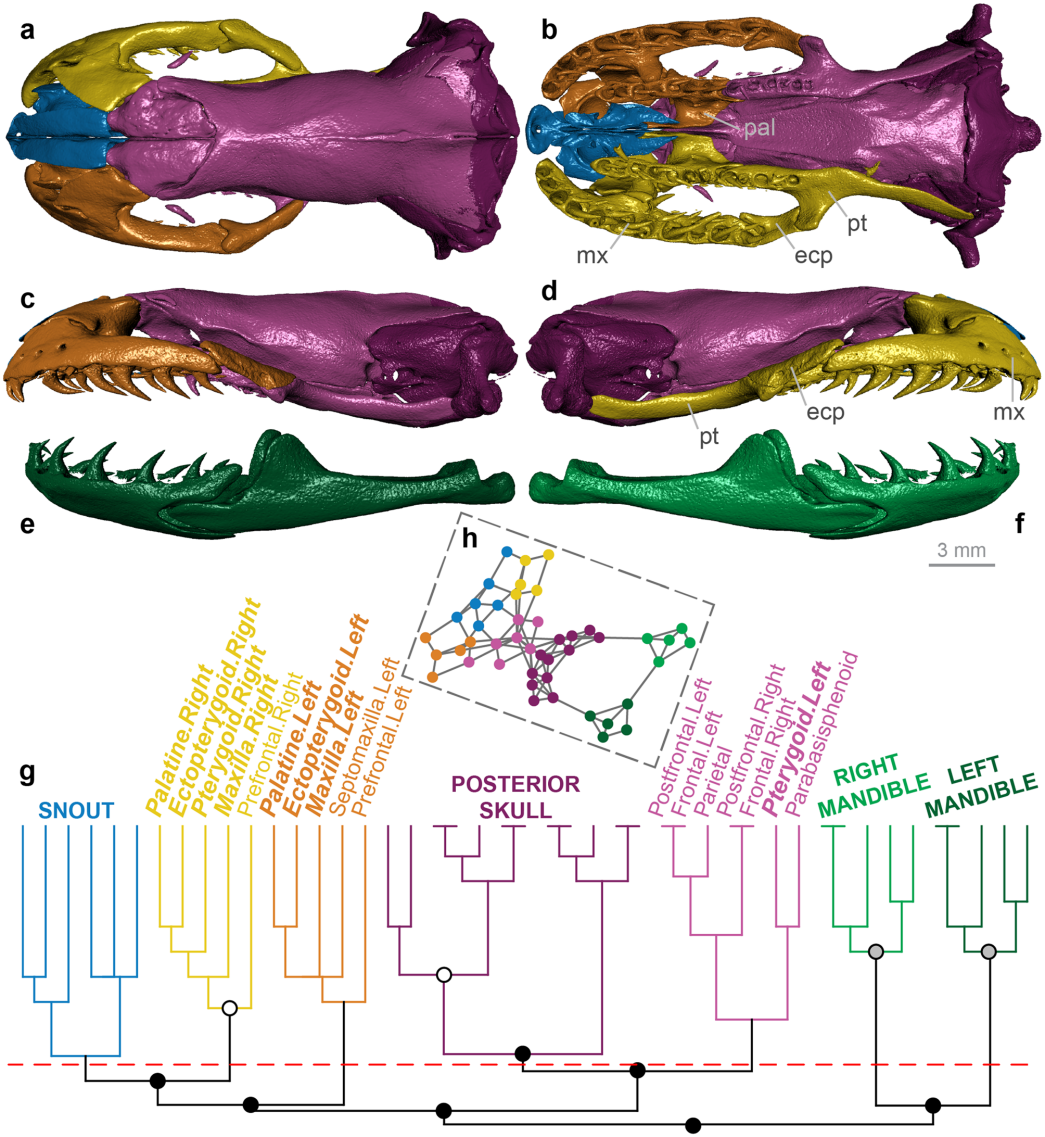
Skull modularity of anilioid snakes. In anilioids, the palatomaxillary elements (in italicized boldface) are integrated to variable extents with the braincase and particularly the snout. (**a–f**) Representative pattern of anilioid skull modularity, illustrated using *Cylindrophis ruffus* (UMMZ 201901) in (**a**) dorsal, (**b**) ventral, (**c**) left lateral, and (**d**) right lateral views of the skull, and (**e**) left lateral and (**f**) right lateral views of the mandible. (**g**) Network dendrogram of *Cylindrophis*, reflecting this network architecture. Q-modules are indicated by Q_max_ (represented by the red dotted line). S-modules are indicated by black (p < 0.001), grey (0.001 ≤ p < 0.01), or white (0.01 ≤ p < 0.05) circles. (**h**) Network representation of the skull of *Cylindrophis* (see Supplementary Fig. S58 for labelled version). *ecp* ectopterygoid, *mx* maxilla, *pal* palatine, *pt* pterygoid. Dendrogram and network generated in R [v.4.0.3]^122^ and RStudio [v.1.3.1093]^123^ (see “Methods” section); specimen visualized in Dragonfly [v.4.1]^114^.

**Figure 6:**
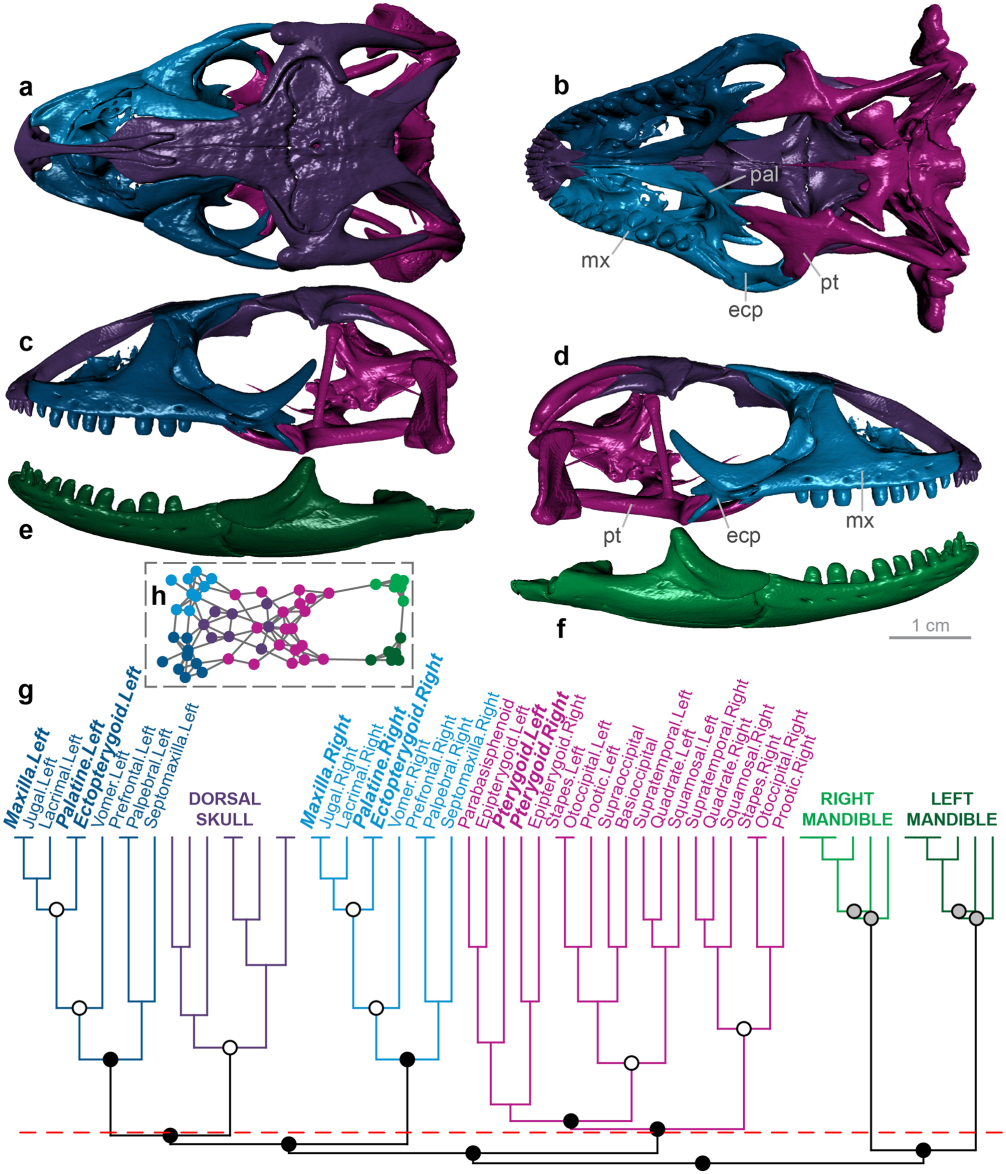
Skull modularity of non-snake lizards. Patterns of skull integration are highly variable among non-snake lizards, with skull regions often being separated across different modules within individual specimens. This is particularly true for the palatomaxillary elements (in italicized boldface), which are typically integrated to some extent with the snout, circumorbital elements, and braincase. (**a–f**) Representative pattern of non-snake lizard skull modularity, illustrated using *Varanus exanthematicus* (FMNH 58299) in (**a**) dorsal, (**b**) ventral, (**c**) left lateral, and (**d**) right lateral views of the skull, and (**e**) left lateral and (**f**) right lateral views of the mandible. (**g**) Network dendrogram of *Varanus*, reflecting this network architecture. Q-modules are indicated by Q_max_ (represented by the red dotted line). S-modules are indicated by black (p < 0.001), grey (0.001 ≤ p < 0.01), or white (0.01 ≤ p < 0.05) circles. (**h**) Network representation of the skull of *Varanus* (see Supplementary Fig. S58 for labelled version). *ecp* ectopterygoid, *mx* maxilla, *pal* palatine, *pt* pterygoid. Dendrogram and network generated in R [v.4.0.3]^122^ and RStudio [v.1.3.1093]^123^ (see “Methods” section); specimen visualized in Dragonfly [v.4.1]^114^.

**Figure 7:**
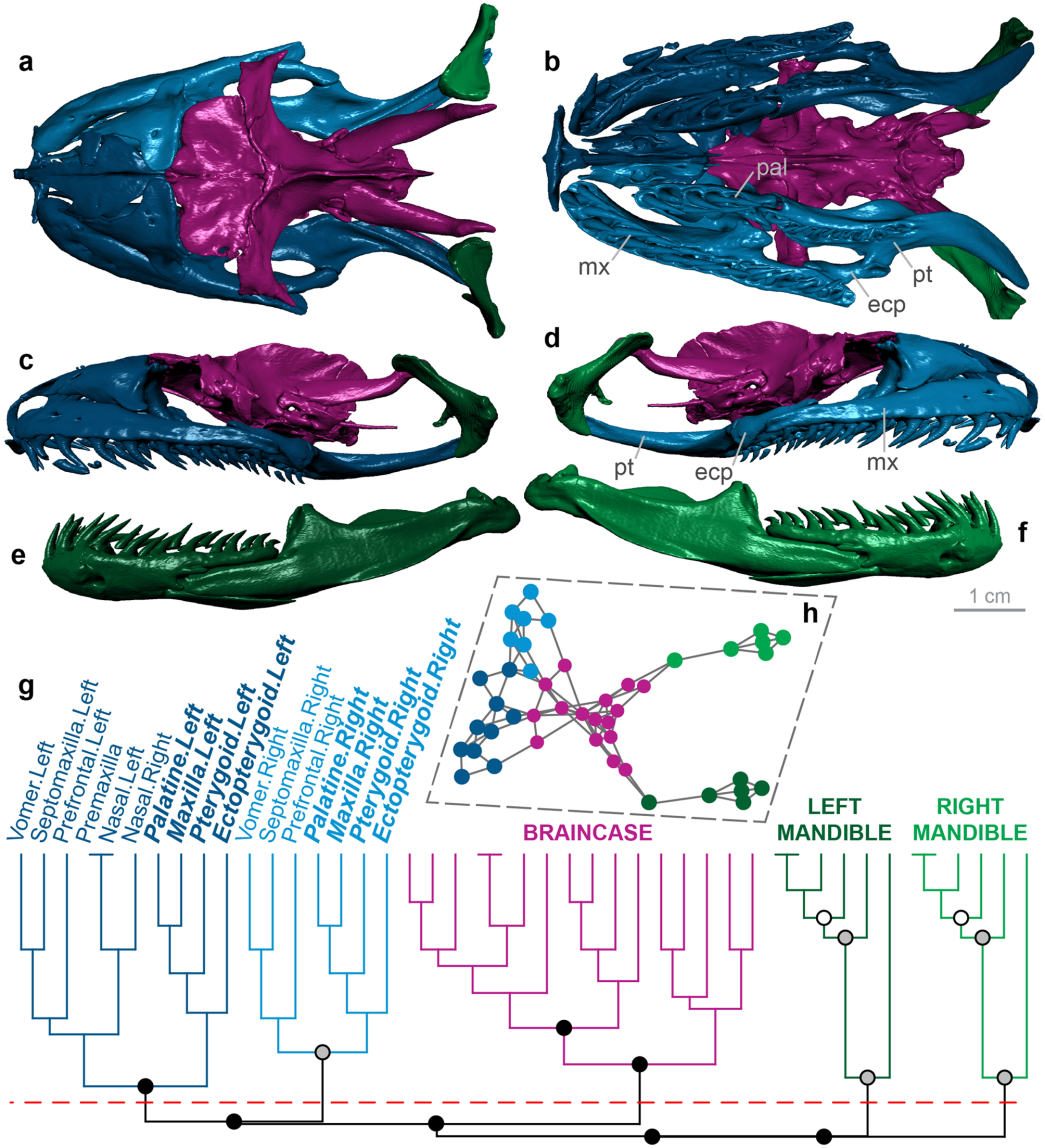
Skull modularity of booid-pythonoid snakes. In booid-pythonoids, the palatomaxillary elements (in italicized boldface) and prefrontals consistently form left and right modules, often alongside some or all of the snout elements. (**a–f**) Representative pattern of booid-pythonoid skull modularity, illustrated using *Boa constrictor* (FMNH 31182) in (**a**) dorsal, (**b**) ventral, (**c**) left lateral, and (**d**) right lateral views of the skull, and (**e**) left lateral and (**f**) right lateral views of the mandible. (**g**) Network dendrogram of *Boa*, reflecting this network architecture. Q-modules are indicated by Q_max_ (represented by the red dotted line). S-modules are indicated by black (p < 0.001), grey (0.001 ≤ p < 0.01), or white (0.01 ≤ p < 0.05) circles. (**h**) Network representation of the skull of *Boa* (see Supplementary Fig. S58 for labelled version). *ecp* ectopterygoid, *mx* maxilla, *pal* palatine, *pt* pterygoid. Dendrogram and network generated in R [v.4.0.3]^122^ and RStudio [v.1.3.1093]^123^ (see “Methods” section); specimen visualized in Dragonfly [v.4.1]^114^.

**Figure 8:**
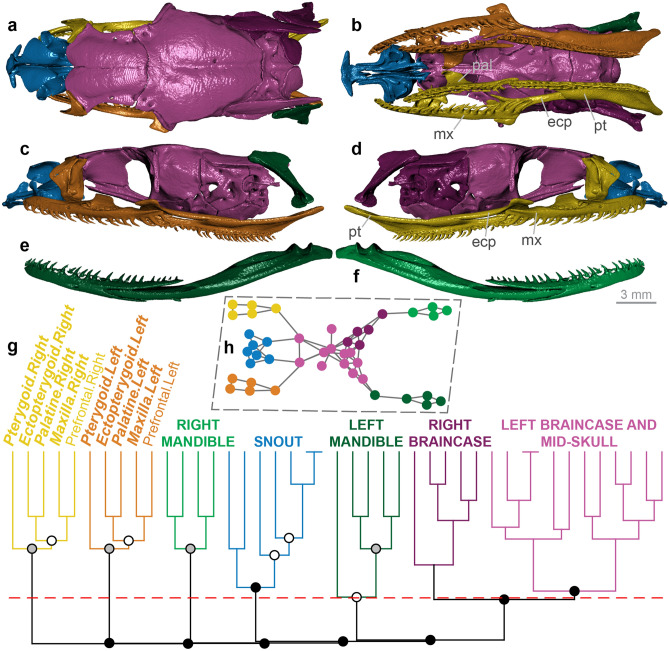
Skull modularity of caenophidian snakes. In caenophidians, the palatomaxillary arches (in italicized boldface) and prefrontals consistently form left and right modules, almost always distinct from all other skull elements (see main text for minor exceptions). (**a–f**) Representative pattern of caenophidian skull modularity, illustrated using *Thamnophis radix* (UAMZ R636) in (**a**) dorsal, (**b**) ventral, (**c**) left lateral, and (**d**) right lateral views of the skull, and (**e**) left lateral and (**f**) right lateral views of the mandible. (**g**) Network dendrogram of *Thamnophis*, reflecting this network structure. Q-modules are indicated by Q_max_ (represented by the red dotted line). S-modules are indicated by black (p < 0.001), grey (0.001 ≤ p < 0.01), or white (0.01 ≤ p < 0.05) circles. (**h**) Network representation of the skull of *Thamnophis* (see Supplementary Fig. S58 for labelled version). *ecp* ectopterygoid, *mx* maxilla, *pal* palatine, *pt* pterygoid. Dendrogram and network generated in R [v.4.0.3]^122^ and RStudio [v.1.3.1093]^123^ (see “Methods” section); specimen visualized in Dragonfly [v.4.1]^114^.

#### Typhlopoidea

Typhlopoid scolecophidians exhibit a unique palatomaxillary configuration in which the maxilla rotates about a rod-like maxillary process of the palatine^15,16,81,82^, forming a ‘single-axle maxillary raking’ mechanism^15^ (Fig. 2a–d). The maxilla is reduced in size and angled transversely, both the palatine and pterygoid are edentulous and structurally simple, and the ectopterygoid is absent^15,16,81,82^ (Fig. 2a–d).

The typhlopoid skull (*n* = 13) typically forms six major modules: the braincase, the snout, the left and right mandibles, and the left and right palatomaxillary arches (Fig. 2; Supplementary Figs. S1–S13). In some taxa (*Afrotyphlops*, *Amerotyphlops*, *Antillotyphlops*, and *Typhlops*; Supplementary Figs. S3, S4, S6, S11), Q_max_ (see “Methods” section) occurs just below this region of the dendrogram, such that the left palatomaxillary elements are included in the snout module; however, in these taxa the snout elements do still form a distinct S-module (p < 0.001; see “Methods” section) to the exclusion of the palatomaxillary elements. This overall pattern is highly consistent across typhlopoids; the only exceptions are *Gerrhopilus ater* (in which the vomers are included in the palatomaxillary modules; Supplementary Fig. S7) and *G. beddomii* (in which the parietal occurs in the snout module, rather than the braincase as in all other typhlopoids; Supplementary Fig. S8).

#### Anomalepididae

In anomalepidid scolecophidians, the maxilla is suspended from the reduced and rod-like prefrontal and braced posteriorly by the ectopterygoid^15,16,69^ (Fig. 3a–d). Despite superficial similarities to typhlopoids (Fig. 2)—e.g., the general structure of the maxilla, palatine, and pterygoid—anomalepidids differ dramatically in the structure of the prefrontal and presence of the ectopterygoid, which together result in a morphofunctionally distinct palatomaxillary configuration termed ‘axle-brace maxillary raking’^15,16^.

The anomalepidid skull (*n* = 6) typically forms eight modules: the braincase, the snout, the left and right mandibles, the left and right jugals, and the left and right ectopterygoid and maxilla (Fig. 3; Supplementary Figs. S14–S19). The ectopterygoid-maxilla module also variably contains the pterygoid (in all taxa except *Typhlophis*), prefrontal (in all taxa except *Anomalepis* and *Liotyphlops beui*), and palatine (in all taxa except *Anomalepis* and *Typhlophis*). The composition of this palatomaxillary module is therefore distinct from that of typhlopoids (Fig. 2; Supplementary Figs. S1–S13), particularly regarding the presence of the ectopterygoid and the inclusion of the prefrontal. When not grouped with the ectopterygoid and maxilla, the prefrontal, palatine, and/or pterygoid are recovered alongside the snout elements (Supplementary Figs. S14, S18, S19). This overall pattern of skull modularity is again quite consistent across anomalepidids, with the only minor exceptions being the formation of a separate vomer-palatine module in *Anomalepis* (Supplementary Fig. S14), the inclusion of the vomer in the palatomaxillary module in *Helminthophis* (Supplementary Fig. S15), and the presence of separate left and right palatine-pterygoid-vomer modules and subdivision of the braincase into three modules in *Typhlophis* (Supplementary Fig. S19).

#### Leptotyphlopidae

Leptotyphlopid scolecophidians are unique among squamates in bearing completely edentulous palatomaxillary arches^15,81,83^ (Fig. 4a–d). In further contrast to other scolecophidians, which rely on highly kinetic upper jaws for prey ingestion, the palatomaxillary elements are essentially immobile in leptotyphlopids: the pterygoid and palatine articulate dorsally with the frontal, the palatine is in broad osseous contact with the vomer, and the maxilla articulates immovably with the snout^15,16,81,83^ (Fig. 4a–d). Leptotyphlopids instead exhibit extensive mandibular kinesis, which reflects a ‘mandibular raking’ mechanism^1,10,15,16,81^. As in typhlopoids (Fig. 2a–d), the ectopterygoid is absent (Fig. 4a–d).

The leptotyphlopid skull (*n* = 6) forms five modules: the braincase, the left and right mandibles, and the left and right snout and palatomaxillary elements (Fig. 4; Supplementary Figs. S20–S25). This pattern of modularity clearly contrasts with the patterns observed in other scolecophidians: whereas typhlopoids (Fig. 2; Supplementary Figs. S1–S13) and anomalepidids (Fig. 3; Supplementary Figs. S14–S19) both exhibit distinct palatomaxillary modules, in leptotyphlopids these elements are always closely integrated with the snout and anterior skull (Fig. 4; Supplementary Figs. S20–S25). These modules are highly consistent across leptotyphlopids, with only *Tricheilostoma* and *Myriopholis tanae* deviating from this pattern. *Tricheilostoma* (Supplementary Fig. S24) differs only in the assignment of the right vomer to the left—rather than right—snout-palatomaxillary module. *M. tanae* (Supplementary Fig. S22) differs more noticeably from other leptotyphlopids, with its braincase exhibiting separate left and right modules and the parietals and parabasisphenoid joining the left snout-palatomaxillary module; however, given the extreme dorsal separation of the skull roof in this taxon (see Ref.^35^, Fig. 2.12B,C for a comparable condition in another leptotyphlopid), this variation in braincase modularity is not unexpected.

#### ‘Anilioidea’ (Aniliidae and Uropeltoidea)

Anilioid snakes (Fig. 5) exhibit slight unilateral movement of the left and right upper jaws, enabled by a unique ‘ball-and-socket’-like maxilla-palatine articulation (Fig. 5b) and by functional decoupling within the snout unit^15,84^. However, this mobility is limited both by the tightness of the ligamentous palatomaxillary-skull connections and by the bracing of the pterygoids against the basipterygoid processes^15,84^ (Fig. 5b). The maxilla, palatine, and pterygoid typically bear teeth, with these elements and the ectopterygoid generally being robust^15^ (Fig. 5a–d). In light of the integral role of the snout elements in the anilioid feeding mechanism, this morphofunctional configuration has been termed ‘snout-shifting’^15,84^.

The anilioid skull (*n* = 5) generally forms six modules: the braincase (sometimes separated across posterior and mid-skull modules), the snout, the left and right mandibles, and the left and right palatomaxillary arches (Fig. 5; Supplementary Figs. S26–S30). However, this pattern of modularity is much more variable than in any scolecophidian clade, as manifested mainly in the palatomaxillary elements and their pervasive integration with the snout and/or braincase. In uropeltids (Supplementary Figs. S29, S30), the ectopterygoid, palatine, and pterygoid form distinct left and right modules, but the maxillae are incorporated into the snout module. In *Anilius* (Supplementary Fig. S26), the snout and palatomaxillary elements are even more closely integrated, with the left palatomaxillary arch grouping with the frontals, parabasisphenoid, left septomaxilla, and left prefrontal, and the right palatomaxillary arch grouping with the premaxilla, nasals, vomers, right septomaxilla, and right prefrontal. *Anomochilus* (Supplementary Fig. S27) shows a similar degree of palatomaxillary-snout integration as in *Anilius*, although the ectopterygoids do not articulate directly with any other element (see Ref.^85^) and thus each form a separate module. Finally, the snout and left and right palatomaxillary arches form generally distinct modules in *Cylindrophis* (Fig. 5; Supplementary Fig. S28), but with notable overlap into other skull regions: the right prefrontal is integrated with the right palatomaxillary arch; the left septomaxilla and prefrontal are integrated with the left palatine, ectopterygoid, and maxilla; and the left pterygoid is integrated with the mid-skull module (comprising the frontals, postfrontals, parietal, and parabasisphenoid).

#### Non-snake lizards

The non-snake lizard skull consists of robust and tightly articulated elements (Fig. 6a–f). This is especially true of the palatomaxillary elements, which—due to their extensive osseous contact with each other and surrounding bones (Fig. 6a–d)—exhibit a much lower degree of mobility than in snakes^84^. In many non-snake lizards, this immobility is reinforced by the presence of elements such as the lacrimal and the degree of development of structures such as the jugal and basipterygoid processes^15^ (Fig. 6a–d). Ultimately, this robust and well-braced jaw configuration reflects ‘minimal-kinesis microstomy’^15^.

The non-snake lizard skull (*n* = 11) broadly separates into five modules: the braincase, the left and right mandibles, and the left and right anterior skull elements (i.e., the snout, palatomaxillary arches, and circumorbital bones) (Fig. 6; Supplementary Figs. S31–S41). However, this pattern is highly variable, with the distinction between these skull regions generally being quite blurred. For example: one or both of the pterygoids are often integrated with the braincase, rather than with the other palatomaxillary elements (e.g., *Dipsosaurus*, *Physignathus*, *Rhineura*, *Sauromalus*, *Uranoscodon*, *Varanus*; Fig. 6; Supplementary Figs. S35, S37–S41); the dorsal skull elements may form a module separate from the snout or braincase (e.g., *Anelytropsis*, *Lanthanotus*, *Sauromalus*, *Uranoscodon*, *Varanus*; Fig. 6; Supplementary Figs. S32, S36, S39–S41); and some (e.g., *Rhineura*; Supplementary Fig. S38) or all (e.g., *Bipes*, *Lanthanotus*; Supplementary Figs. S33, S36) of the snout elements may form a distinct module. Overall, the skull modules are thus much less consistent across taxa and the skull regions are much less distinct within each organism, with the palatomaxillary elements often being separated into different modules alongside the snout, circumorbital elements, and/or braincase.

#### Booidea and Pythonoidea

Booids and pythonoids together form one of the major groups of ‘macrostomatan’ snakes (Fig. 1). Their upper and lower jaw complexes are both highly kinetic^86^, with the palatomaxillary arch bearing particularly strongly recurved teeth (Fig. 7a–f). In booid-pythonoids, the ability to consume proportionally large prey items is achieved mainly via marked posterior elongation of the supratemporal throughout ontogeny, which shifts the jaw articulation posteriorly relative to the skull^35,43^. The quadrate also exhibits positive allometric growth, causing its distal terminus to be displaced laterally throughout development and thus increasing the maximum width of the mouth^43^. Although the basipterygoid processes are typically present (Fig. 7b), they do not directly brace the pterygoids as in anilioids or non-snake lizards (Figs. 5b, 6b; see also Refs.^1,84,86^).

All booid-pythonoids (*n* = 7) exhibit distinct modules for the braincase and left and right mandibles (Fig. 7; Supplementary Figs. S42–S48). However, the remaining skull elements show three different patterns of modularity across this clade. In *Casarea* (Supplementary Fig. S44), *Loxocemus* (Supplementary Fig. S46), and *Python* (Supplementary Fig. S47), the snout, left palatomaxillary arch and prefrontal, and right palatomaxillary arch and prefrontal each form separate modules, as in caenophidians (see below; Fig. 8). The braincase is also subdivided across separate posterior and mid-skull modules, resulting in a total of seven skull modules. In *Boa* (Fig. 7; Supplementary Fig. S42), *Eryx* (Supplementary Fig. S45), and *Xenopeltis* (Supplementary Fig. S48), the snout, palatomaxillary arch, and prefrontal together form left and right modules, resulting in a total of five skull modules. In *Calabaria* (Supplementary Fig. S43), the remaining elements form a dorsal skull module, a left anterior skull module, and a right anterior skull module, resulting in six total modules.

Overall, despite these different patterns, the palatomaxillary arch and prefrontal consistently group together across booid-pythonoid taxa, although are often incorporated with some (*Calabaria*; Supplementary Fig. S43) or all (*Boa*, *Eryx*, *Xenopeltis*; Fig. 7; Supplementary Figs. S42, S45, S48) of the snout elements. This pattern of modularity contrasts the more distinct palatomaxillary arch modules recovered in caenophidians—the other major ‘macrostomatan’ group (see below; Figs. 1, 8; Supplementary Figs. S49–S57)—and the more pervasive palatomaxillary-snout and palatomaxillary-braincase integration typical of anilioids (see above; Fig. 5; Supplementary Figs. S26–S30).

#### Caenophidia

Caenophidians constitute the other major group of ‘macrostomatan’ snakes (Fig. 1). Importantly, though, ‘macrostomy’ arises via a different ontogenetic pathway than in booid-pythonoids^43^. In caenophidians, elongation and rotation of the quadrate throughout ontogeny causes posterior or posterolateral displacement of the quadrate-mandible articulation, whereas the supratemporal typically does not undergo notable posterior elongation^35,43^. Some taxa—e.g., *Homalopsis*, *Thamnophis*—form exceptions to this general caenophidian ontogeny, exhibiting posterior elongation of the supratemporal (especially in *Homalopsis*), as is typical of booid-pythonoids, in addition to the distinct posterolateral orientation of the quadrate as is typical of caenophidians^43,44^. The basipterygoid processes are absent in caenophidians, reflecting an entirely ligamentous or muscular connection between the pterygoid and braincase (see also refs^1,87^ for discussions of the jaw-related musculature in caenophidians).

The caenophidian skull (*n* = 9) is typically arranged into distinct modules for the braincase, snout, left and right mandibles, and left and right palatomaxillary elements and prefrontal (Fig. 8; Supplementary Figs. S49–S57). The braincase is often further split across separate posterior and mid-skull and/or left and right modules (Supplementary Figs. S49, S50, S52–S57). Some variation also arises regarding the snout module: for example, in *Atractaspis* (Supplementary Fig. S51), the palatines form separate left and right modules with the vomers, and the other snout elements group with the mid-skull elements and prefrontals; and, in *Homalopsis* (Supplementary Fig. S53), the left and right vomers are incorporated into the corresponding palatomaxillary-prefrontal modules, as are the jugals (see also *Aparallactus* and *Pareas* for other examples of snout-related variation; Supplementary Figs. S50, S56). Altogether, there is therefore a noticeable degree of variation among caenophidians; however, given the high taxonomic and morphological diversity of Caenophidia—which contains over 2500 species, constituting over 85% of extant snake species^88,89^—such variation is to be expected and is in fact arguably quite minimal given the scope of this clade.

Among caenophidians, the palatomaxillary arches are particularly notable in consistently forming distinct left and right modules alongside the prefrontals (Fig. 8; Supplementary Figs. S49–S57). The only distinct deviation from this palatomaxillary arrangement occurs in *Atractaspis* (Supplementary Fig. S51)—as described above and as is to be expected given the unique palatine-pterygoid separation in atractaspidids^14,90,91^—with minimal deviation in *Homalopsis* (see above; Supplementary Fig. S53) and *Naja* (in which the left prefrontal is incorporated into the mid-skull module; Supplementary Fig. S55). Thus, the palatomaxillary arches in particular show more consistent modularity across caenophidians than across booid-pythonoids (see above).

### Anatomical network parameters and PCA

**Figure 9:**
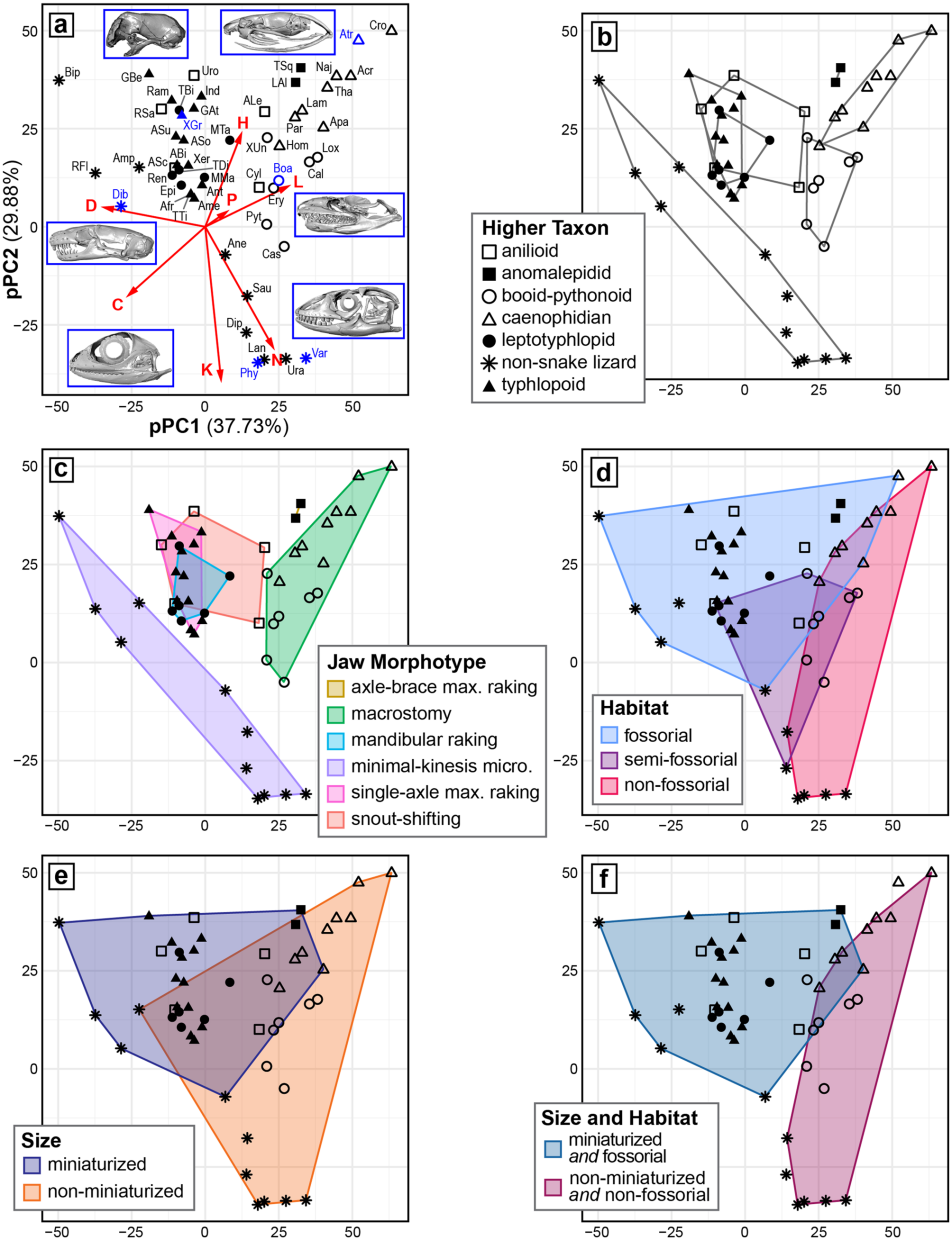
Phylogenetic principal component analysis based on anatomical network parameters. Patterns of topospace occupation are represented using convex hulls. See legend in (**b**) for symbols used throughout all panels. (**a**) Biplot showing overall topospace composition. Red arrows indicate the contribution of each network parameter to the first two phylogenetic principal components. Taxa are labelled using the first three letters of their respective genus, or the first letter of the genus and first two letters of the specific epithet (see Supplementary Table S1). Representative specimens are indicated in dark blue. (**b**) Distribution of higher taxa across topospace. Non-snake lizards, anomalepidids, booid-pythonoids, and caenophidians occupy generally distinct regions, whereas typhlopoids, leptotyphlopids, and anilioids overlap extensively. (**c**) Distribution across topospace of jaw morphotypes proposed by Strong et al.^15^. (**d**) Distribution of habitat types across topospace. Fossorial and non-fossorial taxa occupy distinct regions, although these regions do overlap somewhat. Semi-fossorial taxa occupy an intermediate region overlapping broadly with both other habitats. (**e**) Distribution of size classes across topospace. Miniaturized and non-miniaturized taxa both occupy large regions of topospace; these regions are generally distinct but do exhibit noticeable overlap. (**f**) Distribution of taxa when considering size and habitat simultaneously. Miniaturization and fossoriality together define a more distinct region of topospace than when either phenomenon is considered individually, reflected by reduced overlap between opposing regions (i.e., miniaturized–fossorial *versus* non-miniaturized–non-fossorial). *N* total number of nodes, *K* total number of connections, *D* density of connections, *C* mean clustering coefficient, *L* mean shortest path length, *H* heterogeneity of connections, *P* parcellation. Axis labels are consistent throughout all panels. Analyses performed and plots generated in R [v.4.0.3]^122^ and RStudio [v.1.3.1093]^123^ (see “Methods” section); specimens visualized in Dragonfly [v.4.1]^114^.

Apart from the network dendrograms summarized above, AnNA also calculates several parameters describing various aspects of each anatomical network (Table 1; see “Methods” section for explanation of parameters). In order to further assess skull network diversity across squamates, we analyzed these parameters via pPCA (Fig. 9; Supplementary Figs. S59, S60) and PCA (Supplementary Figs. S61–S63), using various categories to assess the influence of macroevolutionary and adaptational factors such as habitat and body size (see Supplementary Data File 3 for R script, Supplementary Data File 4 for network parameters and groupings, Supplementary Data Files 5 and 6 for pPCA and PCA scores and groupings, Supplementary Table S2 for skull length measurements, Supplementary Tables S3 and S4 for full PERMANOVA statistical results, and Fig. 1b for the phylogeny associated with the pPCA). Both analyses produced very similar results; therefore, although we focus below on the phylogenetically corrected PCA (Fig. 9), the observed patterns also apply to the non-corrected PCA (Supplementary Figs. S61–S63).

**Table 1:** Parameters calculated for each anatomical network.

Taxon	N	K	D	C	L	H	P
*Anilius*	39	91	0.1228	0.4276	3.1134	0.4406	0.7811
*Anomochilus*	41	81	0.0988	0.3181	3.5897	0.5060	0.8209
*Cylindrophis*	45	96	0.0970	0.3664	3.5091	0.4645	0.7714
*Rhinophis*	35	72	0.1210	0.4642	3.1815	0.4633	0.7853
*Uropeltis*	35	68	0.1143	0.3500	3.2118	0.4906	0.7478
*Casarea*	49	107	0.0910	0.4522	4.0765	0.4199	0.7888
*Boa*	45	94	0.0949	0.3760	3.9263	0.4457	0.8148
*Calabaria*	47	95	0.0879	0.2770	3.7299	0.5131	0.7614
*Eryx*	45	95	0.0960	0.3760	3.8758	0.4311	0.8148
*Acrochordus*	43	77	0.0853	0.2257	4.5969	0.5214	0.8069
*Aparallactus*	45	83	0.0838	0.2875	4.2677	0.4999	0.7180
*Atractaspis*	41	67	0.0817	0.1857	4.7512	0.5212	0.7531
*Lampropeltis*	43	81	0.0897	0.2763	4.1030	0.5208	0.6533
*Naja*	43	77	0.0853	0.2123	4.3865	0.5525	0.6555
*Homalopsis*	43	88	0.0975	0.2768	3.8228	0.4486	0.7572
*Thamnophis*	43	75	0.0831	0.2275	4.2182	0.5210	0.6879
*Pareas*	43	79	0.0875	0.3412	4.0853	0.5168	0.7074
*Crotalus*	43	67	0.0742	0.1802	4.8126	0.5611	0.8643
*Loxocemus*	47	95	0.0879	0.2776	3.9112	0.5157	0.7614
*Python*	47	105	0.0971	0.4102	3.7761	0.4399	0.7777
*Xenopeltis*	41	79	0.0963	0.3467	4.1049	0.4085	0.7567
*Anomalepis*	37	59	0.0886	0.4706	3.8807	0.5621	0.8108
*Helminthophis*	40	65	0.0833	0.4479	4.0427	0.5861	0.8125
*Liotyphlops albirostris*	41	68	0.0829	0.4427	4.2186	0.5495	0.8638
*Liotyphlops argaleus*	41	68	0.0829	0.4427	4.2186	0.5495	0.8638
*Liotyphlops beui*	41	67	0.0817	0.4227	4.0472	0.5965	0.8150
*Typhlophis*	40	62	0.0795	0.4211	4.2774	0.5308	0.8638
*Epictia*	40	97	0.1244	0.4735	3.4397	0.4418	0.7725
*Myriopholis macrorhyncha*	41	95	0.1159	0.4773	3.5085	0.4647	0.8245
*Myriopholis tanae*	40	85	0.1090	0.4588	3.8667	0.4758	0.8250
*Rena*	39	95	0.1282	0.4769	3.4345	0.4447	0.7705
*Tricheilostoma*	36	78	0.1238	0.4790	3.5746	0.4903	0.7870
*Trilepida*	39	93	0.1255	0.4634	3.4507	0.4421	0.7771
*Gerrhopilus ater*	35	66	0.1109	0.4776	3.8958	0.4020	0.7739
*Gerrhopilus beddomii*	31	58	0.1247	0.5048	3.5548	0.4138	0.8429
*Acutotyphlops solomonis*	36	72	0.1143	0.5196	4.0127	0.3732	0.7377
*Acutotyphlops subocularis*	36	74	0.1175	0.5272	3.7841	0.4061	0.7824
*Afrotyphlops*	40	89	0.1141	0.5116	3.8705	0.3840	0.6825
*Amerotyphlops*	40	87	0.1115	0.4994	3.9167	0.3513	0.6825
*Anilios*	38	83	0.1181	0.4940	3.6046	0.4033	0.7355
*Antillotyphlops*	40	85	0.1090	0.4941	3.9192	0.3796	0.6825
*Indotyphlops*	35	66	0.1109	0.4554	3.8958	0.4270	0.7739
*Ramphotyphlops*	33	63	0.1193	0.4968	3.9356	0.3787	0.7787
*Typhlops*	40	89	0.1141	0.5116	3.8705	0.3840	0.6825
*Xerotyphlops*	38	81	0.1152	0.4901	3.8094	0.3726	0.7604
*Xenotyphlops*	37	80	0.1201	0.5171	3.4730	0.5314	0.8006
*Amphisbaena*	37	83	0.1246	0.5353	3.1351	0.4288	0.7232
*Bipes*	30	67	0.1540	0.5963	2.8759	0.5380	0.6200
*Rhineura*	36	94	0.1492	0.5658	2.8540	0.4693	0.7701
*Anelytropsis*	47	107	0.0990	0.5212	3.3673	0.4435	0.7759
*Dibamus*	37	90	0.1351	0.5293	3.2583	0.3292	0.7363
*Physignathus*	54	135	0.0943	0.4717	3.3487	0.3561	0.8203
*Dipsosaurus*	52	126	0.0950	0.4861	3.3718	0.3798	0.7633
*Sauromalus*	50	118	0.0963	0.4536	3.4188	0.3924	0.7608
*Uranoscodon*	56	134	0.0870	0.4492	3.4825	0.3774	0.8182
*Lanthanotus*	54	135	0.0943	0.4966	3.5618	0.3624	0.8354
*Varanus*	57	130	0.0815	0.4669	3.9467	0.3636	0.7584

See “Methods” for explanation of parameters.

*N* total number of nodes or elements, *K* total number of connections or articulations, *D* density of connections, *C* mean clustering coefficient, *L* mean shortest path length, *H* heterogeneity of connections, *P* parcellation.

#### Overview of topospace

Phylogenetic principal component (pPC) 1, pPC2, and pPC3 account for 37.73%, 29.88%, and 14.35% of the total variance in the dataset, respectively. pPC1 and pPC2 comprise the focus of the ensuing Results and Discussion, with pPC3 being discussed in the Supplementary Information (see Supplementary Notes; Supplementary Figs. S59, S60).

Taxa toward the lower bound of pPC1 exhibit extensively interconnected skull elements (i.e., high mean clustering coefficient [C] and density of connections [D], low mean shortest path length [L]), whereas taxa toward the upper bound of this axis exhibit less-integrated skull networks (i.e., lower C and D, higher L) (Fig. 9a). In contrast, pPC2 is strongly negatively influenced by N (total number of nodes) and K (total number of connections), such that a lower position along this axis roughly reflects a greater number of skull elements (i.e., higher N) and greater number of total articulations among elements (i.e., higher K) (Fig. 9a).

#### Distribution of higher taxa and jaw morphotypes

We first separated specimens based on higher taxon (Fig. 9b), followed by jaw morphotype (Fig. 9c) as established by Strong et al.^15^ and summarized above. These methods of grouping are equivalent for most specimens, as most of the higher taxa examined herein each exhibit a homologically distinct jaw mechanism^15^ (Fig. 9b,c; Supplementary Data File 4). As an exception, booid-pythonoids and caenophidians occupy distinct regions of topospace but are both ‘macrostomatan’ (Fig. 9b,c; Supplementary Data File 4).

Non-snake lizards (i.e., ‘minimal-kinesis microstomatans’; *n* = 11) occupy the largest region of topospace, comprising taxa with a high number of skull elements and total skull articulations (i.e., ‘typical’ non-snake lizards such as *Varanus* or *Physignathus*) to taxa with fewer, more extensively connected skull elements (i.e., amphisbaenians and *Dibamus*) (Fig. 9a–c). However, despite its size, this region does not overlap with any other higher taxa or jaw morphotypes, highlighting the departure of the snake skull from the pattern of skull integration typical of other squamates (Fig. 9b,c). Anomalepidids (i.e., ‘axle-brace maxillary rakers’; *n* = 2 for pPCA, 6 for PCA) also occupy a distinct region of topospace, reflecting a somewhat loosely integrated skull with relatively few elements (Fig. 9a–c). This region is notably separate from other scolecophidians (Fig. 9b). Both typhlopoids (i.e., ‘single-axle maxillary rakers’; *n* = 13) and leptotyphlopids (i.e., ‘mandibular rakers’; *n* = 6) overlap distinctly with each other and with anilioids (i.e., ‘snout-shifters’; *n* = 5) (Fig. 9a–c). This region reflects a skull structure again with relatively few elements, as in anomalepidids, but with somewhat greater integration among those elements (Fig. 9a–c). Finally, as mentioned above, booid-pythonoids (*n* = 7) and caenophidians (*n* = 9) occupy almost entirely distinct regions of topospace (Fig. 9b); despite both groups exhibiting ‘macrostomy’ (Fig. 9c), caenophidians generally have fewer skull elements and less extensive integration among those elements than booid-pythonoids (Fig. 9a,b).

#### Distribution of habitat types

Fossorial taxa (*n* = 31 for pPCA, 35 for PCA) occupy a large region of topospace, reflecting a relatively low number of skull elements that tend to be more strongly integrated than in non-fossorial taxa (*n* = 15) (Fig. 9d). These fossorial and non-fossorial regions are significantly different (*F*_1,44_ = 19.028, *p* = 0.0001); however, they do exhibit noticeable overlap, mainly due to the placement of the fossorial colubroids *Atractaspis* and *Aparallactus* (Fig. 9a,d). Semi-fossorial taxa (*n* = 7) occupy an intermediate region of topospace, significantly distinct from the fossorial region (*F*_1,36_ = 6.723, *p* = 0.0039) but not the non-fossorial region (*F*_1,20_ = 0.723, *p* = 0.4457) and overlapping strongly with both other habitat types (Fig. 9d).

#### Distribution based on size

Miniaturized taxa (*n* = 30 for pPCA, 34 for PCA) occupy a similar region of topospace as do fossorial taxa, again reflecting skull networks with relatively few and relatively tightly integrated elements (Fig. 9e). However, due to the status of *Atractaspis* as fossorial but not miniaturized (i.e., its exclusion from the current category; Supplementary Data File 4), this region is slightly smaller than that defined by fossoriality (Fig. 9d). Non-miniaturized taxa (*n* = 23) occupy a large region spanning most of topospace, ranging from large networks composed of moderately interconnected elements (many non-snake lizards) to smaller networks of quite strongly interconnected elements (*Amphisbaena*) to much less integrated networks (most caenophidians) (Fig. 9a,e). These miniaturized and non-miniaturized regions are significantly different (*F*_1,51_ = 18.038, *p* = 0.0001), but overlap quite extensively (Fig. 9e).

#### Combined influence of size and habitat

Taxa that are both miniaturized and fossorial (Fig. 9f) occupy a more distinct region of topospace than when these factors are considered independently (Fig. 9d,e). Specifically, miniaturized–fossorial taxa (Fig. 9f; *n* = 29 for pPCA, 33 for PCA) occupy a region equivalent to that delimited by miniaturization alone (Fig. 9e), but smaller than that delimited by fossoriality alone (Fig. 9d). Conversely, non-miniaturized–non-fossorial taxa (Fig. 9f; *n* = 14) occupy a region equivalent to that defined by non-fossoriality alone (Fig. 9d), but smaller than that defined by non-miniaturization alone (Fig. 9e). Thus, when considered simultaneously, these patterns of topospace occupation result in less overlap between contrasting regions (Fig. 9f), which again are significantly different (*F*_1,41_ = 20.822, *p* = 0.0001). In other words, miniaturization and fossoriality together constrain taxa to a comparatively more distinct region of topospace than either does individually.

## Discussion

Although often considered a fundamentally plesiomorphic and homogenous condition among squamates (e.g., Refs.^4,6,37^), recent discussions of ‘microstomy’^1,10,16,81,83^ have emphasized the highly divergent nature of this condition in many taxa, and especially scolecophidians. Most recently, Strong et al*.*^15^ proposed—based on primary homology-centred anatomical assessments of ‘microstomatans’—that ‘microstomy’ in fact exhibits a complex evolutionary history, occurring via five morphofunctionally distinct and non-homologous morphotypes across squamates^15^. The network analyses conducted herein support this hypothesis: rather than the palatomaxillary elements showing consistent patterns of modularity across ‘microstomatans’ (as would be expected if ‘microstomy’ were indeed morphologically homogenous), these elements instead show distinct patterns of connectivity in each ‘microstomatan’ group (Figs. 2, 3, 4, 5, 6), reflecting the various morphofunctional arrangements unique to each of these groups.

Among scolecophidians, the left and right palatomaxillary arches consistently form separate modules in typhlopoids (Fig. 2; Supplementary Figs. S1–S13), as expected under a ‘single-axle maxillary raking’ morphotype^15^. In anomalepidids (Fig. 3; Supplementary Figs. S14–S19), the ectopterygoid and maxilla are universally united into left and right modules—alongside some combination of the palatine, prefrontal, and pterygoid—thus reflecting ‘axle-brace maxillary raking’^15^. In leptotyphlopids, however, the palatomaxillary arches are completely integrated with the snout (Fig. 4; Supplementary Figs. S20–S25), reflecting this clade’s uniquely akinetic palatomaxillary apparatus and resultant reliance on ‘mandibular raking’^10,15,81^.

Among non-scolecophidian ‘microstomatans’, the snout, braincase, and left and right palatomaxillary arches form generally separate modules in anilioids, but often with distinct overlap among these skull regions (Fig. 5; Supplementary Figs. S26–S30). This overlap is particularly evident in the widespread integration of the palatomaxillary elements with the snout (Fig. 5; Supplementary Figs. S26–S30), thus clearly reflecting the reliance of this group on ‘snout-shifting’^15,84^.

On a methodological note, this pattern among anilioids is notable in its recovery of asymmetrical jaw modules, seen most prominently in the differential grouping of the left *versus* right palatomaxillary elements with the braincase in some taxa (Fig. 5; Supplementary Figs. S26, S28). Such asymmetry has been noted by previous authors as an artefact of the inherently hierarchical and dichotomous manner in which AnNA clusters elements into modules; specifically, the presence of unpaired elements or of paired elements with equal affinity to multiple modules causes these modules to be reconstructed asymmetrically, despite the underlying adjacency matrix being perfectly symmetrical (see Refs.^55,59^ for further discussion). However, although this asymmetry is ultimately an artefact, we nonetheless regard it as an informative artefact when interpreted with care. Consider, for example, the contrasting integration in *Cylindrophis* of the right pterygoid with the right palatomaxillary arch and the left pterygoid with the braincase (Fig. 5): rather than simply a meaningless ‘quirk’ of the algorithm, this asymmetry alternatively reflects pervasive integration of the pterygoid with both the braincase and the upper jaw, such that this element can be assigned to either module with equal validity. Indeed, this latter interpretation corresponds well with qualitative assessments of the anilioid jaw mechanism (see Refs.^15,84^), thus echoing previous interpretations (e.g., Refs.^59,92^) that such modular asymmetry can in fact be biologically meaningful. This pattern therefore again reflects the greater palatomaxillary-skull integration central to ‘snout-shifting’ in anilioids^15,84^.

Finally, non-snake lizards exhibit highly variable skull modularity relative to snakes, with extensive overlap between skull regions (Fig. 6; Supplementary Figs. S31–S41). This variation across non-snake lizards is likely at least partially influenced by the taxonomic breadth of this group; however, the overarching lack of definition or modular consistency of skull regions—even within individual specimens—more strongly suggests a genuine lack of modularity corresponding to distinct skull regions. This is consistent with this group’s ‘minimal-kinesis’ morphotype^15^: because the skull elements are pervasively well-braced and universally integrated in the non-snake lizard skull, AnNA ultimately fails to recover the more well-defined modules present in snakes (see also comments above regarding modular asymmetry).

Thus, although AnNA is not itself a test of homology (cf. Refs.^15,74,75,93^, but note that this is an intriguing area for further theorization of this technique; see Refs.^50,51^), these results complement and ultimately support hypotheses of ‘microstomy’ occurring in several evolutionarily distinct forms (as per Refs.^1,15,16,38^): not only is there a lack of primary homology for key character states across ‘microstomatan’ taxa^15^, these proposed morphotypes are indeed sufficiently distinct to result in different patterns of palatomaxillary modularity across these major groups, as demonstrated herein (Figs. 2, 3, 4, 5, 6). As such, ‘microstomy’ should not be considered morphologically homogenous among squamates, nor assumed among snakes to reflect simple retention of an ancestral condition (see also Refs.^1,15,16^); particularly among scolecophidians, these contrasting patterns of modularity instead suggest distinct evolutionary trajectories and clearly non-plesiomorphic conditions in each of these lineages^1,15,16,38^.

When constructing broader hypotheses of scolecophidian evolution, however, it is essential to reconcile the existence of such highly divergent jaw mechanisms with the ostensibly consistent ecomorphology exhibited across these miniaturized and fossorial snakes. Recent authors have discussed this seemingly paradoxical combination of extreme convergence and divergence, ultimately hypothesizing competing roles of contingency and constraint across a highly convergent evolutionary history^1,15,16^. Our analyses of topospace occupation (Fig. 9; Supplementary Figs. S59–S63) provide key quantitative insight into this hypothesis, revealing extensive miniaturization- and fossoriality-associated convergence throughout snakes, and especially scolecophidians. These phenomena have been discussed in the context of squamate comparative anatomy by several authors (e.g., Refs.^9,14–16,18,19,21–24,32–34^), but fossoriality-driven convergence has only recently been examined in GM-based analyses of squamate skull shape^30,33,34^ and integration^25^, and miniaturization-related convergence remains quite underexplored quantitatively.

Regarding habitat, our sample of truly fossorial squamates (dibamids, amphisbaenians, and some snakes) occupies a significantly distinct region of topospace compared to both semi-fossorial and especially non-fossorial squamates, thus reflecting a substantially different skull network architecture (see Results; Fig. 9d). Based on the numerous phylogenies that recover these fossorial taxa as distantly related (e.g., Fig. 1b; Refs.^39,40,94^), we therefore consider their similar network structures to reflect convergence, not phylogenetic affinity (*contra* e.g., Ref.^95^). Generally characterized by a relatively low number of closely integrated skull elements (Fig. 9a,d), this network architecture provides novel quantitative support for previous recognitions of bone loss or reduction, alongside reinforcement of articulations among the remaining elements, as major sources of convergence in fossorial taxa^19,21,22^. It also provides a basis on which to test the degree of specialisation to fossoriality seen in species that have evolved into fossorial forms more recently (e.g., various lineages of skinks^33^).

Miniaturized and non-miniaturized taxa also occupy distinct regions of topospace (see “Results” section; Fig. 9e), reflecting convergence toward a specific skull architecture in miniaturized squamates and especially snakes. This network structure is similar to that in fossorial taxa (Fig. 9d); however, whereas increased connectivity is important in burrowers for mechanical integrity^19,21,22,96^, in miniaturized organisms this greater integration is more likely a consequence of heterochrony and allometric scaling. Allometry has long been recognized as a key source of morphological novelty in miniaturized taxa, with drastic size decrease forcing often-pronounced rearrangements of spatial relationships among affected elements^18,33^. Furthermore, miniaturization has been hypothesized to occur via paedomorphosis^97–101^, an evolutionary developmental phenomenon in which early ontogenetic conditions of an ancestral taxon are retained into the adult stages of a descendant taxon^101,102^. This developmental truncation in turn often involves skeletal reduction, with paedomorphic taxa exhibiting either total absence (e.g., the supratemporal in most scolecophidians and some anilioids^15,24,69^) or extreme reduction (e.g., the supratemporal in most anomalepidids^15,69^) of key skull elements, as is typical of incipient developmental stages (see e.g., Refs.^103–106^). At the same time, paedomorphosis is frequently coupled with hyperossification in vertebrates^18^. Although paedomorphosis also occurs in fossorial taxa (e.g., *Atractaspis*^14^), it is much more pervasive in taxa that are also miniaturized^14,20^ (see below for further discussion); thus, paedomorphic skeletal reduction likely explains the lower number of skull elements in miniaturized compared to non-miniaturized squamates, as well as the greater degree of interconnection among the remaining elements.

The combined influences of fossoriality and miniaturization (see “Results” section; Fig. 9f) further reveal the pressures and constraints shaping squamate skull evolution. Importantly, when these phenomena are analyzed together (Fig. 9f), the opposing regions exhibit even less overlap than when either habitat (Fig. 9d) or size (Fig. 9e) are treated separately. In other words, fossoriality and miniaturization are each associated with a specific set of skull network parameters, constraining fossorial or miniaturized taxa to particular regions of topospace relative to non-fossorial or non-miniaturized taxa, respectively (see above; Fig. 9d,e); however, when these phenomena co-occur, the endpoint categories (i.e., miniaturized–fossorial *versus* non-miniaturized–non-fossorial; Fig. 9f) exhibit even more distinct separation.

Ultimately we cannot entirely disentangle the connection between miniaturization and fossoriality, given that our analysis focuses on scolecophidians and thus lacks a broad sampling of miniaturized but non-fossorial taxa. Nonetheless, from an evolutionary perspective, this comparatively greater constraint is logical given the aforementioned pressures associated with fossoriality and miniaturization. For example, increased connectivity among skull elements is important for structural integrity in fossorial taxa, while also being a consequence of evolutionary size reduction in miniaturized taxa. Taxa that are neither miniaturized nor fossorial face neither of these pressures, whereas those that are both miniaturized and fossorial face both of them, thus even further promoting divergence in network architecture between these categories (see also Refs.^14,20^). Greater network integration is also associated with greater structural inter-dependence among elements, which in turn promotes evolutionary constraint^50,60,64^; thus, trends toward a more interconnected skull architecture in miniaturized or fossorial taxa in turn constrain morphological evolution, essentially generating a feedback cycle that becomes amplified when both of these phenomena are at play.

Altogether, these complementary lines of evidence provide intriguing insight into snake origins and evolution, particularly in terms of recent hypotheses surrounding scolecophidians. Contrary to traditional paradigms of snake evolution (e.g., Refs.^6,37^), recent authors^1,15,16,38,107^ have increasingly argued for the independent evolution of miniaturization, fossoriality, and ‘microstomy’ in each scolecophidian lineage. When considered in tandem, our analyses of skull network composition and topospace occupation provide the first quantitative examination of this heterodox perspective.

Specifically, although typhlopoids and leptotyphlopids overlap in topospace (Fig. 9b,c), their jaw mechanisms (‘single-axle maxillary raking’ *versus* ‘mandibular raking’, respectively) are functionally and anatomically highly divergent (Figs. 2, 4; see also Ref.^15^). In contrast, anomalepidids are separate from other scolecophidians in topospace (Fig. 9b,c), but like typhlopoids are maxillary rakers (although they exhibit ‘axle-brace maxillary raking’, rather than the ‘single-axle’ mechanism of typhlopoids; Figs. 2, 3; see also Ref.^15^). Essentially, typhlopoids and leptotyphlopids are performing different strategies (i.e., maxillary *versus* mandibular raking, non-homologous morphotypes^15^ associated with distinct patterns of modular composition; Figs. 2, 4) within a similar overall network architecture (i.e., overlapping in topospace; Fig. 9b,c); conversely, typhlopoids and anomalepidids are performing superficially similar strategies (i.e., maxillary raking) within highly distinct network structures (i.e., exhibiting different patterns of skull modularity, occupying different regions within the miniaturized–fossorial topospace, and also comprising non-homologous morphotypes^15^; Figs. 2, 3, 9b,c).

When considered in the context of fossoriality, miniaturization, and the interplay between these phenomena, these findings support the aforementioned hypothesis of convergence among scolecophidians. Although scolecophidians do exhibit superficial similarities—such as overlapping occupations of topospace (typhlopoids and leptotyphlopids) or seemingly similar jaw mechanisms (typhlopoids and anomalepidids)—the morphofunctional configurations underlying each of the scolecophidian lineages are in reality strikingly different, with each clade exhibiting a non-homologous^15^ and uniquely modularized (see above; Figs. 2, 3, 4) jaw mechanism. At the same time, because these snakes are fossorial and highly miniaturized, they are therefore restricted to a highly specific region of network topospace among squamates (Fig. 9). In light of this constraint, this observation of superficial similarities masking dramatic underlying differences is thus ultimately most consistent with convergence among the three scolecophidian lineages, driven by the ecological and morphofunctional constraints associated with miniaturization and fossoriality. Applying these findings to the question of snake origins, this study therefore refutes the traditional perspective of scolecophidians as a miniaturized and fossorial vestige of the ancestral snake condition (e.g., Ref.^6^), and instead supports the competing hypothesis of blindsnakes as a highly convergent and morphologically derived assemblage (see also Refs.^1,15,16,38^). These findings thus ultimately reveal how body size and habitat act to influence skull—and especially jaw—anatomy and evolution in blindsnakes.

It is important to emphasize in closing that this study reflects an intentionally snake-, scolecophidian-, and ‘microstomy’-focused investigation; it thus remains an open question as to how the trends observed herein might translate to squamates more broadly. For example, do body size and habitat induce the same separation in topospace, or interact in the same manner, when considering extremely miniaturized but non-fossorial chameleons, or perhaps fossorial but non-miniaturized skinks or gymnophthalmids? What might the inclusion of the fossorial-but-non-serpentiform *Scincus scincus* reveal about possible correlations between postcranial body-plan, fossoriality, and skull architecture (see also Ref.^33^)? And importantly, what might be revealed about snake skull evolution with the inclusion of well-preserved fossil snakes such as *Najash rionegrina*^108^ or *Dinilysia patagonica*^109^?

Ultimately these broader, squamate-wide implications can only be answered via an accordingly squamate-wide analysis. Although such an undertaking lies beyond the scope of this study, our implementation of AnNA—the first of its kind to snakes, and potentially the first among squamates (see preprint Ref.^65^ for the only other squamate-focused network analysis)—clearly reinforces this method as a powerful tool for investigating the structure, function, and evolution of complex anatomical systems. Indeed, when considering future applications of this technique, perhaps the most obvious of such systems—in light of the insights afforded by our inclusion herein of booid-pythonoids and caenophidians—is that of ‘macrostomy’. Specifically, despite both of these groups classically being recognized as ‘macrostomatan’, each shows a distinct pattern of palatomaxillary modularity: caenophidians consistently exhibit discrete left and right palatomaxillary-prefrontal modules, with relatively few exceptions given the size of this clade (see above; Fig. 8; Supplementary Figs. S49–S57), whereas the palatomaxillary modularity of booid-pythonoids is much more variable, with the upper jaw arches sometimes forming separate modules with the prefrontals as in caenophidians, but more often being integrated to some extent with the snout elements (see above; Fig. 7; Supplementary Figs. S42–S48). Because ‘macrostomy’ has not been morphologically re-assessed in the same detail as ‘microstomy’, the broader implications of these differences ultimately remain preliminary; however, in reinforcing previous suggestions of the non-homology of this jaw mechanism^1,39,42–44^, these results strongly emphasize a renewed examination of ‘macrostomy’ as a key avenue for future research.

Comparisons of variational *versus* organizational modularity^55^ represent yet another intriguing topic for future squamate-wide analyses of skull evolution. For example, Watanabe et al*.*^25^ and Rhoda et al*.*^46^ recently assessed cranial integration in snakes via GM-based analyses of shape covariation, incorporating a broad sampling of squamates (including typhlopoids, anilioids, booids, and colubroids) and of aquatic-foraging caenophidians, respectively. Although the details of their results differ, both studies broadly recovered a much more modular arrangement of the snake skull—and notably the palatomaxillary arch—than the present analysis, with Watanabe et al.^25^ also recovering consistent patterns of skull integration across snakes and non-snake lizards^25,46^. The distinct contrast between these findings and ours likely reflects, in no small part, the impact of shape- *versus* connectivity-based analyses of modularity (i.e., analyses of variational *versus* organizational modularity, respectively), thus highlighting the importance of both approaches in future studies of skull integration and modularity (see also Refs.^55,64,72,73^).

## Methods

### Taxon sampling

Because this study aims to assess ‘microstomy’ across squamates, we focused our sampling efforts on the main ‘microstomatan’ groups. As such, we included representatives of every major typhlopoid subclade (2 gerrhopilids, 10 typhlopids, 1 xenotyphlopid), every anomalepidid genus (1 *Anomalepis*, 1 *Helminthophis*, 3 *Liotyphlops*, 1 *Typhlophis*), multiple leptotyphlopid tribes and subtribes as outlined by Adalsteinsson et al*.*^110^ (4 epictines, 2 myriopholines), every anilioid family (1 aniliid, 1 anomochilid, 1 cylindrophiid, 2 uropeltids), and each of the major non-snake lizard groups often hypothesized as the sister-group of snakes (3 amphisbaenians, 2 dibamids, 4 iguanians, 2 varanoids), totalling 41 specimens representing as many species (Figs. 1, 9; Supplementary Table S1). We also included 16 ‘macrostomatans’—i.e., booid-pythonoids (1 bolyeriid, 3 booids, 3 pythonoids) and caenophidians (1 acrochordoid, 8 colubroids)—in order to provide a comparative framework in relation to ‘microstomatans’ and thus more fully establish the squamate topospace (Figs. 1, 9; Supplementary Table S1).

When selecting specimens, we prioritized those that were in good quality (i.e., without major damage or distortion) and in as similar a position as possible (i.e., mouth almost-to-completely closed, with particular attention to the position of the palatomaxillary arches), using multiple conspecific individuals where available if one or both of these conditions were not fully met (see Supplementary Table S1). We further verified specimen quality by ensuring that all exhibited left–right symmetry in skull bone articulations (and thus that the resultant adjacency matrices were symmetrical; see below).

### Network modelling

We modelled each anatomical network by coding each skull into an unweighted and undirected adjacency matrix (Supplementary Data File 1), in which scores of ‘1’ indicate a connection between elements and scores of ‘0’ indicate a lack of connection (see e.g., Refs.^50,55,58^). Although other studies (e.g., Refs.^57,59^) typically consider these connections to represent the sutures or direct physical contacts among bones, this is not a requisite definition; depending on the goal of the analysis and the nature of the question being examined, ‘connections’ could represent any of countless forms of linkage between nodes in the network^55^. Due to the loose overall articulation of the snake skull, bones were herein considered ‘connected’ if in osseous contact or if closely integrated though lacking direct physical contact. In the latter case, we determined whether to score these elements as ‘connected’ or not based on the functional morphology of the bones in question (as informed by personal observations and/or anatomical studies; see references below, especially Refs.^83,84,86,91,111–113^), including the presence of features such as articulatory facets or processes. For example, in snakes, the palatine typically does not directly contact the maxilla, in contrast to the extensive osseous contact typical of non-snake lizards; however, these elements do typically come into close proximity, with one or both bones often bearing processes mediating this junction, which are then ultimately joined by soft-tissue. Given this arrangement—a reflection of the functional interaction of these bones during feeding—it is therefore reasonable to still consider them ‘connected’. This more lenient method of scoring the adjacency matrix is critical when analyzing a highly kinetic structure such as the snake skull, particularly in accurately reflecting patterns of connectivity and functional integration without over-estimating modularity or separation among elements.

Each anatomical network was scored based on direct observation of micro-computed tomography (micro-CT) scans of each specimen (Supplementary Table S1), visualized using Dragonfly [v.4.1]^114^. We performed the scans of MCZ specimens, which will be made available on MorphoSource.org; other scans were obtained from DigiMorph.org and MorphoSource.org (see Supplementary Methods for further information and Supplementary Table S1 for a list of specimens and access information). As noted above, these observations were supplemented with published descriptions of anatomy and functional integration where available; relevant taxa include amphisbaenians^68^, anilioids^24,70,84,85,96,115^, anomalepidids^69,116^, *Atractaspis*^91^, booid-pythonoids^86,115^, *Casarea*^117^, dibamids^22,113^, gerrhopilids^118^, iguanians^113,119,120^, leptotyphlopids^83,121^, typhlopids^2,111^, varanids^104,113^, and xenotyphlopids^16^.

### Anatomical network analysis

All anatomical network analyses were performed in R [v.4.0.3]^122^ and RStudio [v.1.3.1093]^123^, using the packages *igraph* [v.1.2.6]^124^, *ape* [v.5.4-1]^125^, *phytools* [v.0.7-70]^126^, and *XLConnect* [v.1.0.1]^127^ and the core R package *stats*^122^. Our AnNA script (Supplementary Data File 2) is modified from Werneburg et al*.*^58^ and Plateau and Foth^59^, with the parcellation calculation adapted from Esteve-Altava et al*.*^56^. This network analysis algorithm produces two major outputs, as described below, reflecting the modularity and integration of each skull network.

#### Network dendrograms and modular composition

A key output of AnNA is the generation of dendrograms reflecting the pattern of connectivity among each network’s nodes (Figs. 2, 3, 4, 5, 6, 7, 8; Supplementary Figs. S1–S57). For this study, these dendrograms therefore reveal the patterns of articulation among the individual elements in each skull, thus providing the level of anatomical detail required to draw conclusions about the evolution of specific morphofunctional arrangements (e.g., jaw morphotypes). These dendrograms were created using the generalized topological overlap measure (GTOM) introduced by Yip and Horvath^128,129^. This method first converts the aforementioned adjacency matrix into a similarity matrix—i.e., a generalized topological overlap matrix—based on the extent to which each node overlaps with (i.e., connects to the same neighbouring nodes as) each other node^128^. This GTOM matrix is then converted into a dissimilarity or distance matrix, which is in turn analyzed by a hierarchical clustering algorithm—in this case, UPGMA (i.e., unweighted pair group method with arithmetic mean)—to arrange the nodes into a dendrogram. Essentially, nodes with a greater number of shared neighbours have a higher topological overlap than nodes with fewer shared neighbours, are therefore more likely to belong to the same anatomical module, and thus are ultimately recovered closer to each other in the dendrogram than nodes with fewer shared neighbours^50,55,58,59,64,128^.

Once established, each dendrogram must then be partitioned into modules. The main technique used herein for module identification implements the modularity Q-value as introduced by Clauset et al*.*^130^ and Newman and Girvan^131^. This parameter reflects how distinctly the observed modularity varies relative to a randomly-connected network; the Q-value is 0 when the number of connections within modules is no greater than what would be expected under random organization of the overall network, whereas higher Q-values indicate greater connectivity among nodes than expected at random, in turn reflecting a more strongly modular network^50,55,58,59,131^. To determine where to cut the dendrogram, the Q-value was calculated for every possible partition, with the cut-off associated with the highest Q-value (i.e., Q_max_) being considered the preferred partition^50,55,58,64,131^. Modules identified in this manner are herein referred to as Q-modules, as in other AnNA studies (e.g., Refs.^58,59^).

As a supplementary strategy for module detection, each dendrogram was also assessed statistically, using a one-sided two-sample Wilcoxon rank-sum or Mann–Whitney U test. This test evaluates whether the number of internal connections significantly exceeds the number of external connections for every cluster within the dendrogram^55,58,59^. Statistically significant clusters reflect S-modules (sensu e.g., Refs.^58,59^).

#### Anatomical network parameters

The AnNA algorithm also calculates several parameters describing the overall anatomical network in question. We briefly outline these parameters below, and refer the reader to Refs.^50,56^ for further explanation.

The most fundamental components of a network are the nodes (*N*) and the connections linking those nodes (*K*)^50^, as represented by the adjacency matrices described above (Supplementary Data File 1). As in most anatomical network analyses (e.g., Refs.^57–59,62^), *N* herein represents the total number of skull bones in each network. *K* represents the total number of articulations, assessed as described above.

The density of connections (*D*) is the ratio of the actual number of connections in the network to the maximum possible number of connections, thus reflecting how fully-integrated the network is^50^.

The mean clustering coefficient (*C*) is the ratio of the actual number of interconnections among a node’s neighbours to the maximum possible number of inter-neighbour connections for that node, averaged over the entire network^50^.

The mean shortest path length (*L*) is the shortest distance between any pair of nodes, averaged over every pair in the network^50^. *D*, *C*, and *L* together reflect network complexity or co-dependence, as a more thoroughly interconnected or integrated network will have a higher density, higher mean clustering coefficient, and lower mean shortest path length^50,58,64^.

The heterogeneity of connections (*H*) measures the variance in connectivity across the network, reflecting whether all nodes connect to a similar number of neighbours (low H) or whether some nodes have much higher connectivity compared to more isolated nodes (high H)^50,57^. This variance in turn reflects anisomerism, i.e., the extent of imbalance in network structure, with greater heterogeneity typically considered to reflect greater anatomical specialization of the affected nodes^50,58^.

Finally, network parcellation (*P*) is another measure of modularity, using a community detection algorithm to reflect how extensively and how uniformly the network is modularized^56,57,59^. Previous analyses have used the *cluster_spinglass* function in *igraph*^124^ to calculate parcellation; however, this function cannot incorporate isolated elements (e.g., the anomalepidid jugal, which has no articulations), so we instead used a leading eigenvector community detection algorithm as described by Newman^132^ and implemented in *igraph* under the function *leading.eigenvector.community*^124^.

### Principal component analysis

In contrast to the dendrograms generated by AnNA—which provide insight into specific anatomical arrangements within each skull, as mentioned above—the aforementioned network parameters instead provide metrics encapsulating the skull architecture in its entirety for each organism. Comparison of these parameters across taxa is thus essential for examining variation at higher levels of anatomical organization (e.g., convergence in overall skull network construction).

To analyze the network parameters calculated by AnNA, we therefore performed a principal component analysis (PCA; Supplementary Figs. S61–S63; see Supplementary Data File 3 for R script) in R [v.4.0.3]^122^ and RStudio [v.1.3.1093]^123^ using the core R package *stats*^122^ and the package *readxl* [v.1.3.1]^133^. We also performed a phylogenetically corrected PCA (pPCA; Fig. 9; Supplementary Figs. S59–S60) using functions from the packages *geiger* [v.2.0.7]^134^, *phytools* [v.0.7-70]^126^, and *ape* [v.5.4.1]^125^. We used the dated squamate phylogeny of Zheng and Wiens^135^, Appendix S3, matching sampled taxa either to the correct species, or, if they were missing, to a congener (see bolded taxa in Supplementary Data File 4). Where no good proxy was available, the samples were dropped (hence the inclusion of *Anomalepis mexicanus*, *Helminthophis praeocularis*, *Liotyphlops argaleus*, and *L. beui* in the PCA but not the pPCA), and all unsampled tips in the tree were also dropped. We then performed the phylogenetic PCA on the correlation matrix of the data, as the input variables are on different scales.

In order to examine various aspects of squamate macroevolution, we grouped taxa according to several criteria (see below; Supplementary Data Files 4–6). These were visualized using the package *ggplot2* [v.3.3.2]^136^ to create plots and generate normal data ellipses, with the package *ggConvexHull* [v.0.1.0]^137^ being used to generate the convex hulls upon which we based our interpretations (see “Results” section). We assessed the statistical significance of each grouping method via permutational multivariate analysis of variance (PERMANOVA) with 10 000 permutations and using a Euclidean distance matrix. These PERMANOVA tests were performed using the packages *vegan* [v.2.5-6]^138^ and *pairwiseAdonis* [v.0.4]^139^, the latter of which was used to perform pairwise PERMANOVA for groupings with more than two categories (i.e., higher taxon, jaw morphotype, habitat, and combined size-habitat; see below).

#### Higher taxon and jaw mechanism

We first assessed basic patterns of topospace occupation by grouping specimens according to higher taxon (i.e., anilioids, *n* = 5; anomalepidids, *n* = 2 [pPCA] or 6 [PCA]; booid-pythonoids, *n* = 7; caenophidians, *n* = 9; leptotyphlopids, *n* = 6; non-snake lizards, *n* = 11; and typhlopoids, *n* = 13; Supplementary Table S1; Supplementary Data File 4). We then grouped specimens based on the jaw morphotypes proposed by Strong et al*.*^15^ (i.e., axle-brace maxillary raking, *n* = 2 [pPCA] or 6 [PCA]; mandibular raking, *n* = 6; minimal-kinesis ‘microstomy’, *n* = 11; single-axle maxillary raking, *n* = 13; snout-shifting, *n* = 5; and ‘macrostomy’, *n* = 16; Supplementary Data File 4), so as to quantitatively examine this hypothesis of squamate jaw evolution, particularly in terms of which combinations of network parameters characterize each morphotype.

#### Habitat

Based on previous recognitions of extensive fossoriality-driven convergence across squamates (e.g., Refs.^9,14,15,19,21–31,33,34^), we divided specimens according to habitat, with categories for fossoriality (*n* = 31 [pPCA] or 35 [PCA]), semi-fossoriality (*n* = 7), and non-fossoriality (*n* = 15) (Supplementary Data File 4). ‘Fossoriality’ herein refers to taxa that actively burrow (e.g., amphisbaenians^68^) or that have extensively subterranean habits (e.g., the occupation of ant nests by scolecophidians, which are myrmecophagous^16,140,141^; or distinct adaptations for subterranean predation in atractaspidids^14,91^). ‘Semi-fossoriality’ describes taxa that show an affinity for leaf litter or loose soil, but are not strictly tied to subterranean habitats (e.g., *Cylindrophis*, *Loxocemus*^142,143^).

However, as is likely inevitable when assessing a phenomenon as complex as habitat usage, these categories are ultimately arbitrary. As emphasized by Palci et al*.*^143^, many taxa do not in reality strictly conform to idealized ecological categories (e.g., accounts of arboreality in the classically fossorial scolecophidians^144^), with this ambiguity further exacerbated by a dearth of rigorous field studies of squamate—and particularly scolecophidian—ecology^140,145^. The definitions above therefore provide a general—but by no means definitive—guideline for demarcating habitat type and its influence on morphological evolution.

Habitat designations for most snake taxa are based on Figueroa^146^, Table S3.1. The scolecophidian genera *Antillotyphlops*, *Anomalepis*, *Helminthophis*, and *Tricheilostoma* were not included in Figueroa’s^146^ analysis, so were instead assigned to habitat types based on phylogenetic bracketing. We also used various literature sources to designate habitat types for sampled non-snake lizards (amphisbaenians^68^, dibamids^22^, *Dipsosaurus*^147^, *Lanthanotus*^148^, *Physignathus*^149^, *Sauromalus*^150^, *Uranoscodon*^151^, and *Varanus*^152^).

#### Size

Like fossoriality, miniaturization has also been proposed as a major source of convergence in squamates (e.g., Refs.^9,14–16,18,23,24,32^) and is thus a phenomenon worth examining herein. However, as is often the case in vertebrates^18^, there is no set guideline or measurement for what constitutes ‘miniaturization’ in squamates (similar to the issue noted above regarding guidelines for determining fossoriality *versus* semi-fossoriality). For the present study, we assigned taxa to size categories by measuring the snout-occiput length of each specimen (either directly from micro-CT scans or from the images available on DigiMorph.org), plotting these values, and looking for breaks in the distribution (Supplementary Table S2). For the observed specimens, skull length increases by about 1 mm or less between taxa until a length of 11.74 mm, after which the next value is 14.05 mm. After this point, skull length varies more distinctly among specimens. Based on this distribution, taxa with skull lengths ≤ 11.74 mm were considered ‘miniaturized’ (*n* = 30 [pPCA] or 34 [PCA]), whereas those with skull lengths ≥ 14.05 mm were considered ‘non-miniaturized’ (*n* = 23) (Supplementary Data File 4; Supplementary Table S2).

#### Size and habitat

Fossoriality and miniaturization often co-occur in squamates, and their respective influences can be quite complexly intertwined^14,20,22–24,98,153^. Furthermore, the interaction between these phenomena has been hypothesized to exert a strong influence on squamate evolution and anatomy^14^. To examine this potential interplay, and to enable comparison of this combined influence to the patterns of topospace occupation that arise when these phenomena are considered separately (see above), as our final analysis we divided taxa into three categories based on their categorization under preceding variables: those that are both miniaturized and fossorial (*n* = 29 [pPCA] or 33 [PCA]), those that are neither miniaturized nor fossorial (*n* = 14), and those that are either miniaturized or fossorial but not both (*n* = 10) (Supplementary Data File 4). Focusing on the end-point categories (i.e., miniaturized–fossorial *versus* non-miniaturized–non-fossorial), we compared this plot to those generated by habitat or size alone, so as to assess relative patterns of topospace occupation.

## Supplementary Information

Supplementary Information 1.

Supplementary Information 2.

Supplementary Information 3.

Supplementary Information 4.

Supplementary Information 5.

Supplementary Information 6.

Supplementary Information 7.

## Data Availability

The micro-CT scans used in the present study are available as indicated in the Supplementary Methods and Supplementary Table S1. All other relevant data and code generated or analysed during this study are included in this article and its Supplementary Information files.

## Code availability

All relevant computer code is provided in the Supplementary Information (see Supplementary Data Files 2, 3).
