# Supplementary material for: Convergence, divergence, and macroevolutionary constraint as revealed by anatomical network analysis of the squamate skull, with an emphasis on snakes
Source: Sci Rep. 2022 Aug 25;12:14469. doi: 10.1038/s41598-022-18649-z (PMC9411180; doi:10.1038/s41598-022-18649-z)
Supplement: Supplementary file 1 — Supplementary Information 1. [file 41598_2022_18649_MOESM1_ESM.pdf]

Supplementary Materials for

Convergence, divergence, and macroevolutionary constraint as revealed by anatomical network analysis of the squamate skull, with an emphasis on snakes

Catherine R. C. Strong*, Mark D. Scherz, and Michael W. Caldwell

*Corresponding author. Email: crstrong@g.harvard.edu

This PDF file includes:

SUPPLEMENTARY METHODS **3**

Sources of micro-CT scan data 3

SUPPLEMENTARY NOTES **5**

Supplementary Results: Trends in topospace as revealed by pPC3 5

SUPPLEMENTARY FIGURES **7**

Supplementary Figures S1–S57. Anatomical network dendrograms 7
Supplementary Figure S58. Anatomical networks of representative squamates 66
Supplementary Figure S59. Phylogenetic principal component analysis based on anatomical network parameters, depicting pPC1 and pPC3 68
Supplementary Figure S60. Phylogenetic principal component analysis based on anatomical network parameters, depicting pPC2 and pPC3 70
Supplementary Figure S61. Principal component analysis based on anatomical network parameters, depicting PC1 and PC2 72
Supplementary Figure S62. Principal component analysis based on anatomical network parameters, depicting PC1 and PC3 74
Supplementary Figure S63. Principal component analysis based on anatomical network parameters, depicting PC2 and PC3 76

SUPPLEMENTARY TABLES **77**

Supplementary Table S1. List of specimens analyzed in this study 77
Supplementary Table S2. Measurements of skull length (mm) in observed taxa 81
Supplementary Table S3. PERMANOVA statistical results for pPCA 83
Supplementary Table S4. PERMANOVA statistical results for PCA 87

REFERENCES – SUPPLEMENTARY INFORMATION **91**

This article is also accompanied by the following supplementary data files:

Supplementary Data File 1. Adjacency matrices used for anatomical network analysis.

Supplementary Data File 2. R script used for anatomical network analysis.

Supplementary Data File 3. R script used for principal component analyses.

Supplementary Data File 4. Network parameters and groupings used for principal component analyses.

Supplementary Data File 5. Phylogenetic-PCA scores and groupings.

Supplementary Data File 6. PCA scores and groupings.

Supplementary Methods

Sources of micro-CT scan data

All micro-CT scans of MCZ specimens were performed by CRCS and will be made available on MorphoSource.org, accompanied by the relevant metadata. Copyright of all MCZ scans belongs to the Museum of Comparative Zoology, Harvard University, and the associated raw digital media are © President and Fellows of Harvard College, 2020, all rights reserved. These are used herein with permission.

Several scans were obtained from DigiMorph.org, as provided by the University of Texas High-Resolution X-ray CT Facility (UTCT). Scans of YPM 14378 and YPM 14376 were originally collected under NSF grants DEB-0132227, EF-0334961, and IIS-9874781. Scans of FMNH 58299, FRIM 0026, FMNH 216257, FMNH 148589, FMNH 22468, UMMZ 190285, and FMNH 95184 were collected under NSF grants IIS-0208675 and EF-0334961. Scans of FMNH 62204, FMNH 63117, FMNH 117833, FMNH 104800, and FMNH 148900 were collected under NSF grant EF-0334961. Scans of YPM 12871 were collected under NSF grants EF-0334961 and IIS-9874781. Scans of TNHC 18483 were collected under NSF grant IIS-9874781. Scans of TNHC 62769 were collected under NSF grant IIS-0208675. Scans of FMNH 167048 were also obtained from DigiMorph.

Several other scans were downloaded from MorphoSource.org, Duke University. The University of Michigan Museum of Zoology provided access to the data for UMMZ 201901 (media ID 000070987), the collection of which was funded by oVert TCN under NSF DBI-1701714 and NSF DBI-1701713. The Florida Museum of Natural History at the University of Florida provided access to the data for UF 33488 (media ID 000062342), UF 78397 (media ID 000072220), UF 103268 (media ID 000048786), and UF 68168 (media ID 000072081), the collection of which was funded by oVert TCN under NSF DBI-1701714, and for UF 143722 (media ID 000445119), the collection of which was funded by oVert TCN under NSF DBI-1701737. The University of Kansas Center for Research Inc provided access to the data for KUH 125976 (media ID 000075015) and KUH 116885 (media ID 000075008), the collection of which was funded by oVert TCN under NSF DBI-1701714, NSF DBI-1701713, and NSF DBI-1701932. oUTCT provided access to the data for FMNH 22847 (media ID 000098383), FMNH 31182 (media ID 000098393), TCWC 45501 (media ID 000113753), CAS 126478 (media ID 000098391), FMNH 31774 (media ID 000098600), FMNH 259340 (media ID 000097478), and

FMNH 128304 (media ID 000098586), originally appearing in Gauthier *et al.*[1], with data collection funded by NSF EF-0334961 and data upload to MorphoSource funded by DBI-1902242. Mark D. Scherz provided access to the data for ZSM 2194/2007 (media ID 000079510), originally appearing in Chretien *et al.*[2].

Finally, scans from the AMS and SAMA collections were provided courtesy of A. Palci, and scans of UAMZ specimens were provided courtesy of lab colleagues.

Supplementary Notes

Supplementary Results: Trends in topospace as revealed by pPC3

As discussed in the main text, pPC1 and pPC2 together reveal that fossoriality and miniaturization each characterize a distinct region of topospace, with their combined influence reflecting even further constraint on skull architecture relative to non-miniaturized–non-fossorial squamates (Fig. 9). Comparison of each of these axes of variation to pPC3 (Supplementary Figs S59–S60) also provides insight into the effect of habitat and body size on squamate skull architecture, extending these findings by revealing the influence of anatomical network parameters not captured by the first two components. As for the first two axes of variation, pPCA and PCA produced similar trends in relation to their respective third components; therefore, as in the main text, the ensuing results and interpretations feature pPC3 (Supplementary Figs S59–S60) but are also largely applicable to PC3 (Supplementary Figs S62–S63).

Comparison of pPC1 to pPC3 (Supplementary Fig. S59) closely matches the trends discussed in the main text (cf. Fig. 9), with combined analysis of habitat and body size again causing reduced overlap between end-point categories (i.e., miniaturized-fossorial *versus* non-miniaturized–non-fossorial; Supplementary Fig. S59f) compared to when these categories are considered separately (Supplementary Fig. S59d–e). However, comparison of pPC2 to pPC3 (Supplementary Fig. S60) provides a slightly different pattern. As for pPC1 *versus* pPC2, miniaturized–fossorial taxa (Supplementary Fig. S60f) occupy a region equivalent to that delimited by miniaturization alone (Supplementary Fig. S60e) but smaller than that delimited by fossoriality alone (Supplementary Fig. S60d), and non-miniaturized–non-fossorial taxa (Supplementary Fig. S60f) occupy a region equivalent to that defined by non-fossoriality alone (Supplementary Fig. S60d) but smaller than that defined by non-miniaturization alone (Supplementary Fig. S60e). However, whereas this pattern causes a reduction in overlap for pPC1 *vs* pPC2 (Fig. 9f), this is not the case for pPC2 *vs* pPC3 (Supplementary Fig. S60f). Instead, along the latter axes of variation, the miniaturized-fossorial *versus* non-miniaturized–non-fossorial regions are arranged such that they still overlap quite heavily, to the same extent as when considering miniaturization alone (Supplementary Fig. S60e–f). These axes therefore do not provide as strong a signal for the influence of habitat and/or body size as that encapsulated by pPC1 *vs* pPC2 (Fig. 9).

In interpreting this difference, it is important to consider the various parameters driving each of pPC1, pPC2, and pPC3, with particular attention to their respective anatomical implications. Specifically, the distribution of taxa within the pPC1–pPC2 topospace (Fig. 9) is driven largely by density [D], mean clustering coefficient [C], and mean shortest path length [L] (along pPC1), and by the total numbers of skull elements [N] and articulations [K] (along pPC2). Notably, both of these sets of parameters bear clear anatomical implications (i.e., reflecting how thoroughly the skull is internally connected, and how large the overall network is, respectively), with intuitive relevance for both fossoriality and miniaturization (e.g., see Discussion in main text regarding structural reinforcement, paedomorphic skeletal reduction, and allometric scaling in relation to these parameters). In contrast, pPC3 is driven almost entirely by parcellation [P], a metric describing the overall 'level of modularity' within a given network (i.e., how many modules exist and how evenly nodes are distributed among those modules[3]). Unlike the parameters driving pPCs 1 and 2, this latter metric does not bear such obvious anatomical relevance to the topics of habitat and body size; in other words, variation in parcellation does not confer an immediately evident adaptive advantage (unlike variations in D/C/L, which reflect structural reinforcement), nor is it as directly related to developmental phenomena like allometry or paedomorphosis (as discussed in the main text for D/C/L in the context of miniaturization, and N/K in the context of skeletal reduction, respectively). It is therefore perhaps unsurprising that a topospace based on pPC2 and pPC3—and thus excluding the information carried by pPC1—does not bear as strong a signal regarding the interplay between fossoriality and miniaturization. In light of these findings, it will therefore be important for future research to clarify the anatomical and adaptive implications of 'parcellation', so as to better understand how—and, in a broader evolutionary sense, why—this parameter varies across taxa.

Supplementary Figures

Supplementary Figures S1–S57. Anatomical network dendrograms

Dendrograms reflecting the anatomical network structure and modular composition of each specimen analyzed in this study. Q-modules are indicated by Q_{max} (represented by the red dotted line). S-modules are indicated by black ($p < 0.001$), grey ($0.001 \leq p < 0.01$), or white ($0.01 \leq p < 0.05$) circles. The palatomaxillary elements are indicated in italicized boldface.

Supplementary Figure S58. Anatomical networks of representative squamates

Supplementary Figure S59. Phylogenetic principal component analysis based on anatomical network parameters, depicting pPC1 and pPC3

Supplementary Figure S60. Phylogenetic principal component analysis based on anatomical network parameters, depicting pPC2 and pPC3

Supplementary Figure S61. Principal component analysis based on anatomical network parameters, depicting PC1 and PC2

Supplementary Figure S62. Principal component analysis based on anatomical network parameters, depicting PC1 and PC3

Supplementary Figure S63. Principal component analysis based on anatomical network parameters, depicting PC2 and PC3

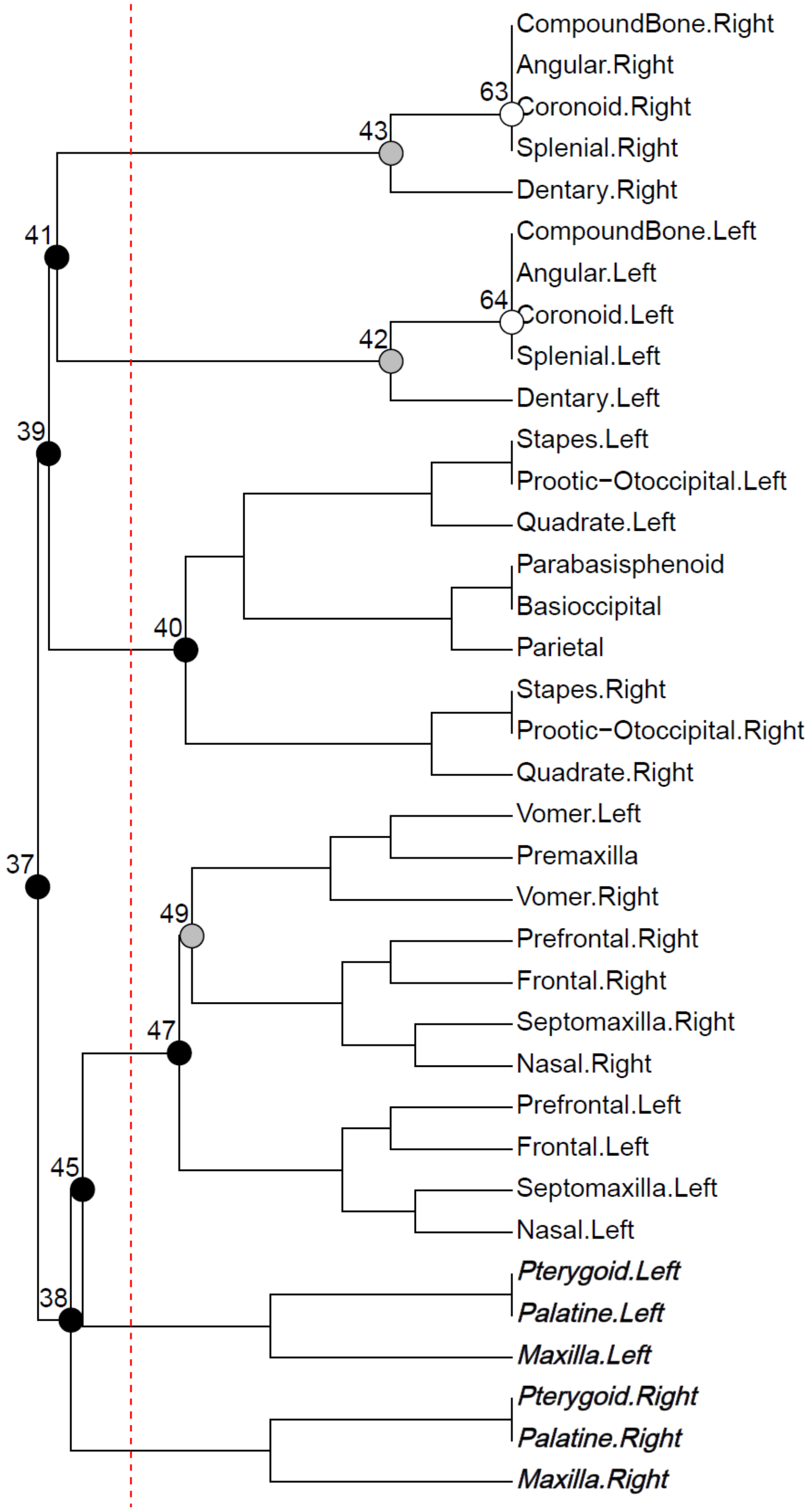

Supplementary Figure S1. Modularity of the skull network of *Acutotyphlops solomonis* (AMS R11452).

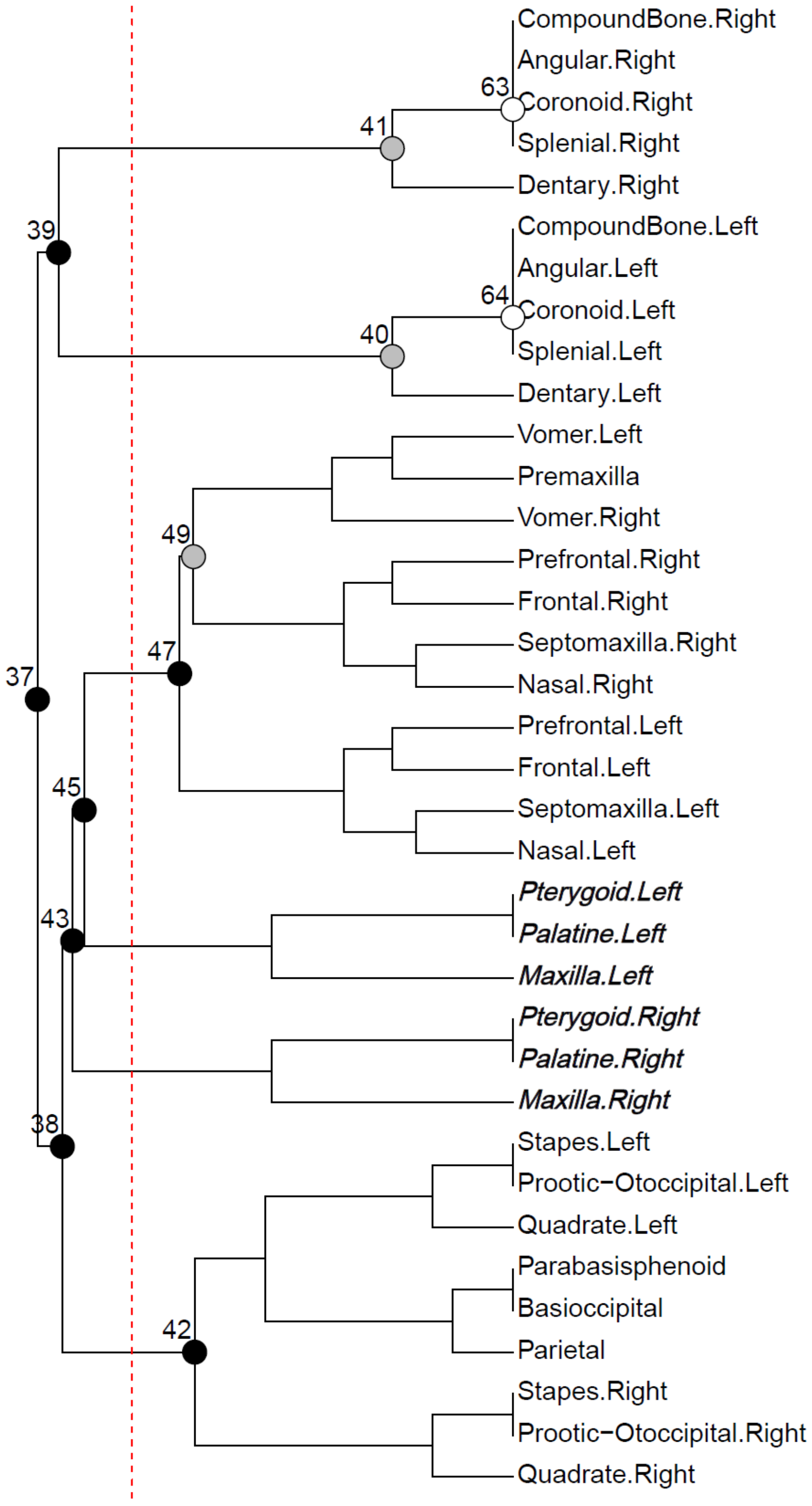

Supplementary Figure S2. Modularity of the skull network of *Acutotyphlops subocularis* (SAMA R64770).

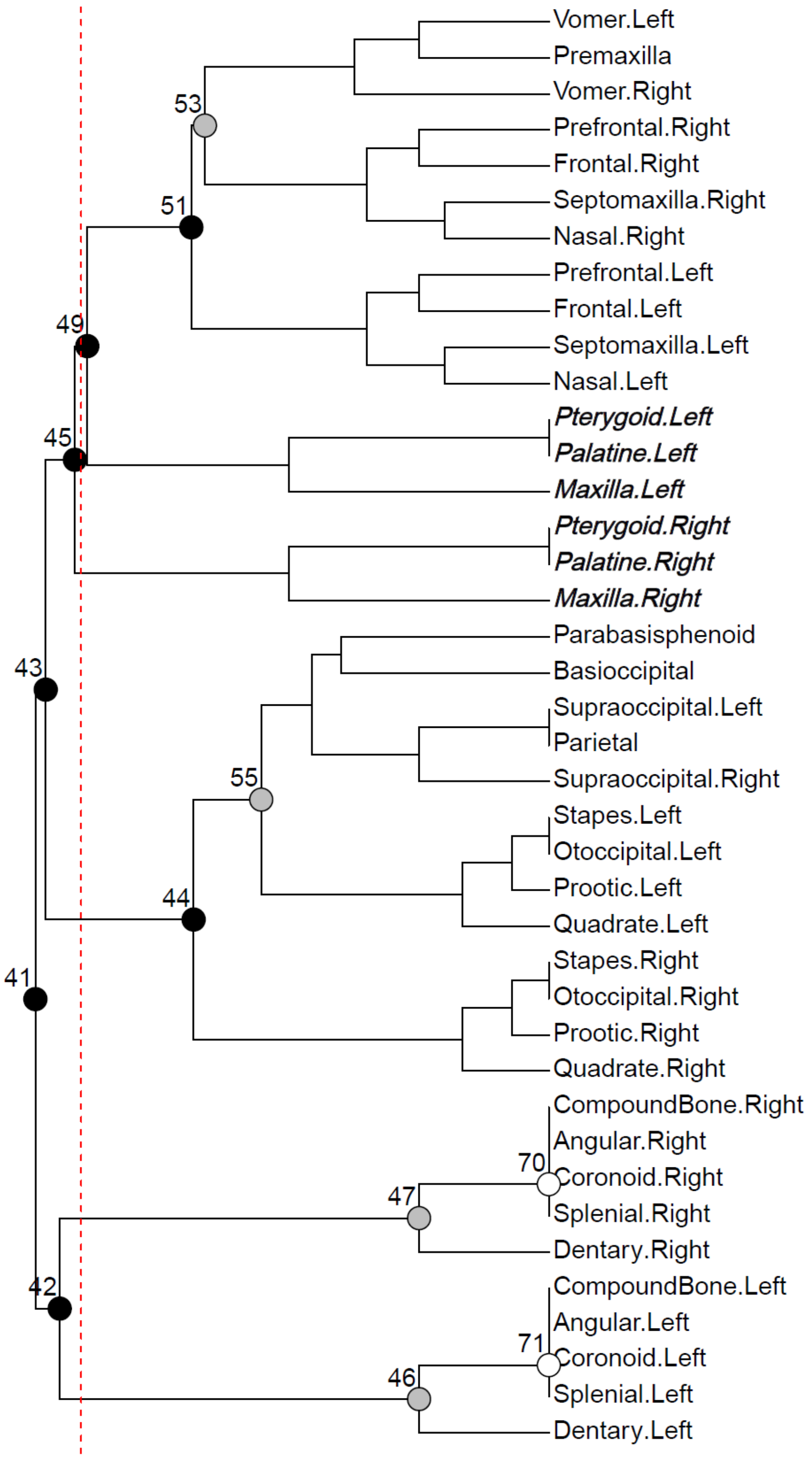

Supplementary Figure S3. Modularity of the skull network of *Afrotyphlops angolensis* (MCZ R-170385).

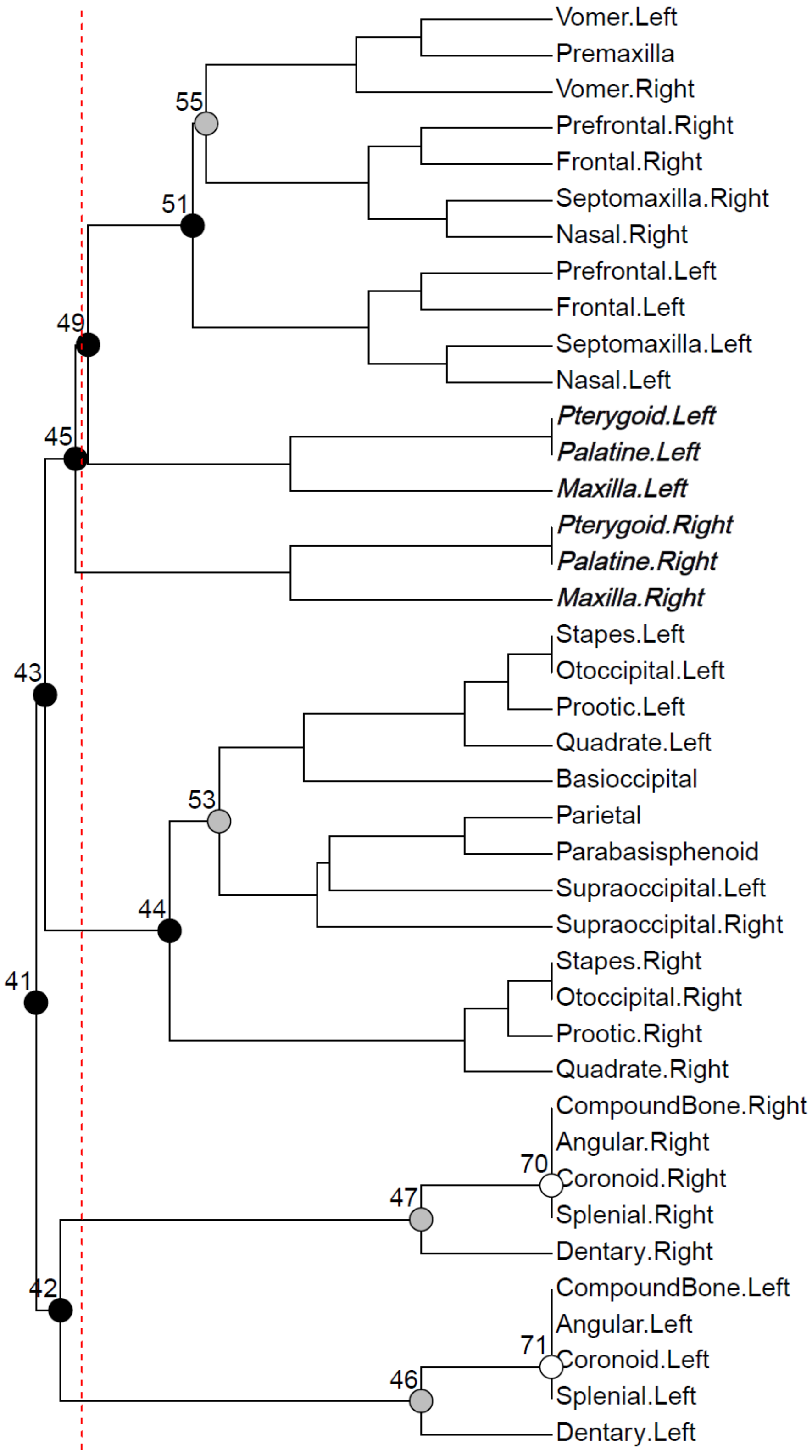

Supplementary Figure S4. Modularity of the skull network of *Amerotyphlops paucisquamus* (MCZ R-147336).

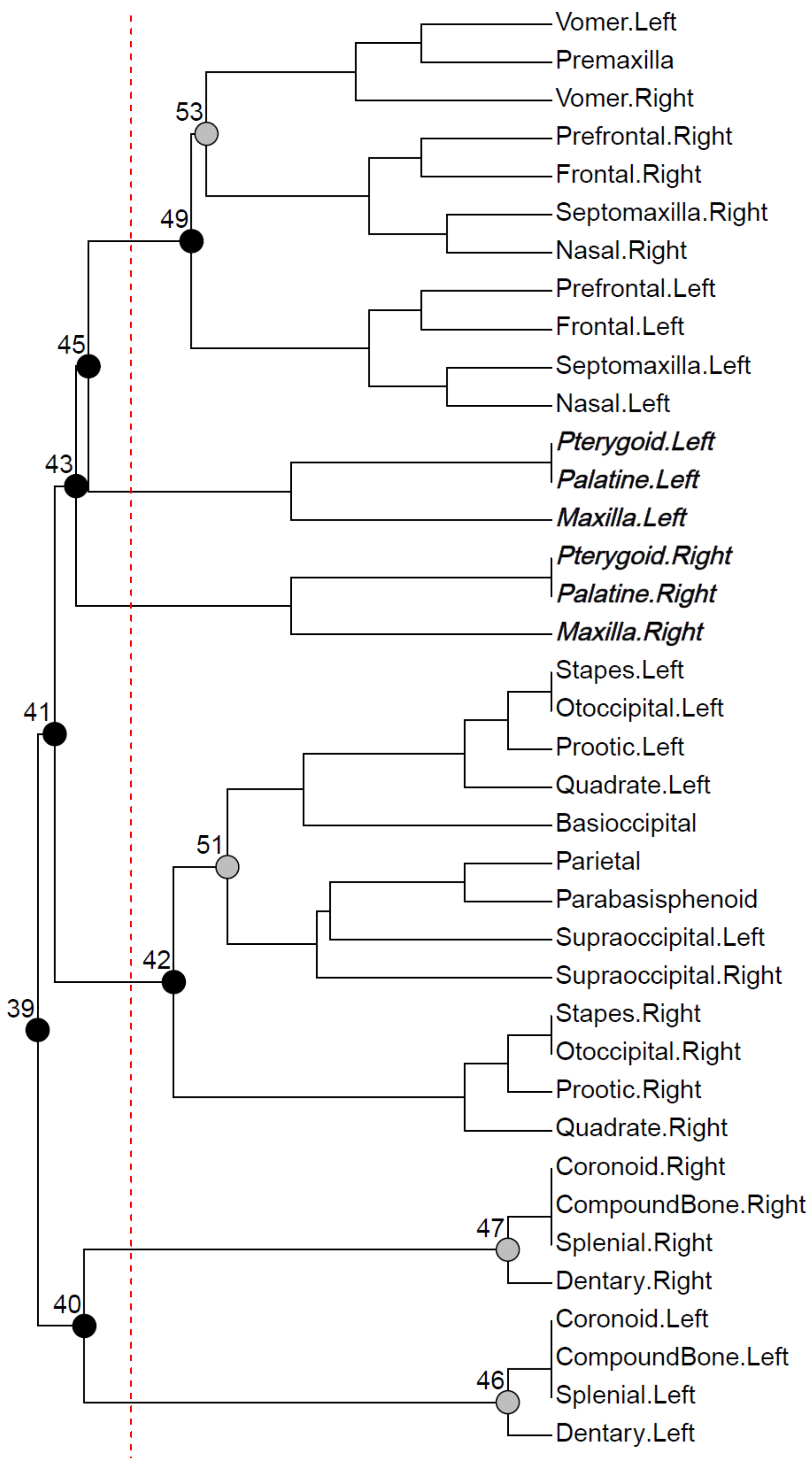

Supplementary Figure S5. Modularity of the skull network of *Anilios bicolor* (SAMA 60626).

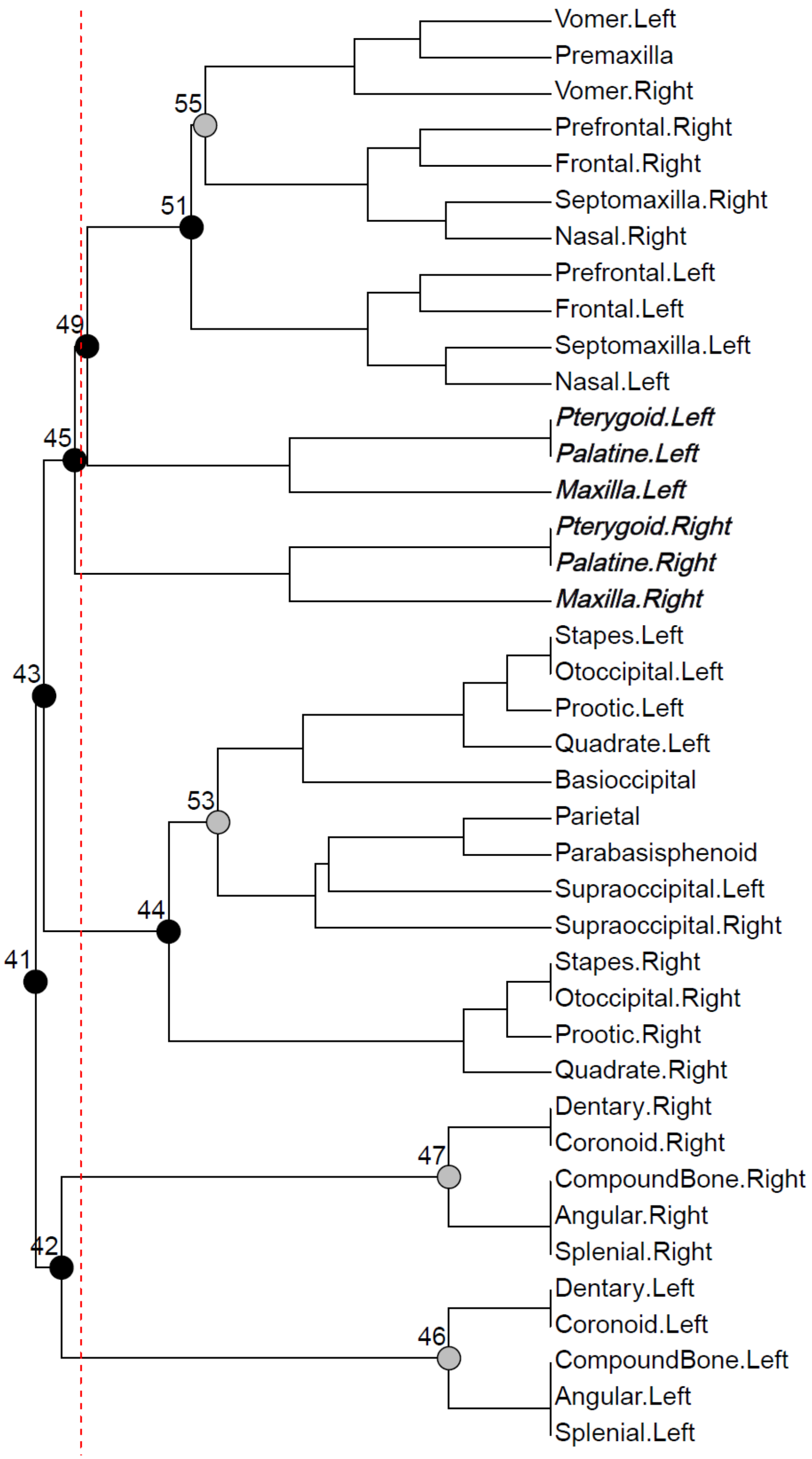

Supplementary Figure S6. Modularity of the skull network of *Antillotyphlops monastus* (MCZ R-81112).

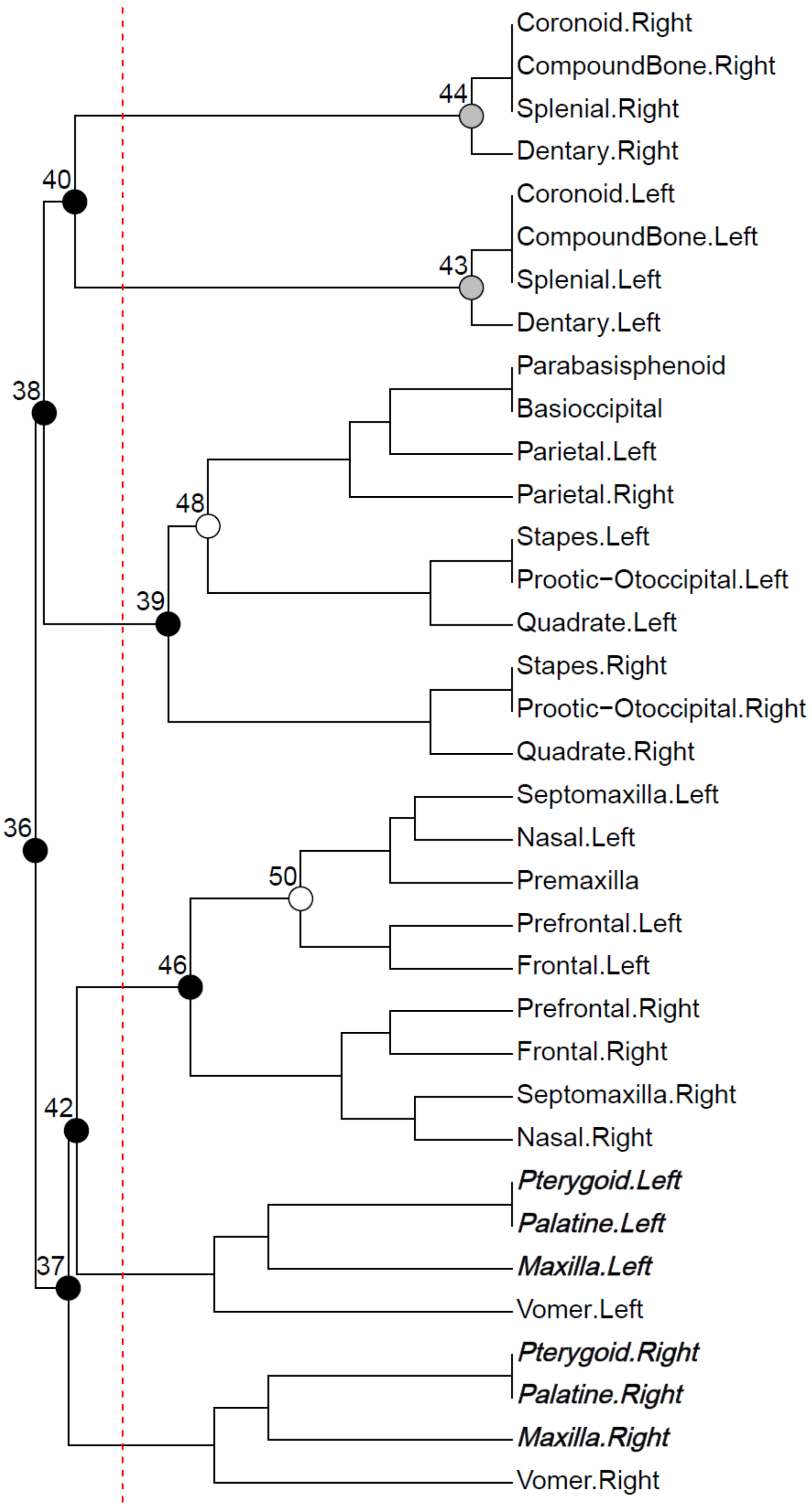

Supplementary Figure S7. Modularity of the skull network of *Gerrhopilus ater* (MCZ R-33505).

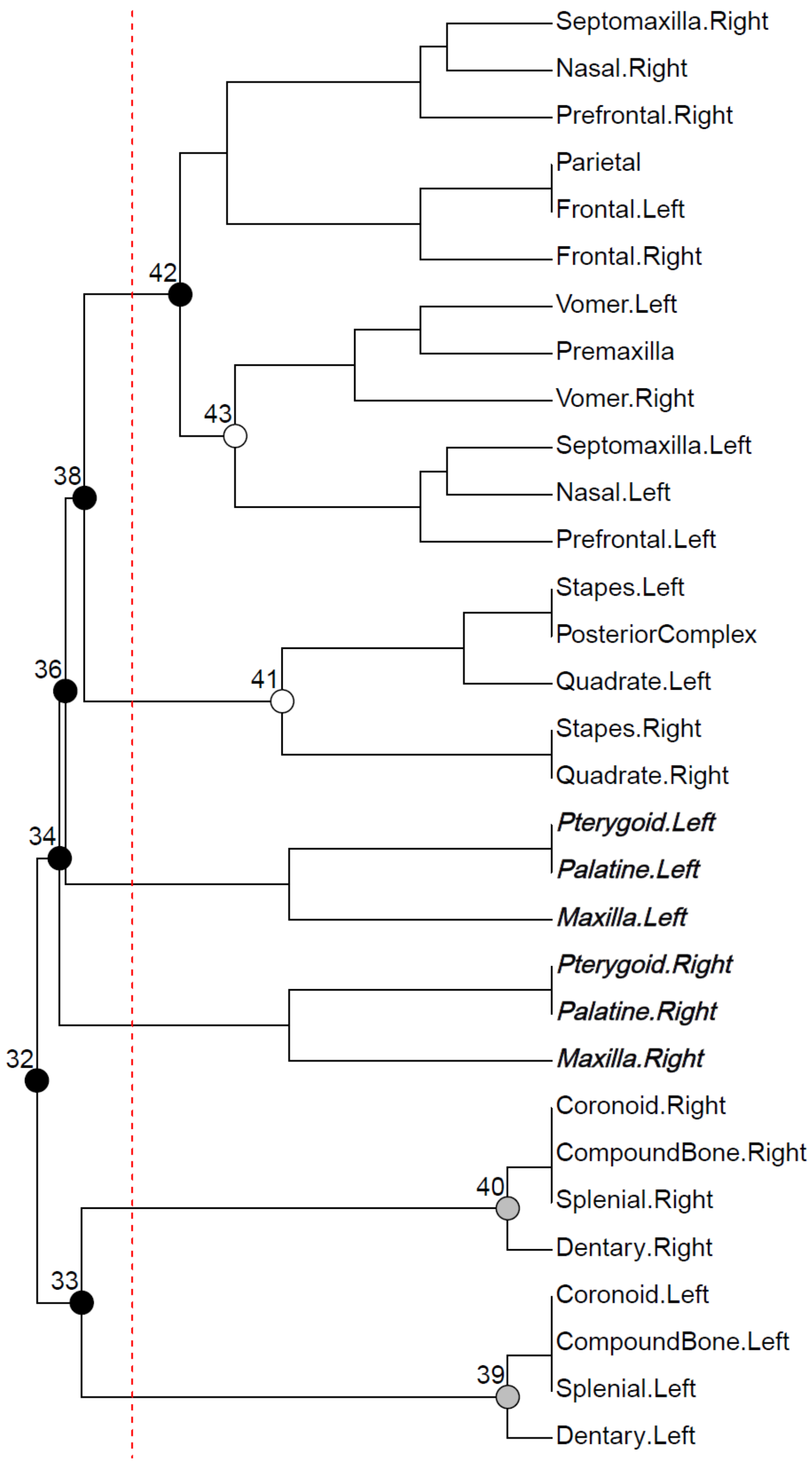

Supplementary Figure S8. Modularity of the skull network of *Gerrhopilus beddomii* (MCZ R-22372).

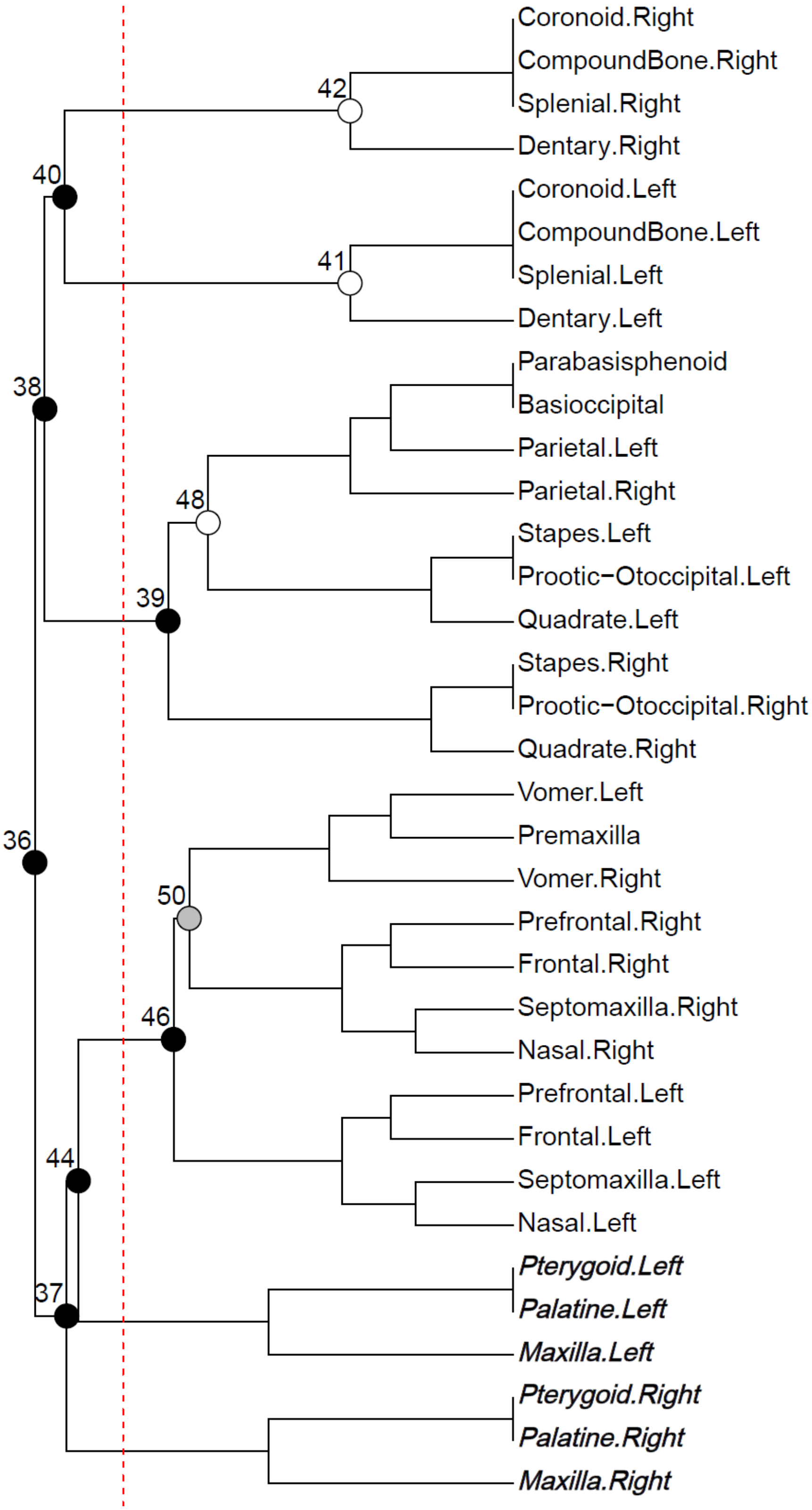

Supplementary Figure S9. Modularity of the skull network of *Indotyphlops braminus* (UAMZ R363).

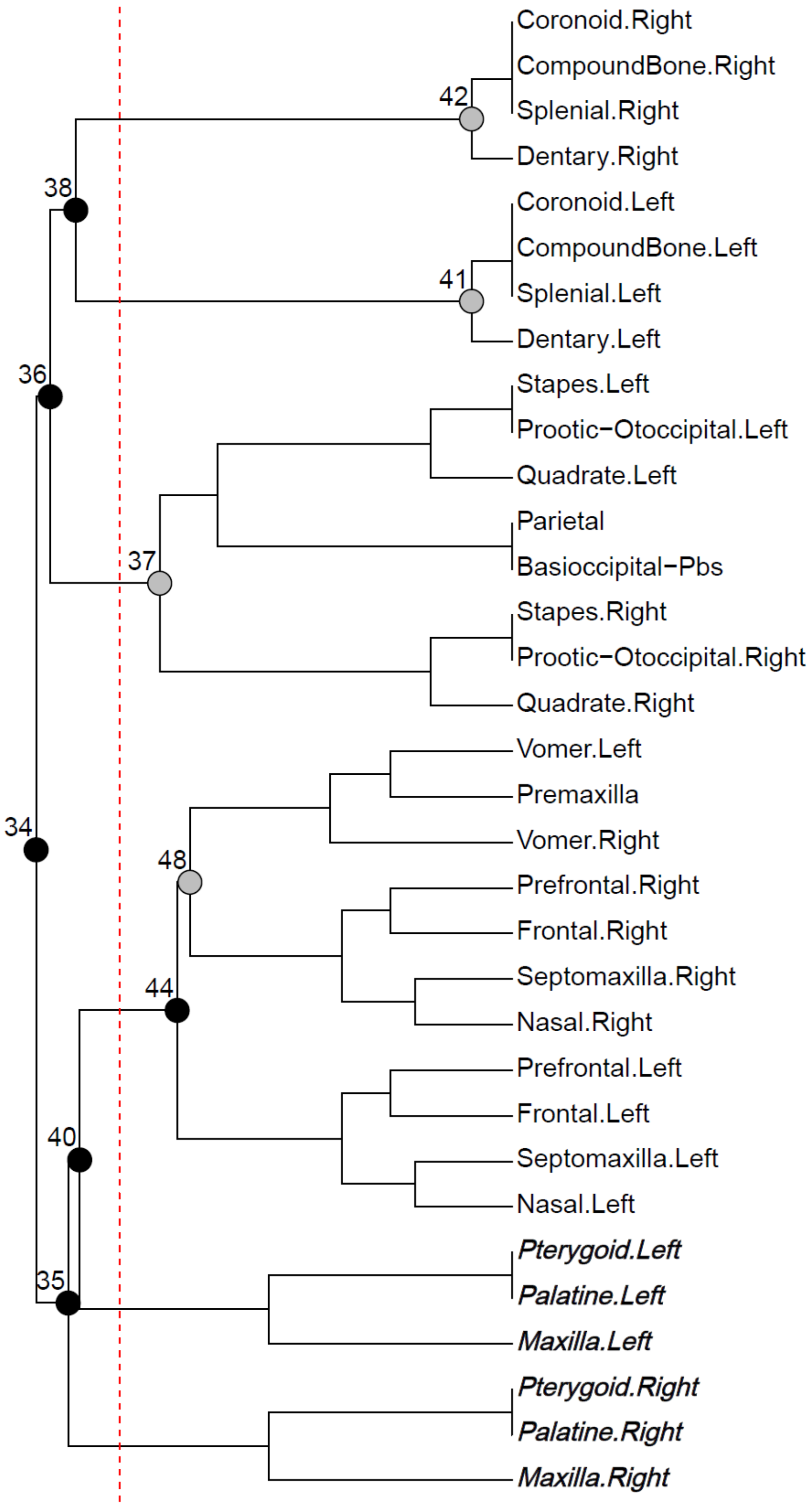

Supplementary Figure S10. Modularity of the skull network of *Ramphotyphlops lineatus* (MCZ R-37751).

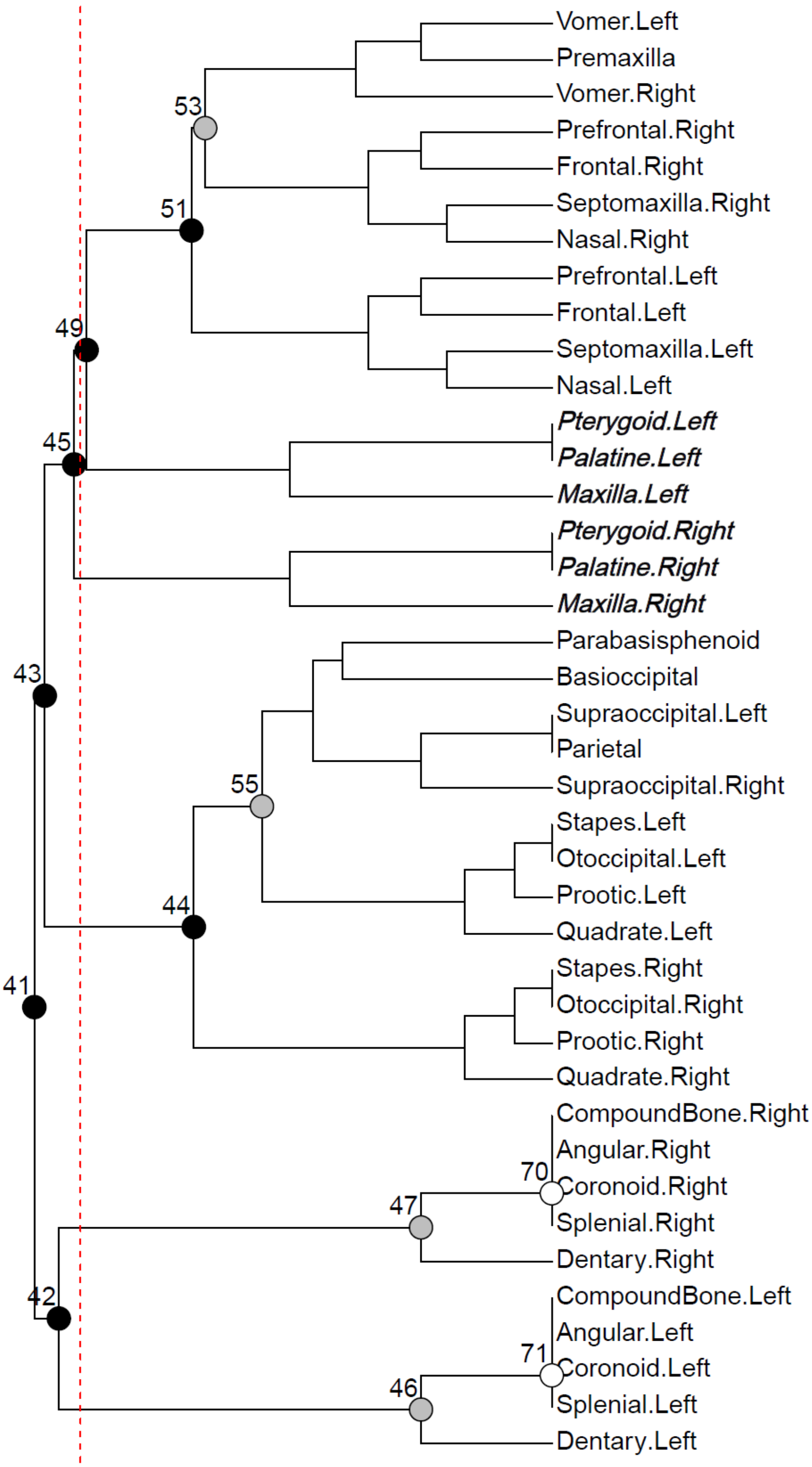

Supplementary Figure S11. Modularity of the skull network of *Typhlops titanops* (MCZ R-68571).

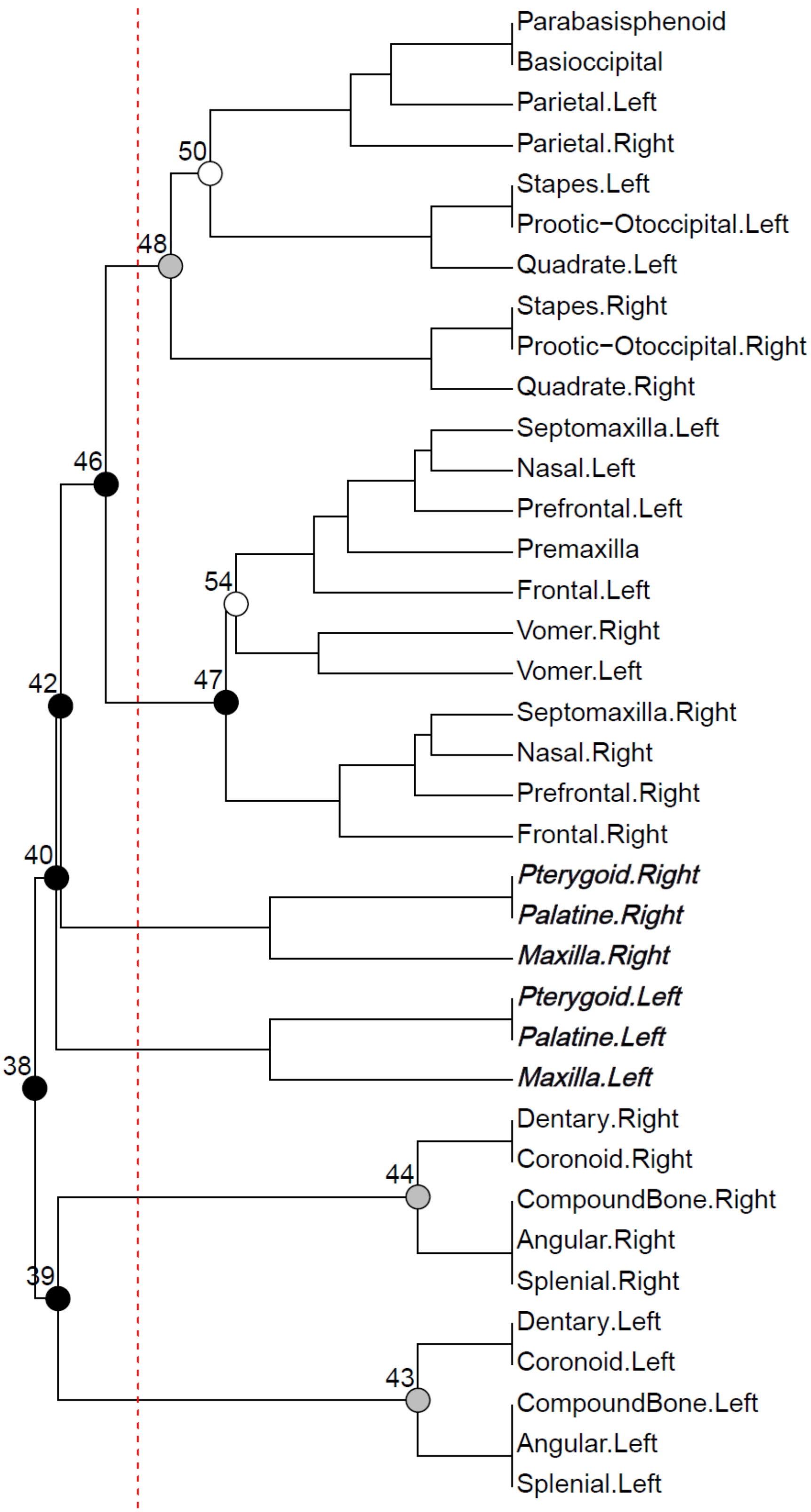

Supplementary Figure S12. Modularity of the skull network of *Xenotyphlops grandidieri* (ZSM 2194/2007).

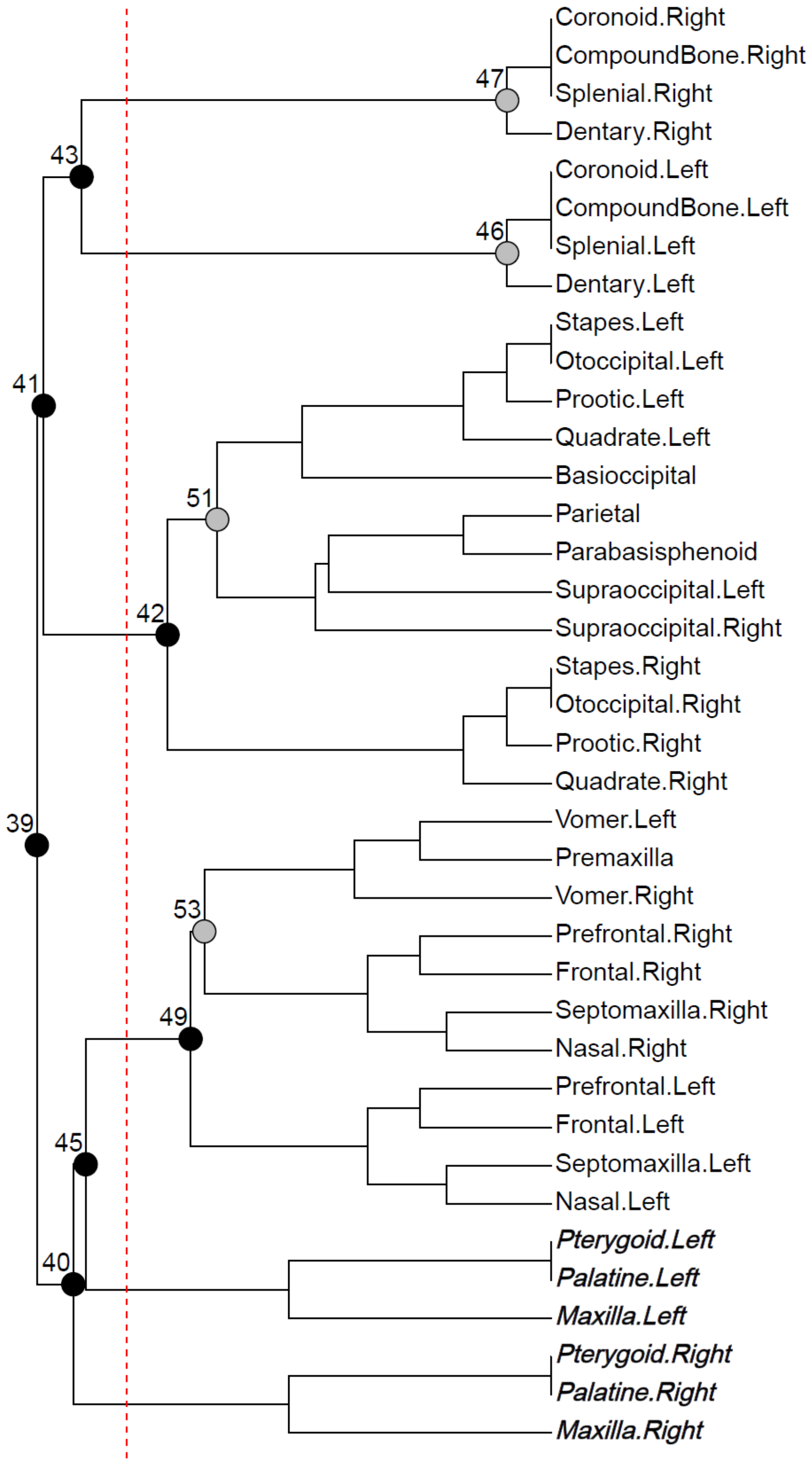

Supplementary Figure S13. Modularity of the skull network of *Xerotyphlops vermicularis* (MCZ R-56477).

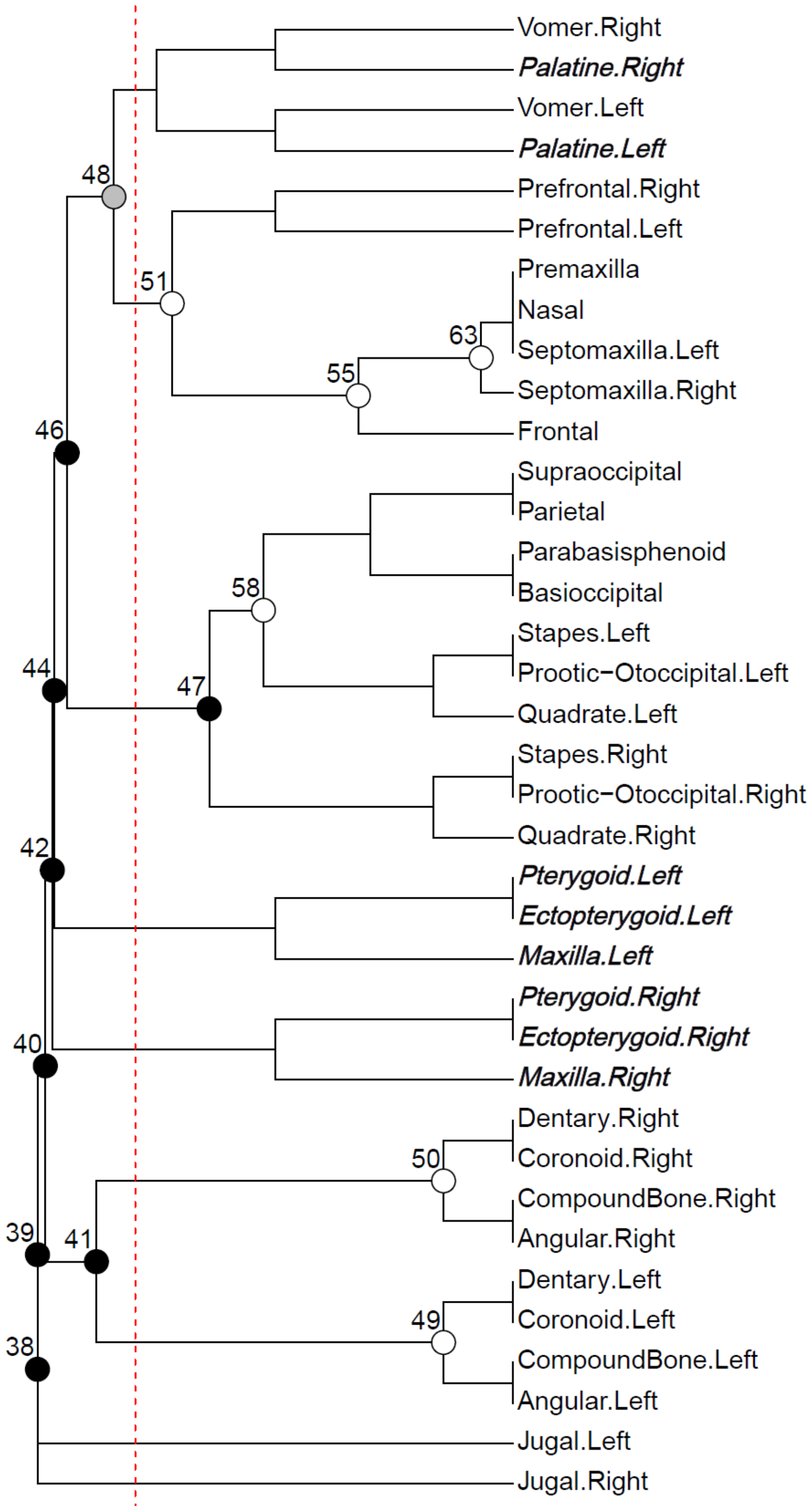

Supplementary Figure S14. Modularity of the skull network of *Anomalepis mexicanus* (MCZ R-191201).

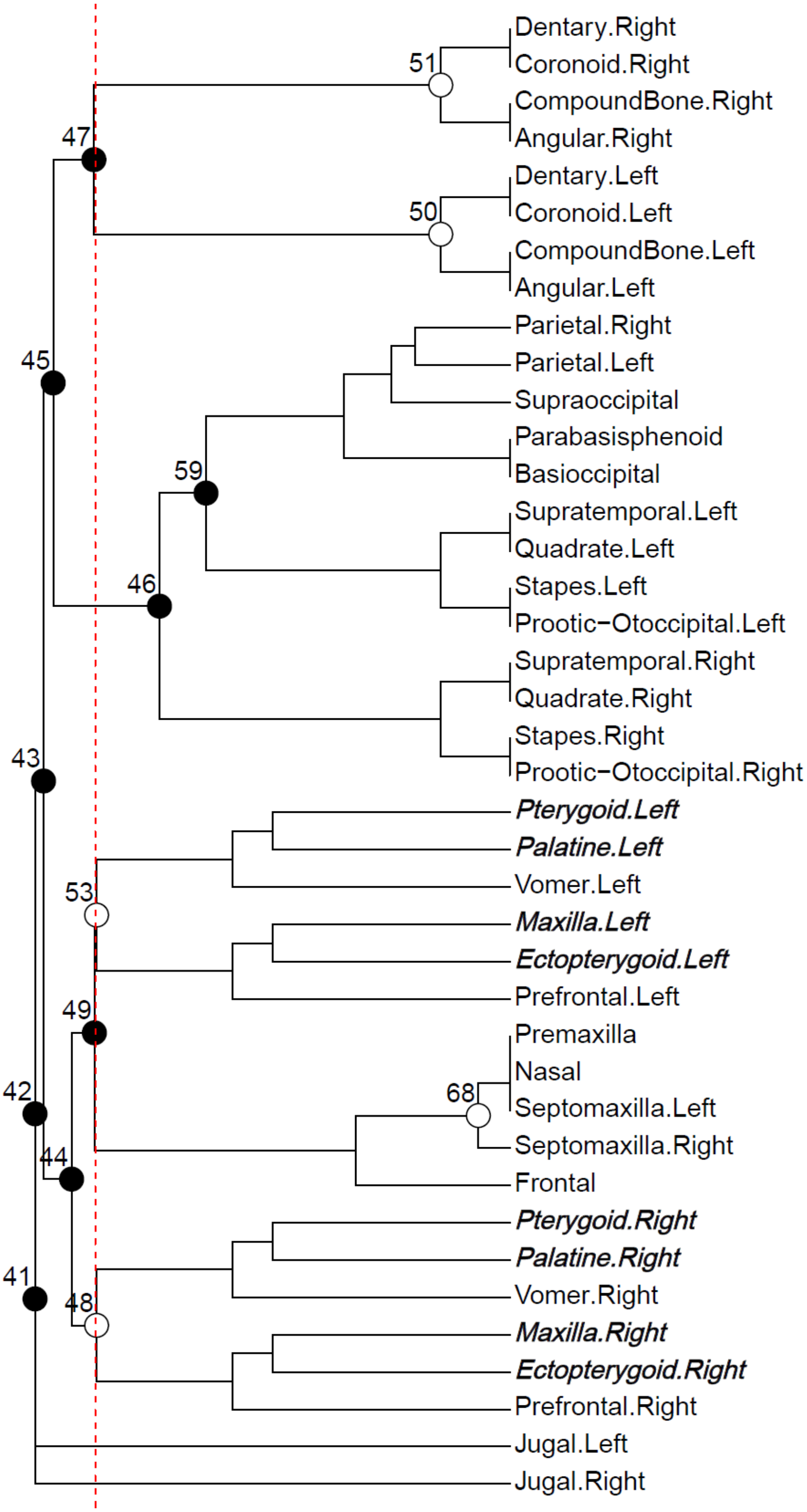

Supplementary Figure S15. Modularity of the skull network of *Helminthophis praeocularis* (MCZ R-17960).

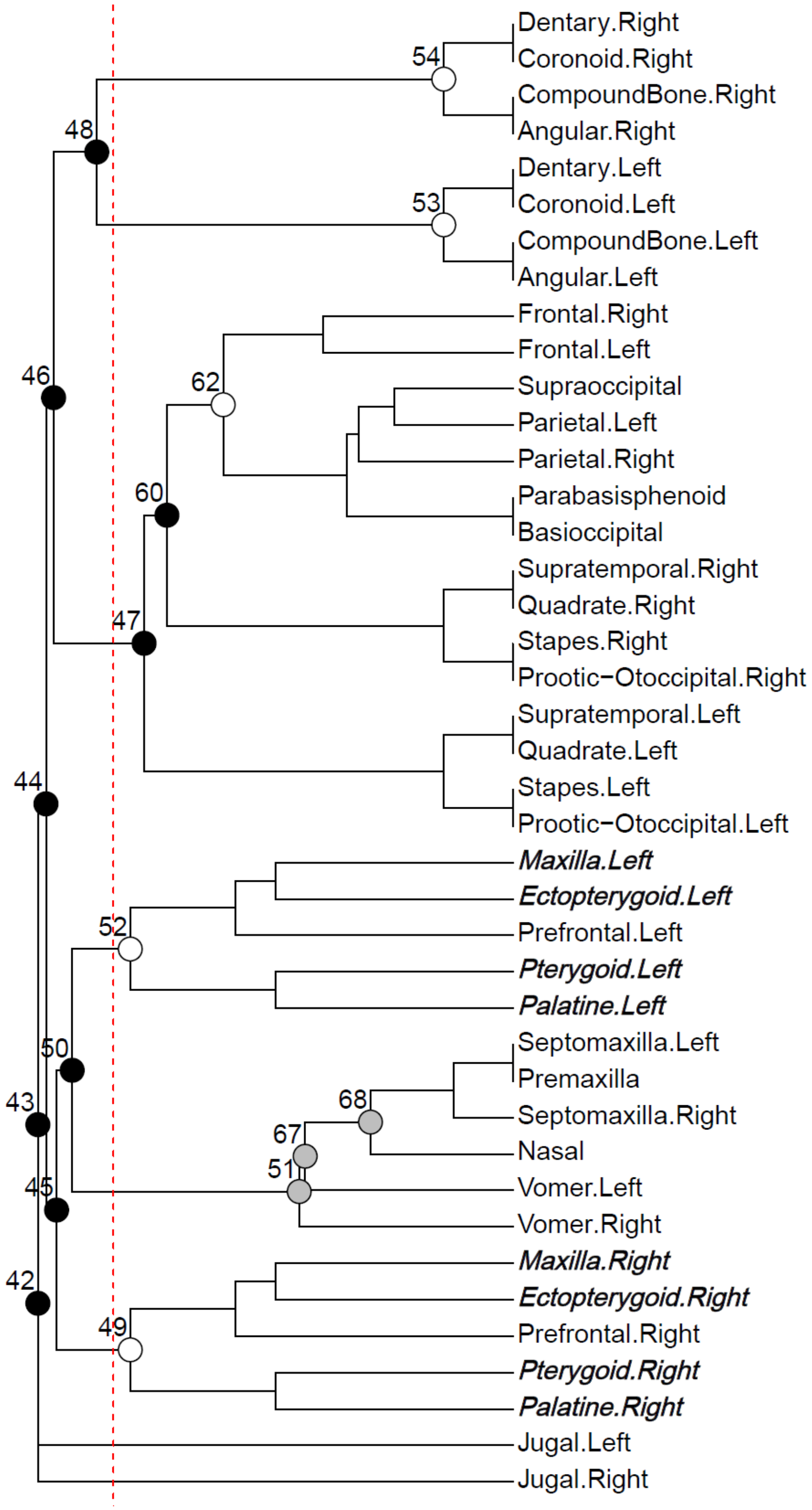

Supplementary Figure S16. Modularity of the skull network of *Liotyphlops albirostris* (FMNH 216257, KUH 116885).

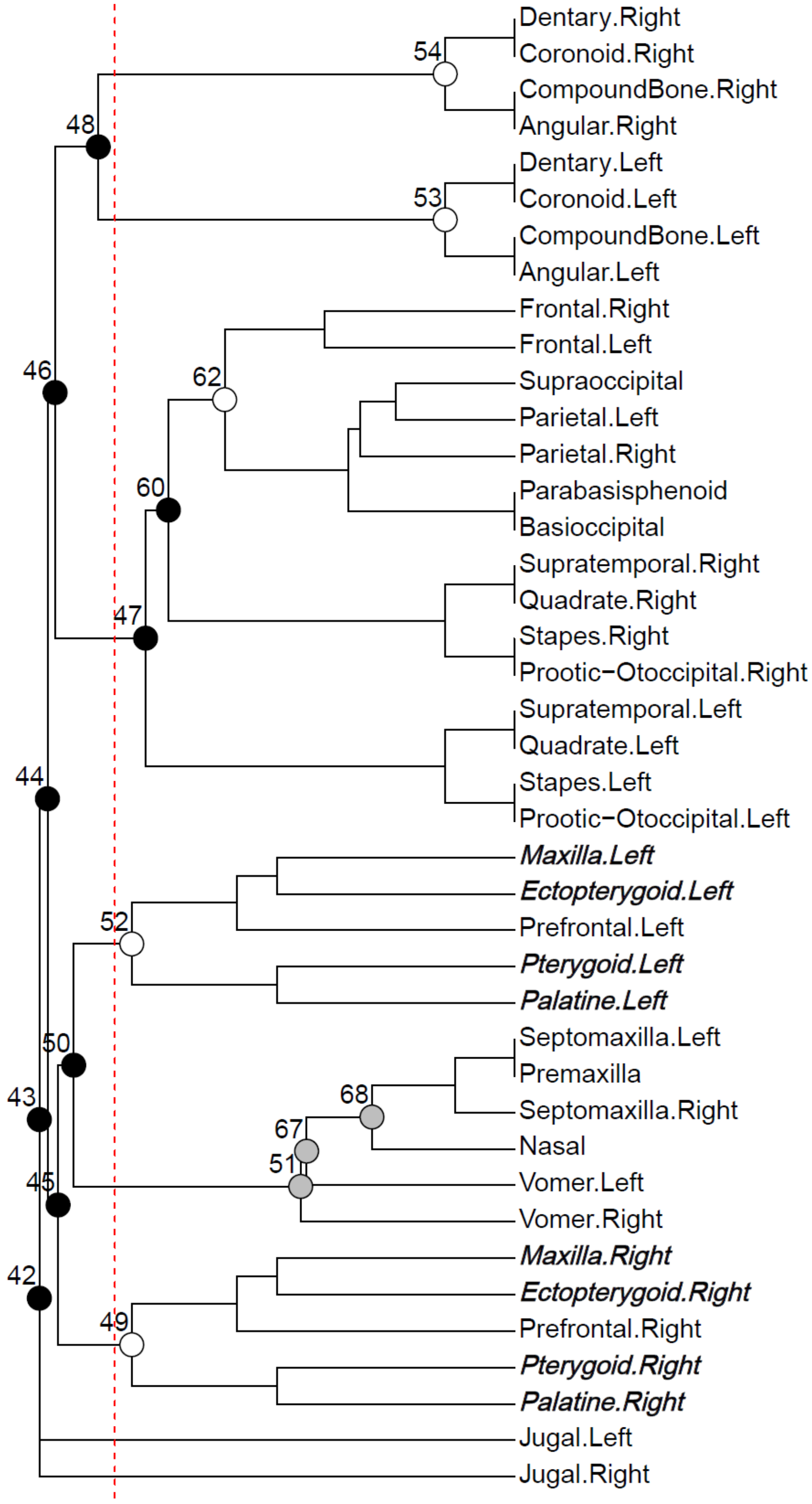

Supplementary Figure S17. Modularity of the skull network of *Liotyphlops argaleus* (MCZ R-67933).

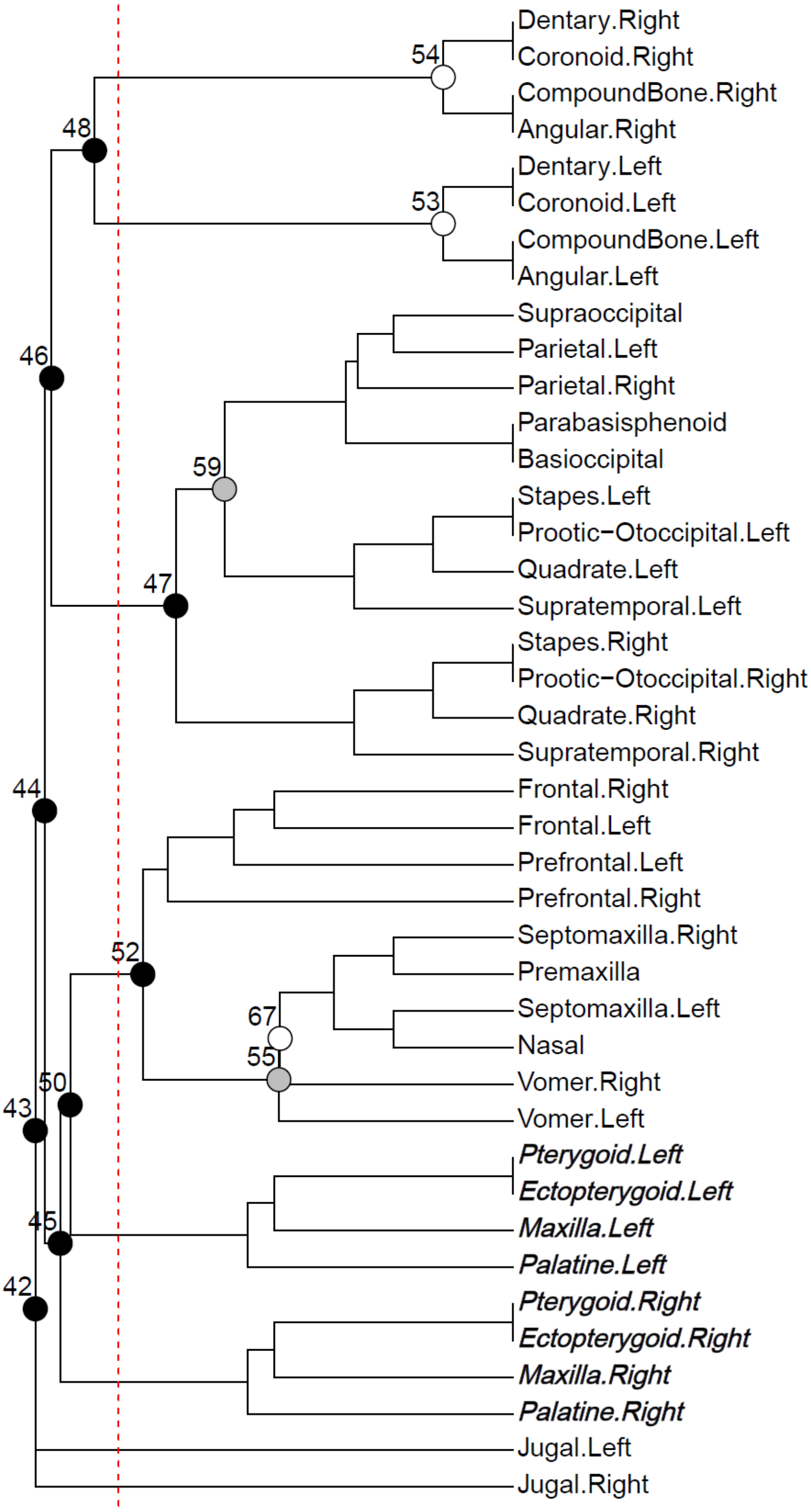

Supplementary Figure S18. Modularity of the skull network of *Liotyphlops beui* (SAMA 40142).

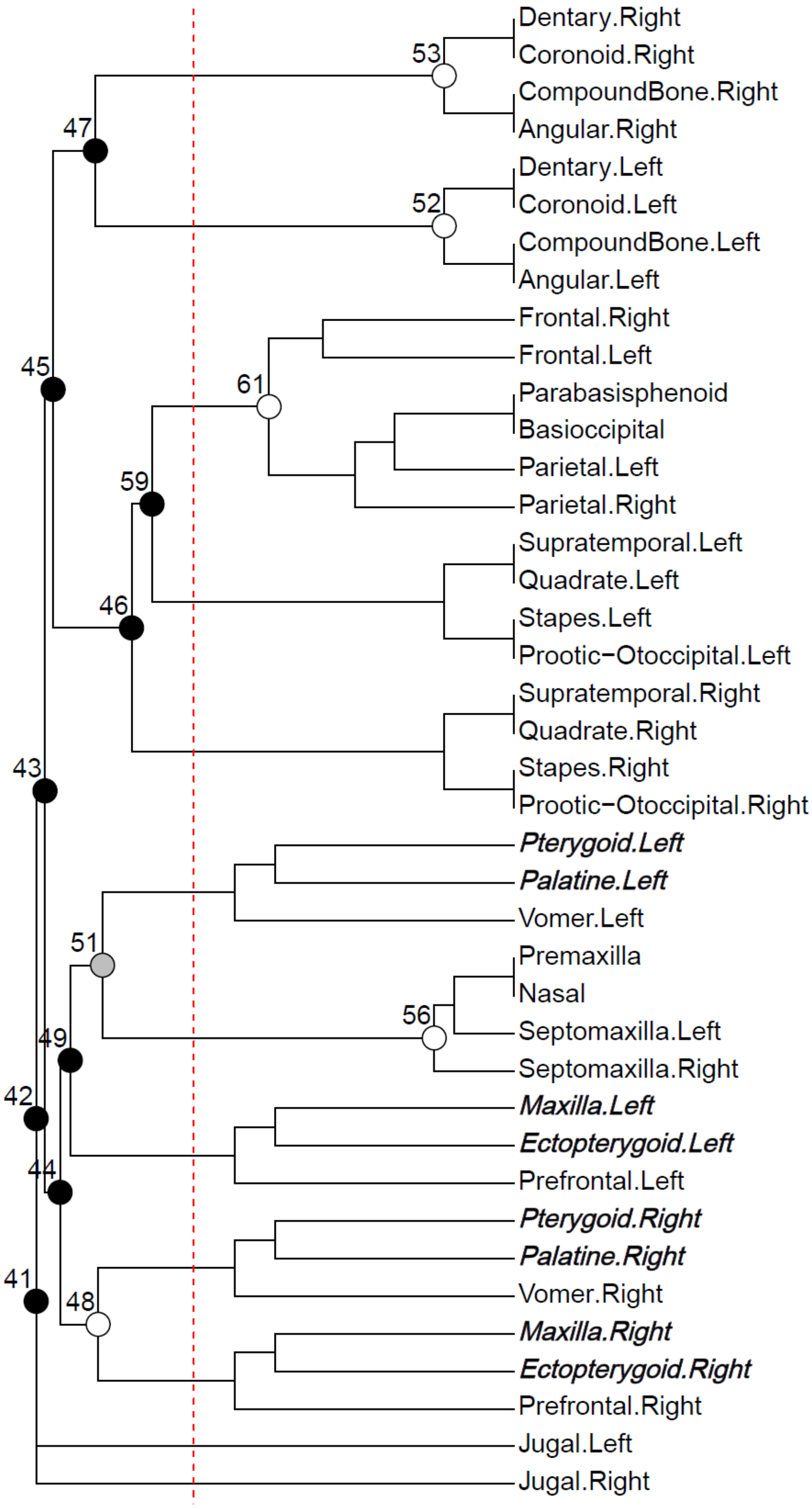

Supplementary Figure S19. Modularity of the skull network of *Typhlophis squamosus* (MCZ R-145403).

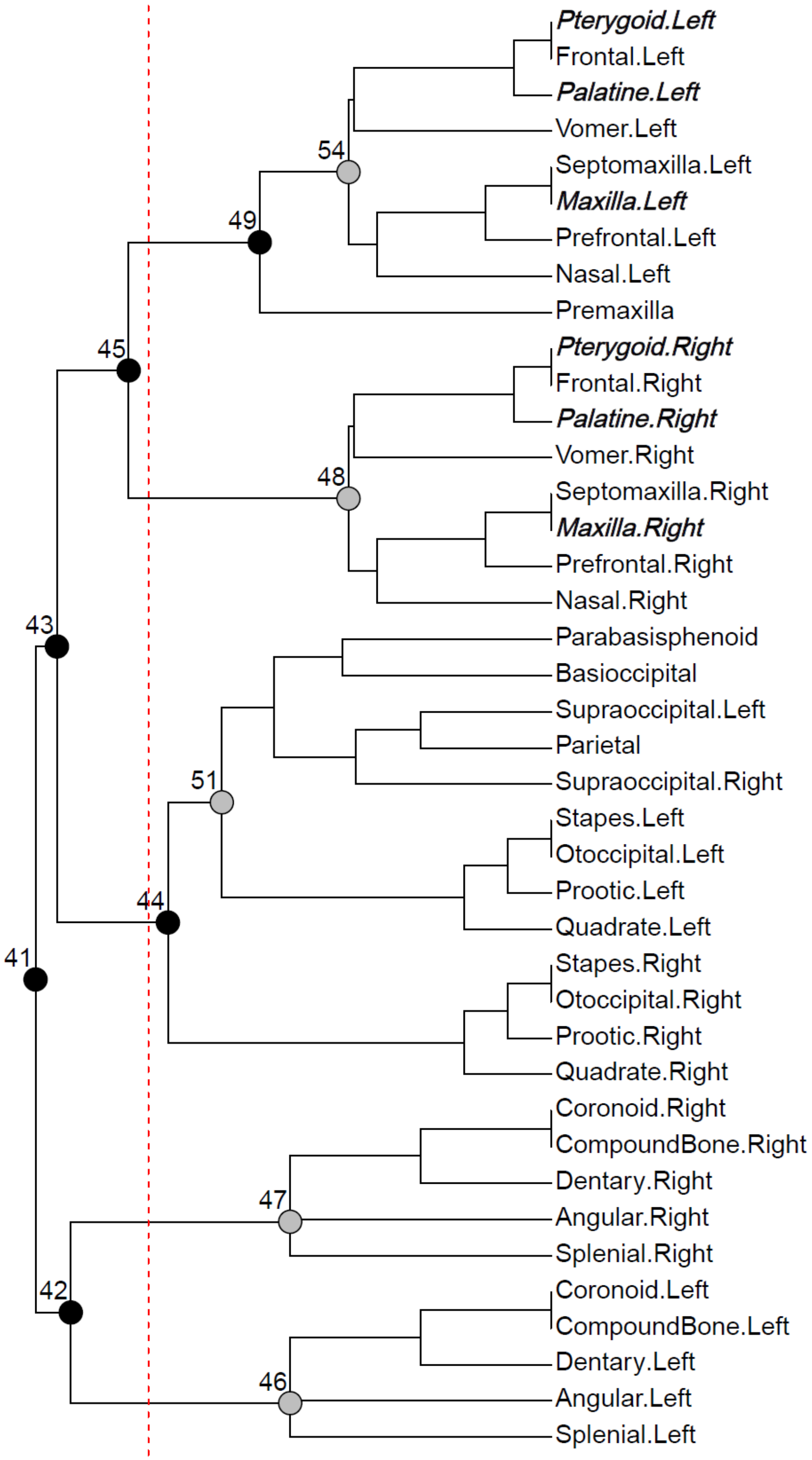

Supplementary Figure S20. Modularity of the skull network of *Epictia albifrons* (MCZ R-2885).

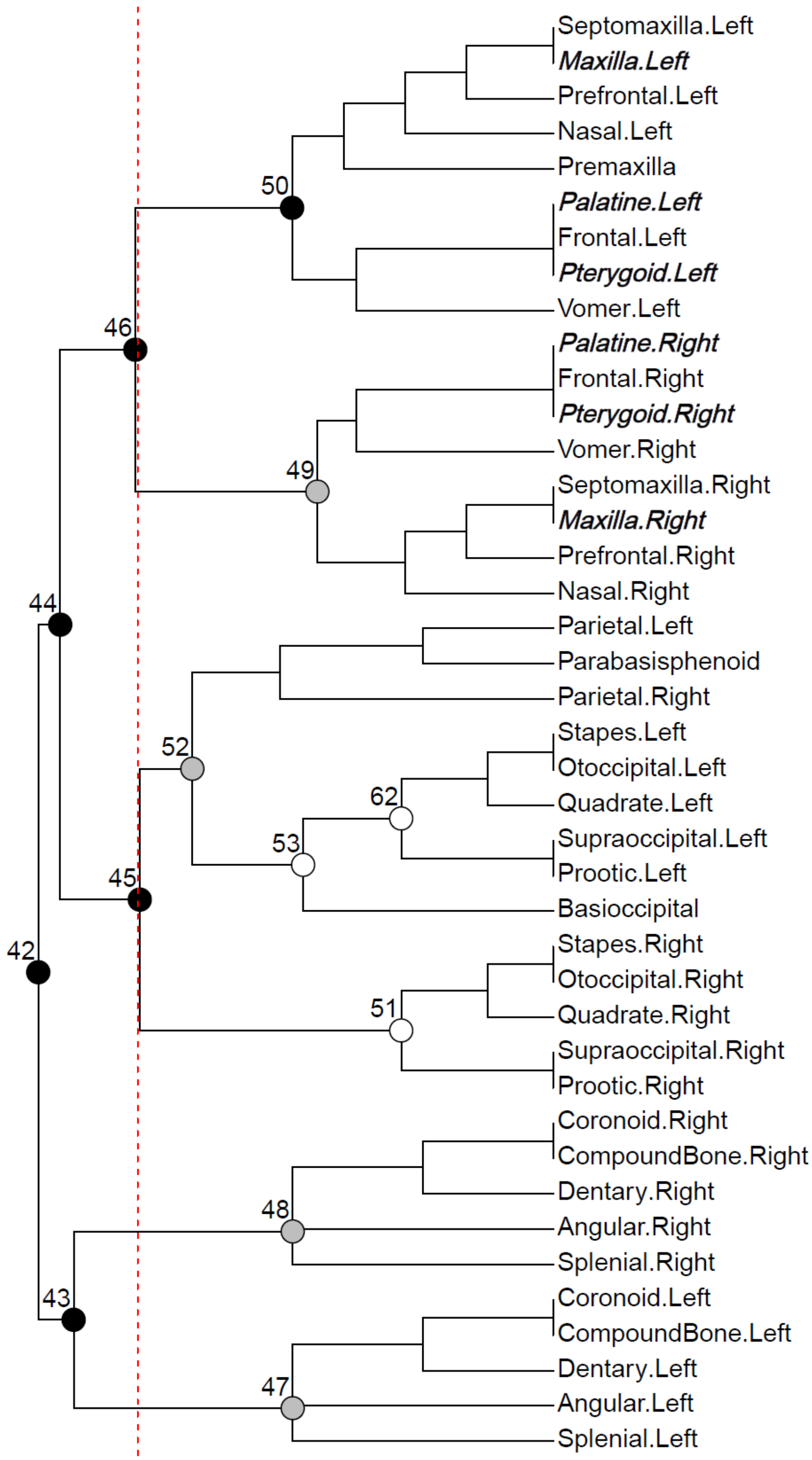

Supplementary Figure S21. Modularity of the skull network of *Myriopholis macrorhyncha* (MCZ R-9650).

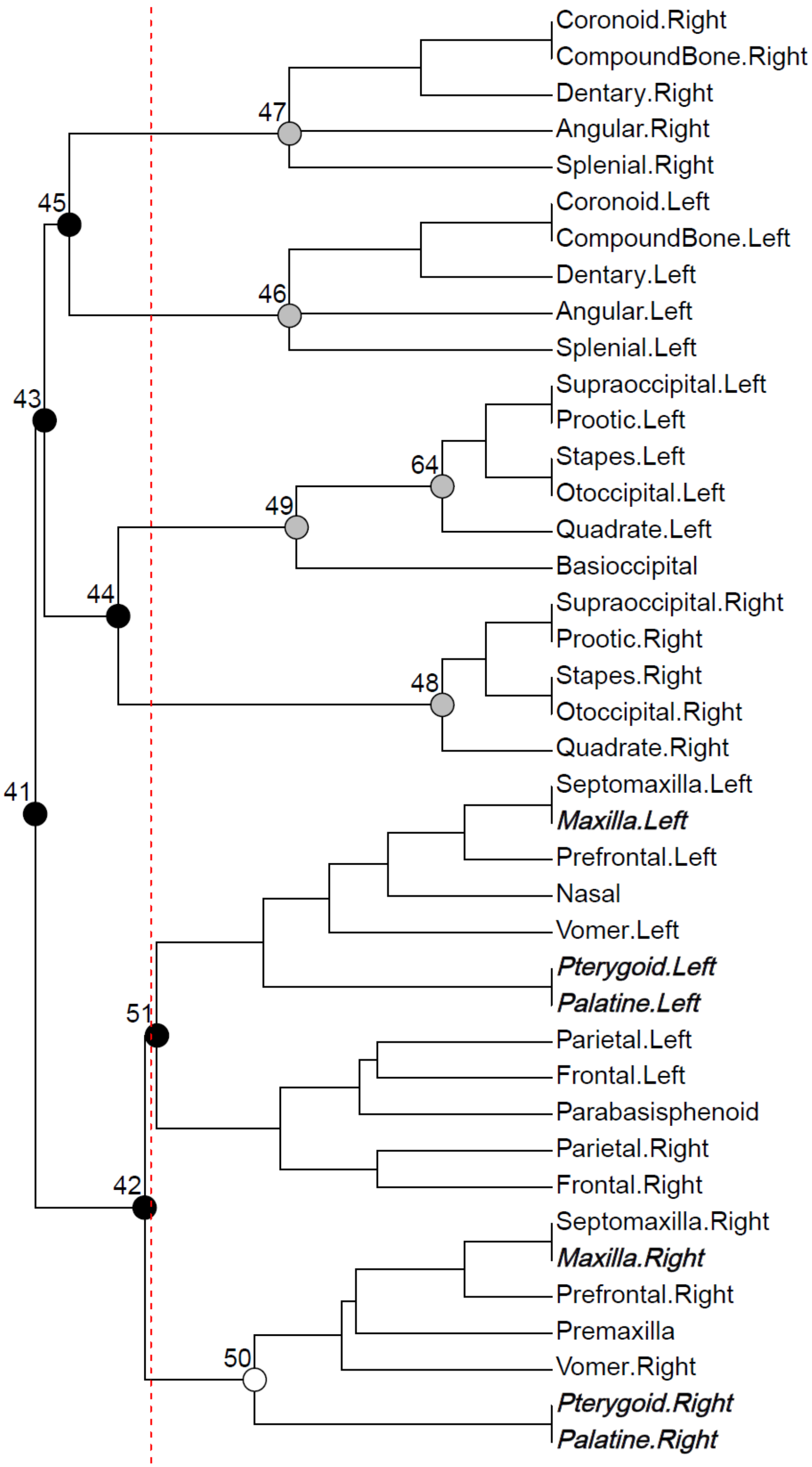

Supplementary Figure S22. Modularity of the skull network of *Myriopholis tanae* (MCZ R-40099).

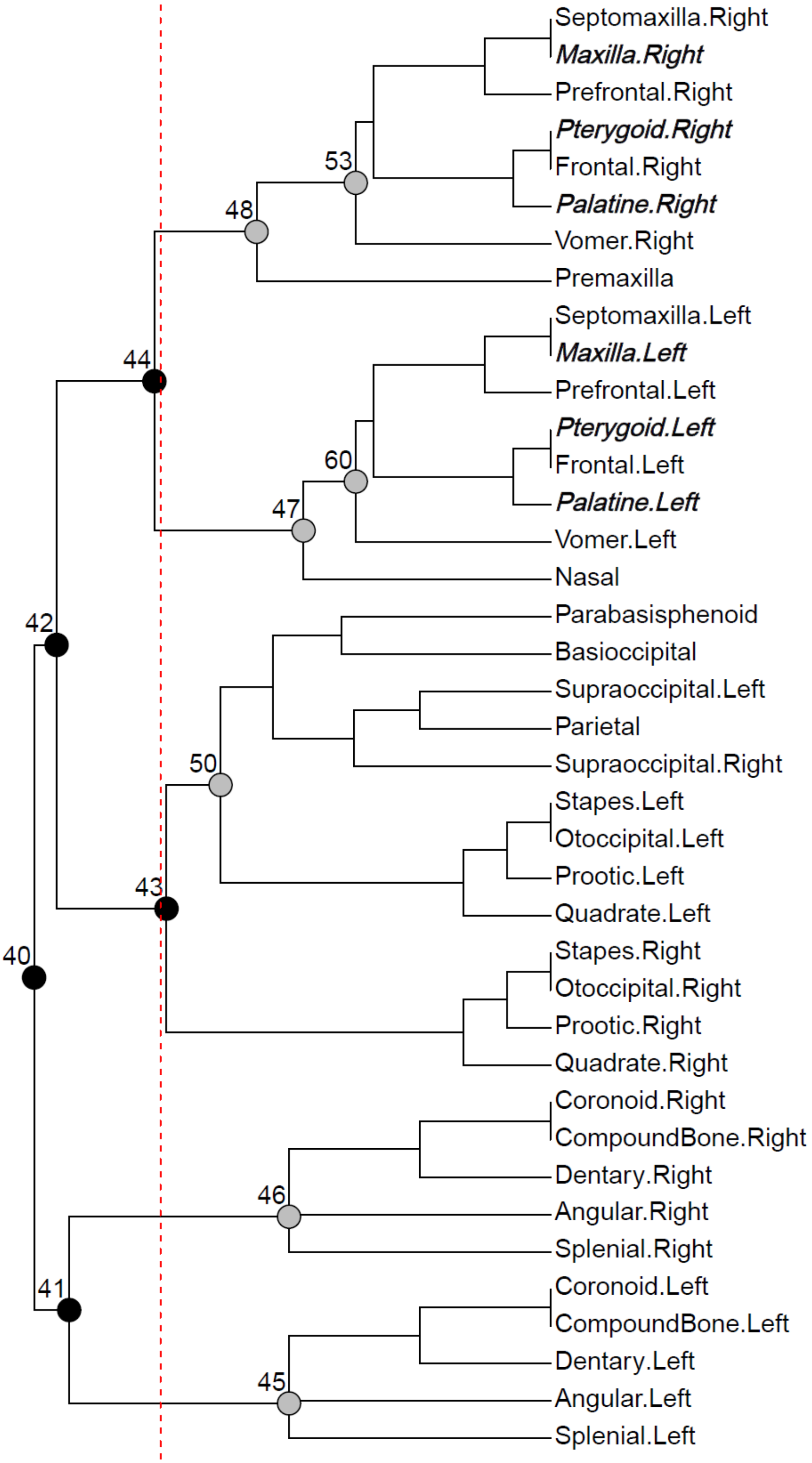

Supplementary Figure S23. Modularity of the skull network of *Rena dulcis* (UAMZ R335).

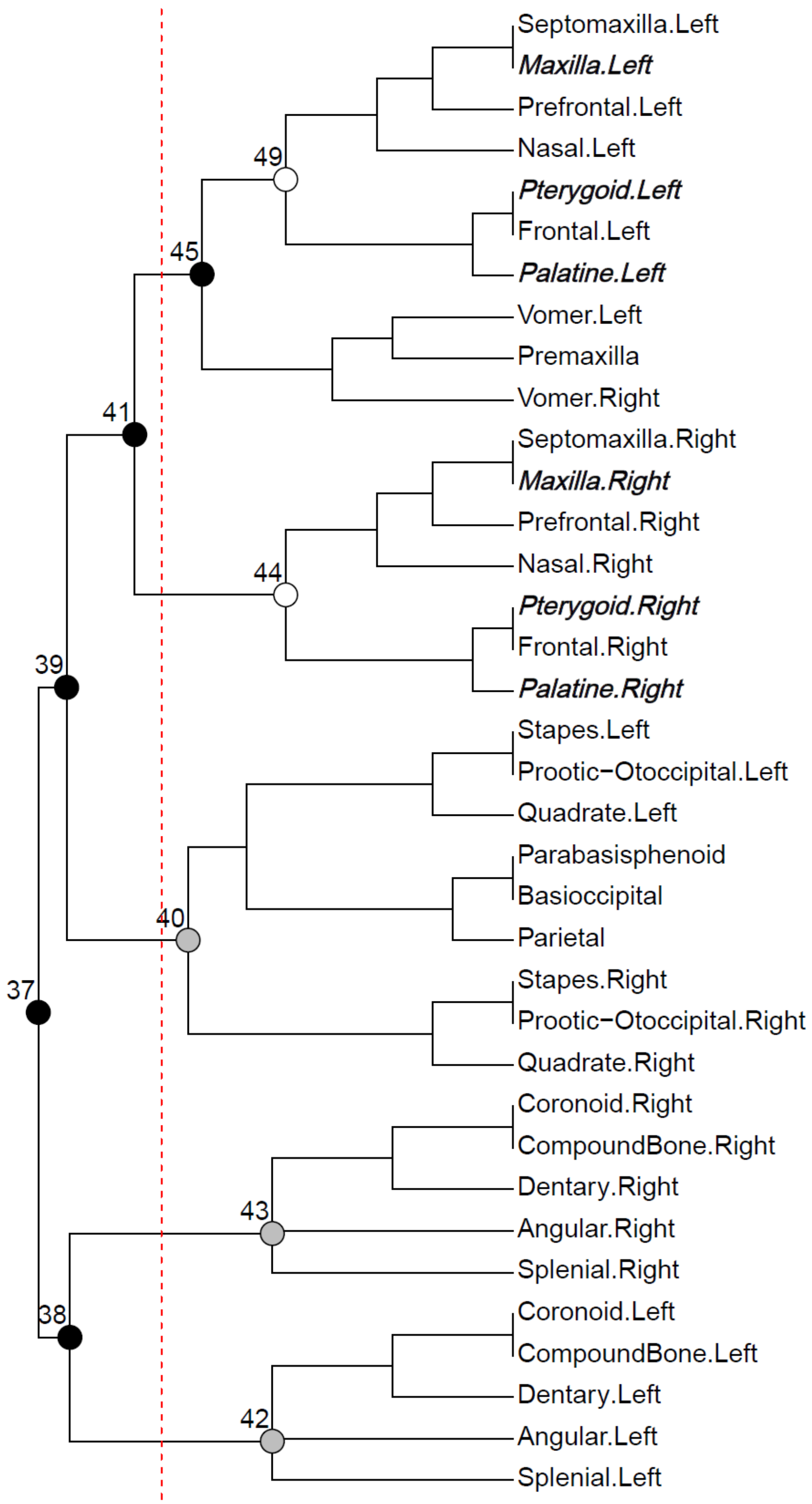

Supplementary Figure S24. Modularity of the skull network of *Tricheilostoma bicolor* (MCZ R-49718).

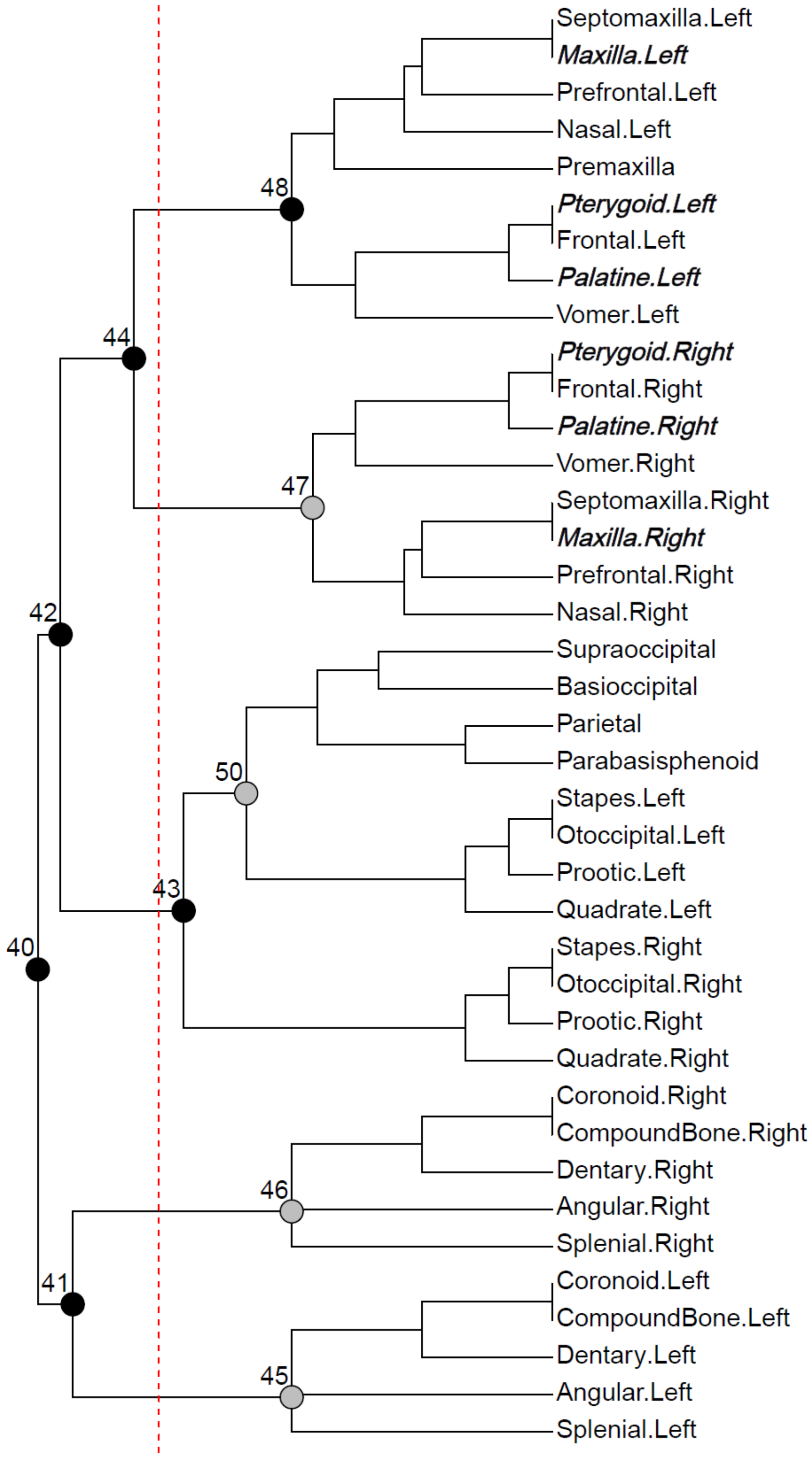

Supplementary Figure S25. Modularity of the skull network of *Trilepida dimidiata* (SAMA 40143).

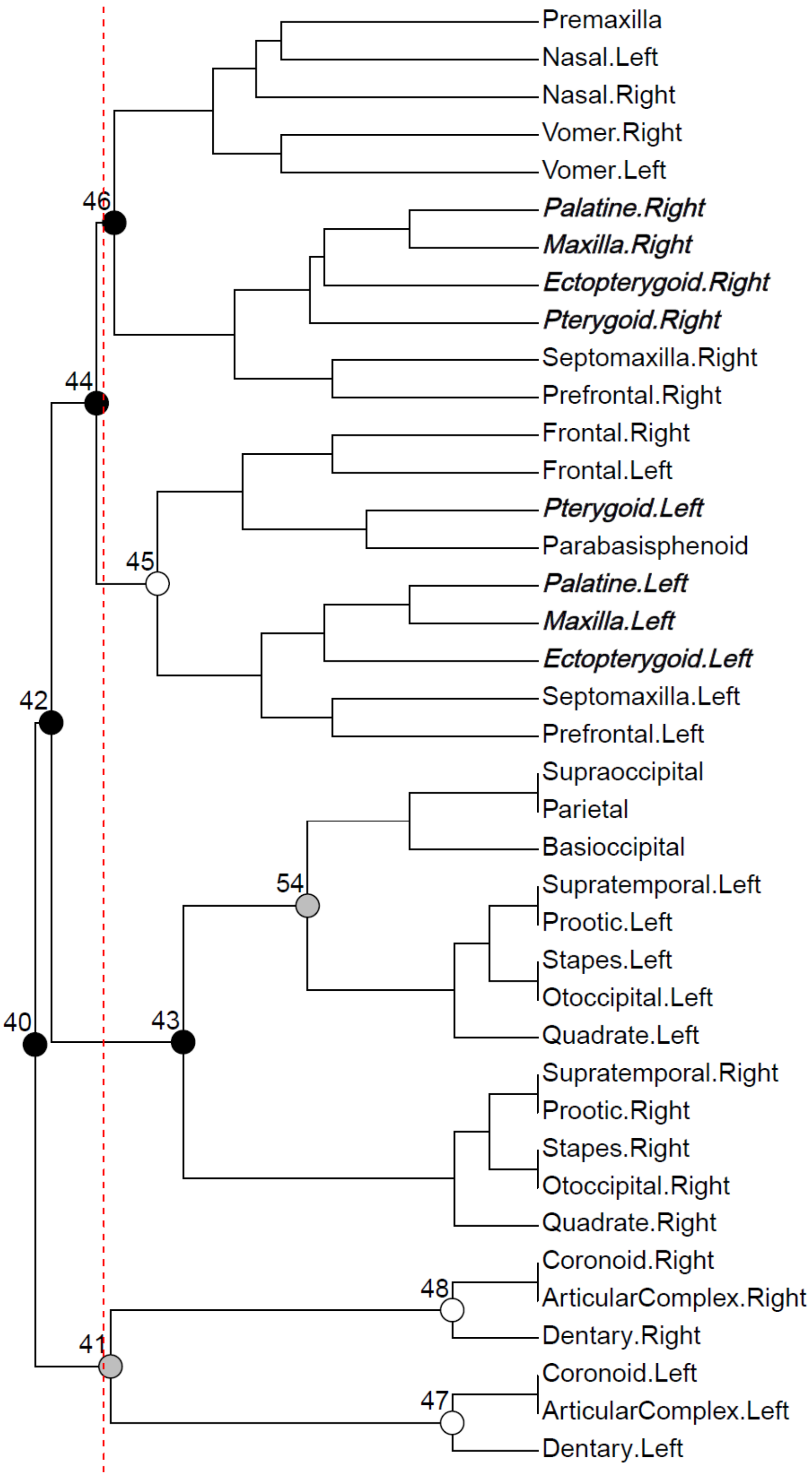

Supplementary Figure S26. Modularity of the skull network of *Anilius scytale* (KUH 125976).

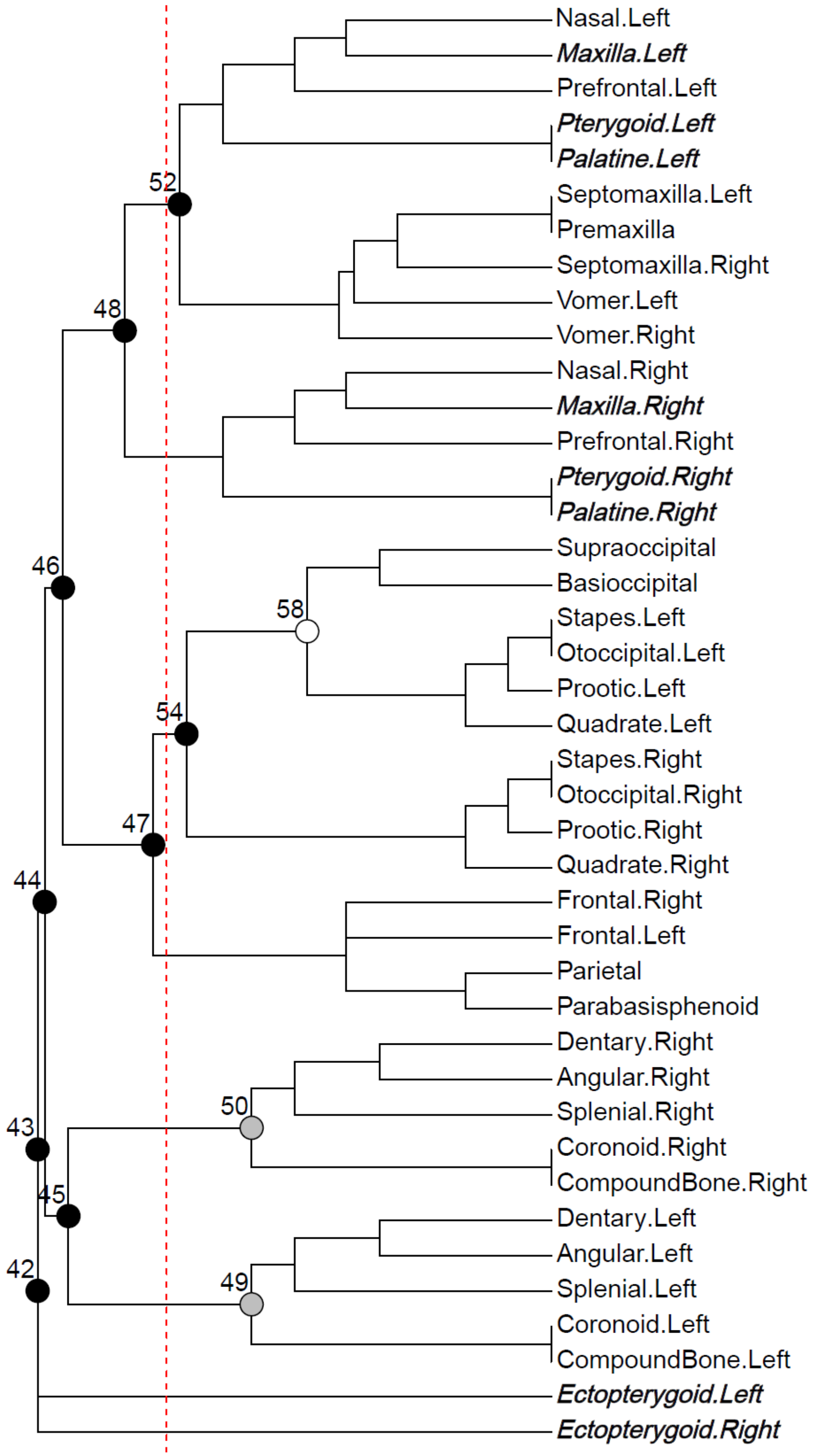

Supplementary Figure S27. Modularity of the skull network of *Anomochilus leonardi* (FRIM 0026).

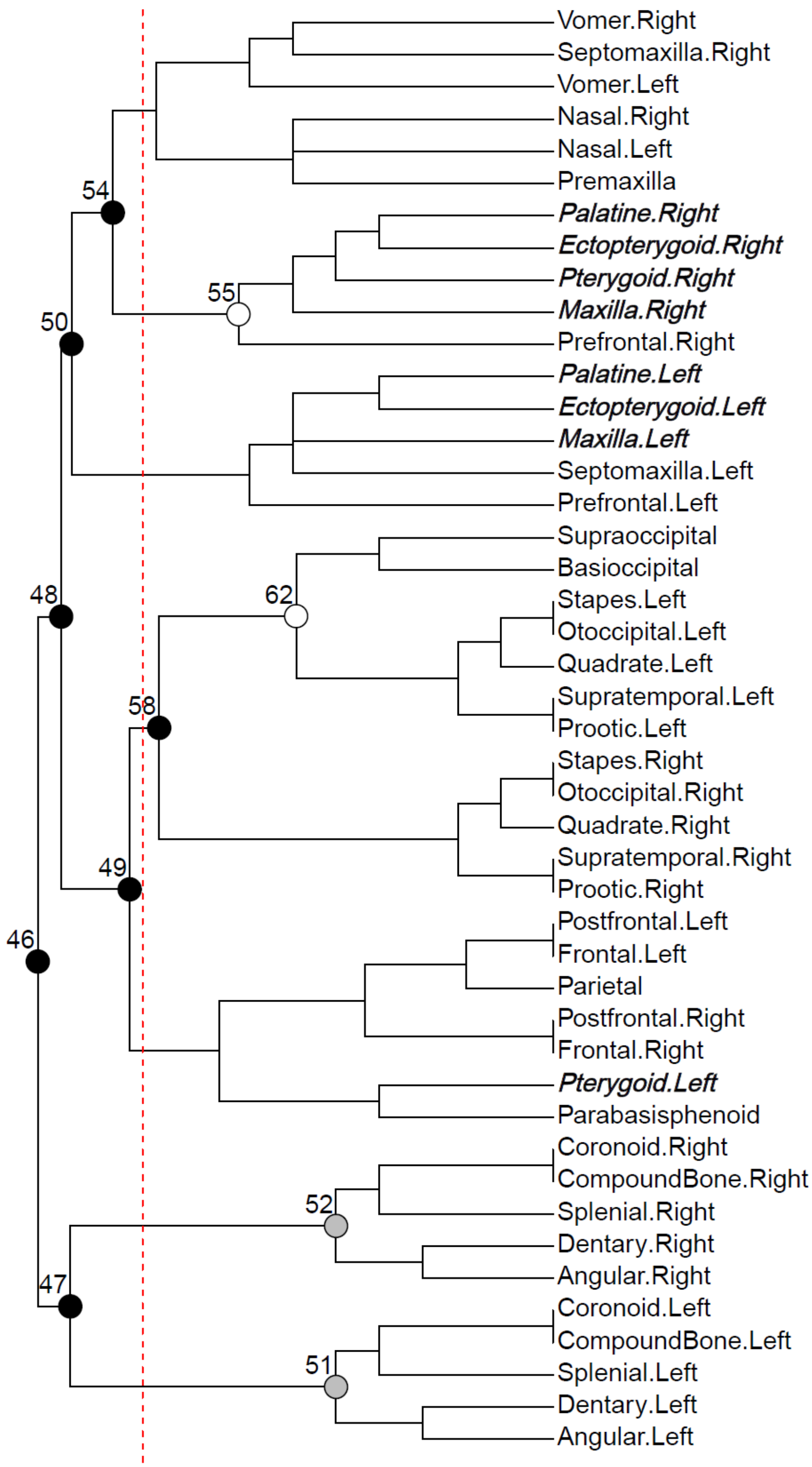

Supplementary Figure S28. Modularity of the skull network of *Cylindrophis ruffus* (UMMZ 201901, UF 143722).

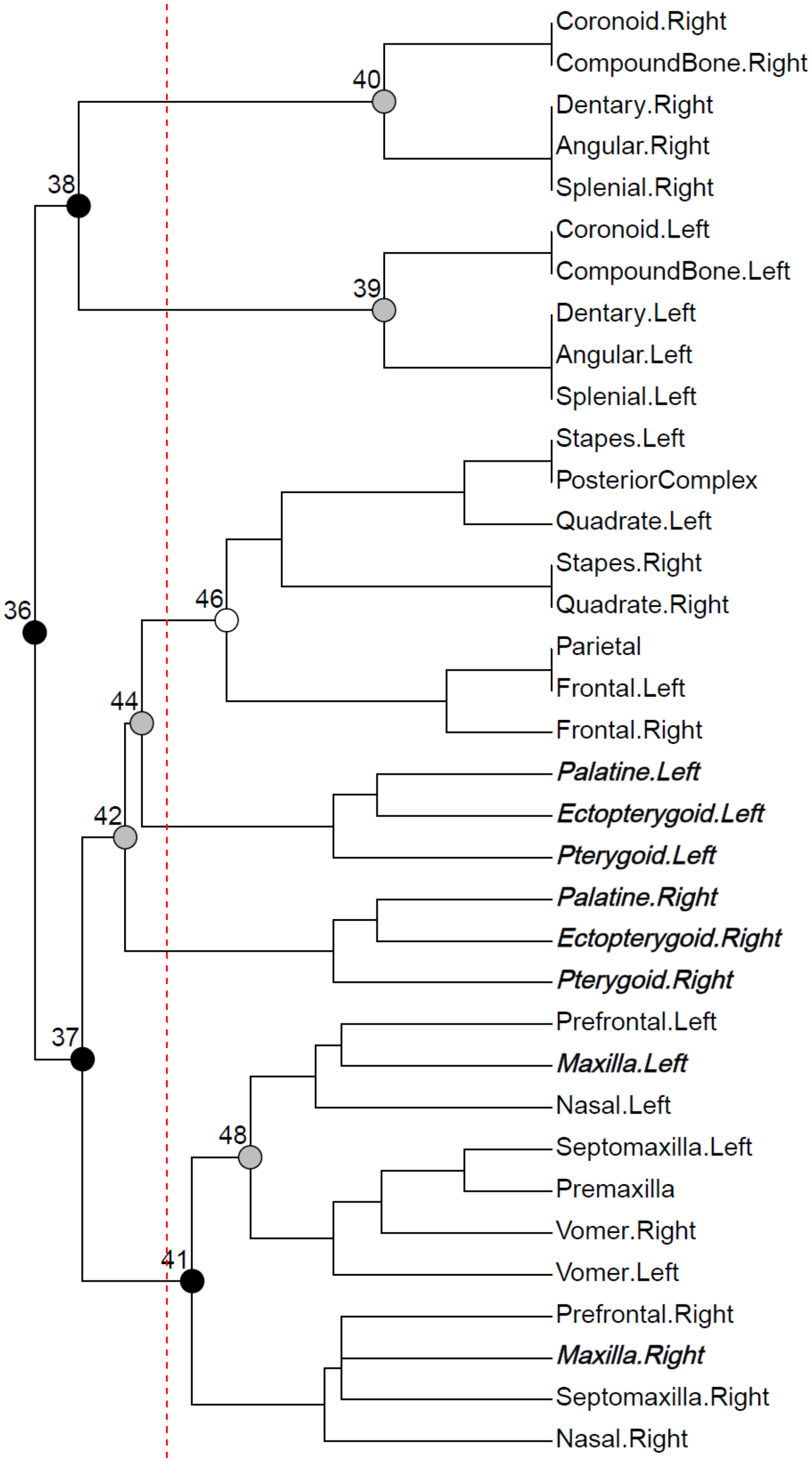

Supplementary Figure S29. Modularity of the skull network of *Rhinophis sanguineus* (UF 78397).

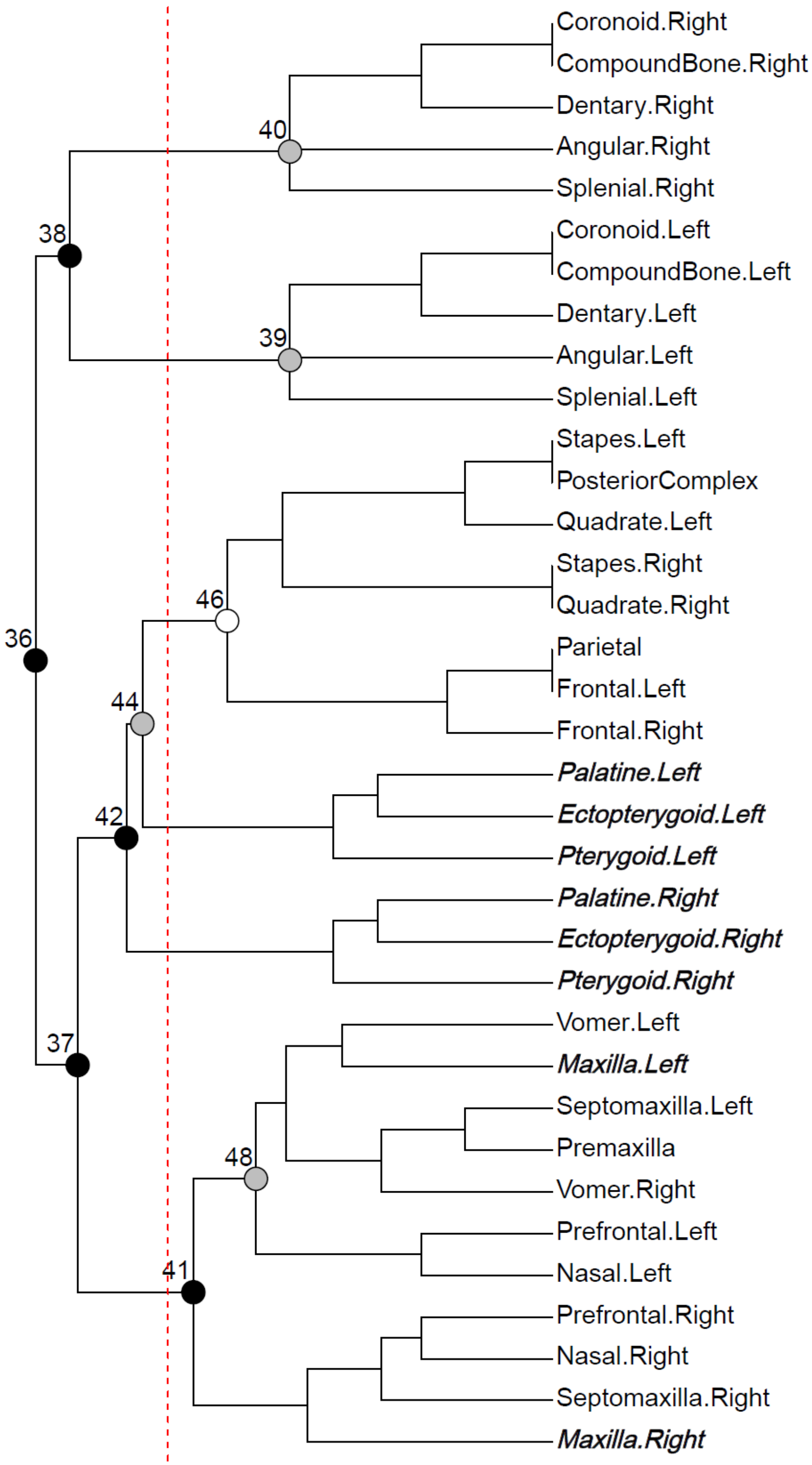

Supplementary Figure S30. Modularity of the skull network of *Uropeltis melanogaster* (FMNH 167048).

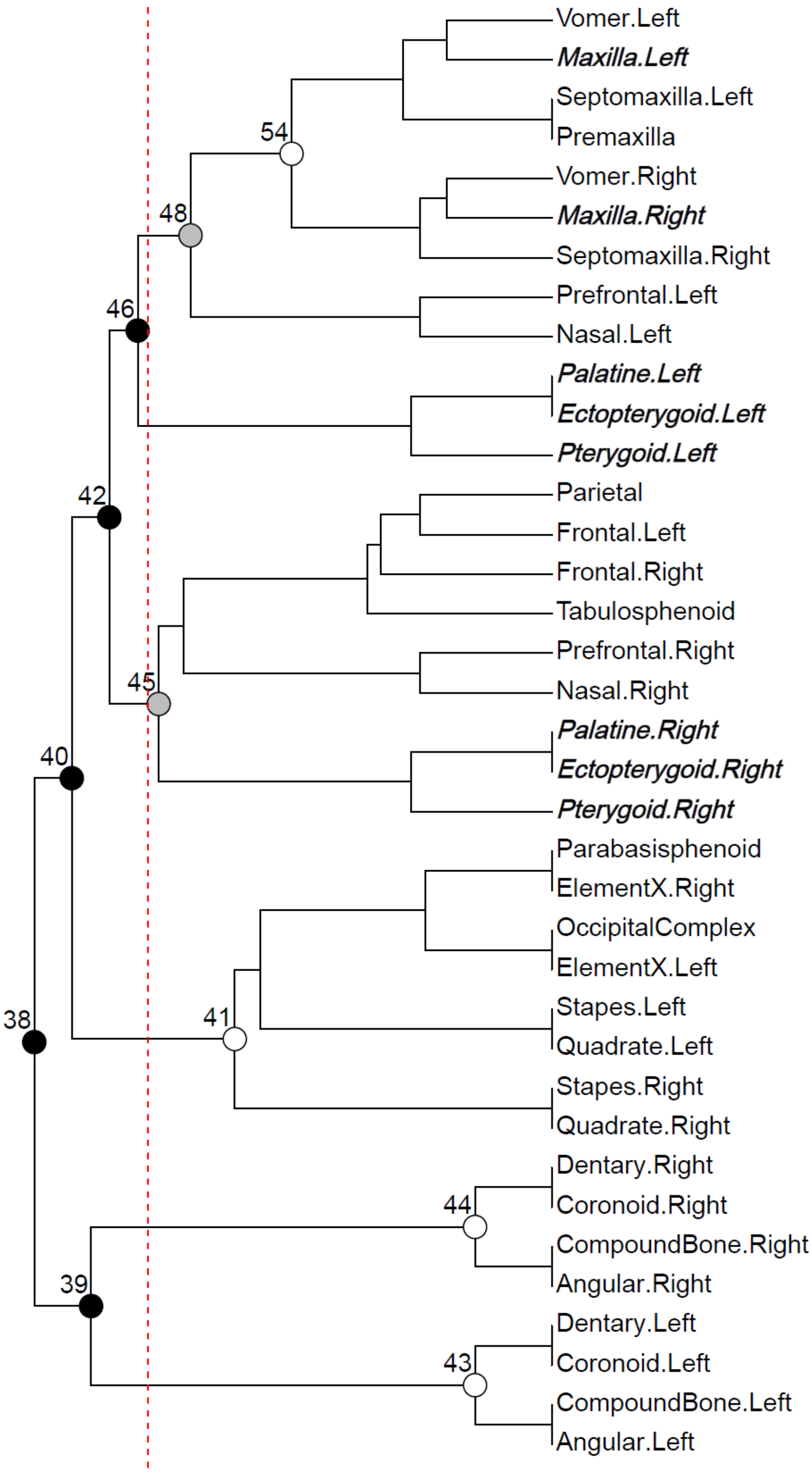

Supplementary Figure S31. Modularity of the skull network of *Amphisbaena fuliginosa* (FMNH 22847).

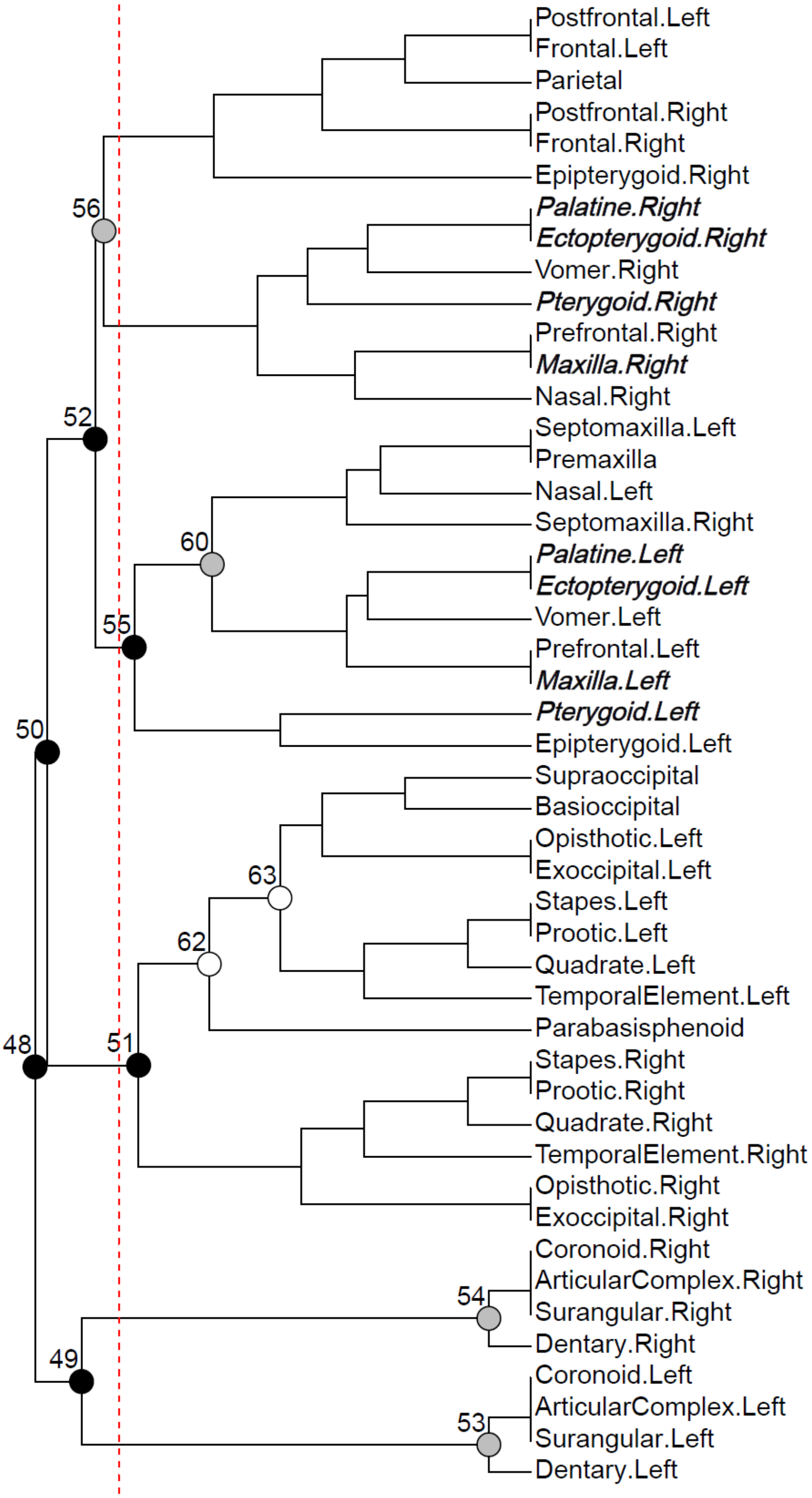

Supplementary Figure S32. Modularity of the skull network of *Anelytropsis papillosus* (TCWC 45501).

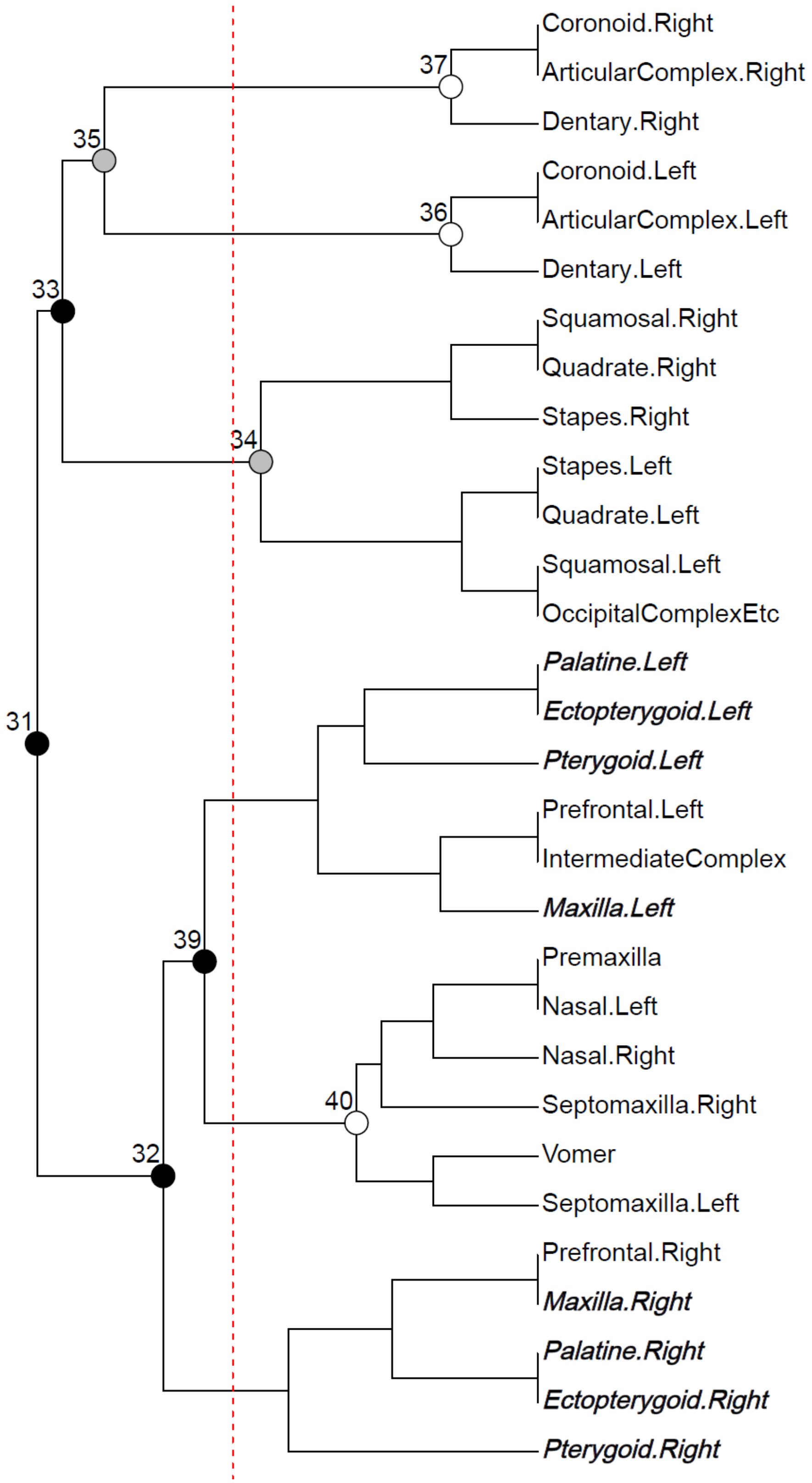

Supplementary Figure S33. Modularity of the skull network of *Bipes biporus* (CAS 126478).

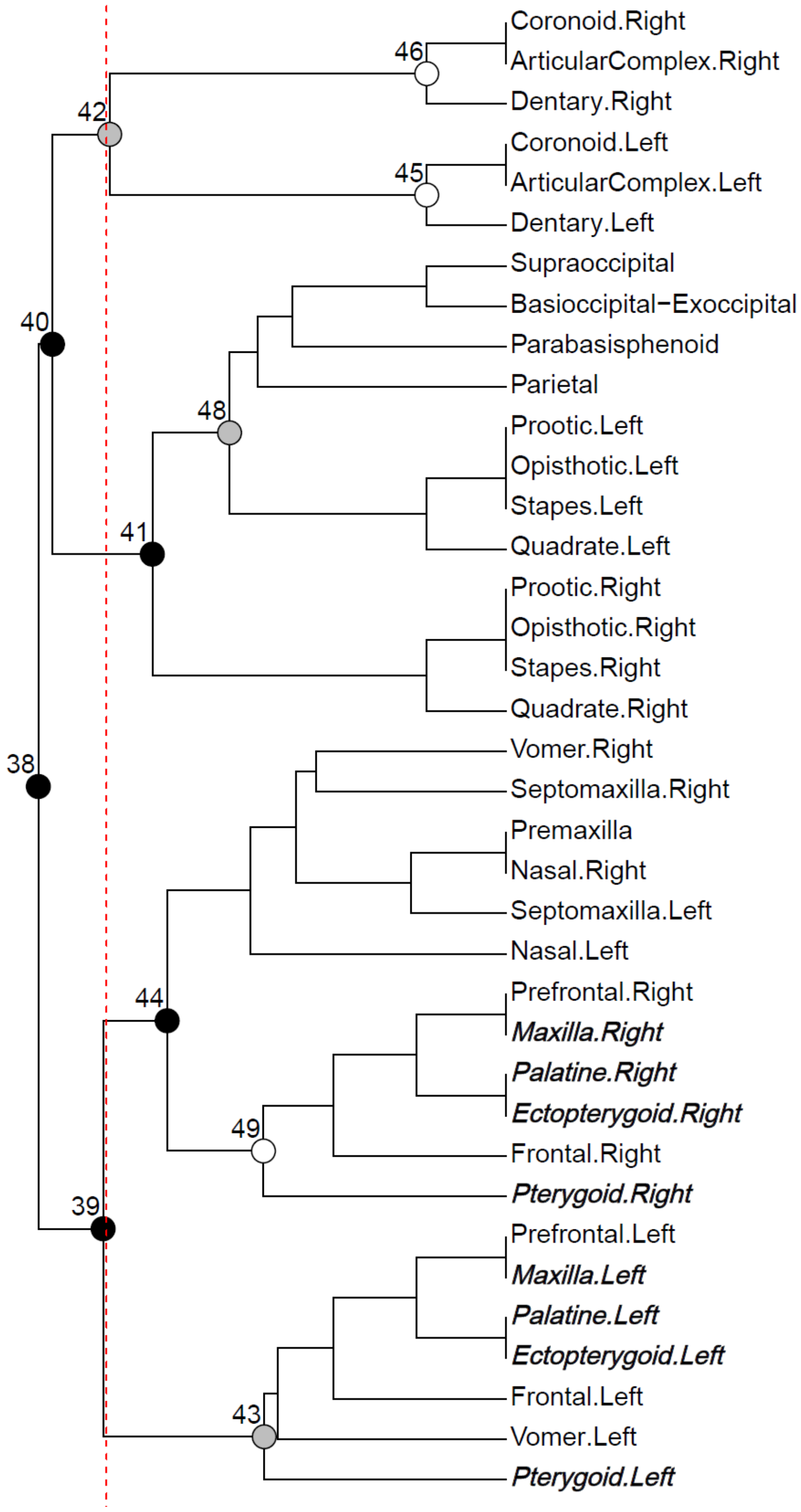

Supplementary Figure S34. Modularity of the skull network of *Dibamus novaeguineae* (UF 33488).

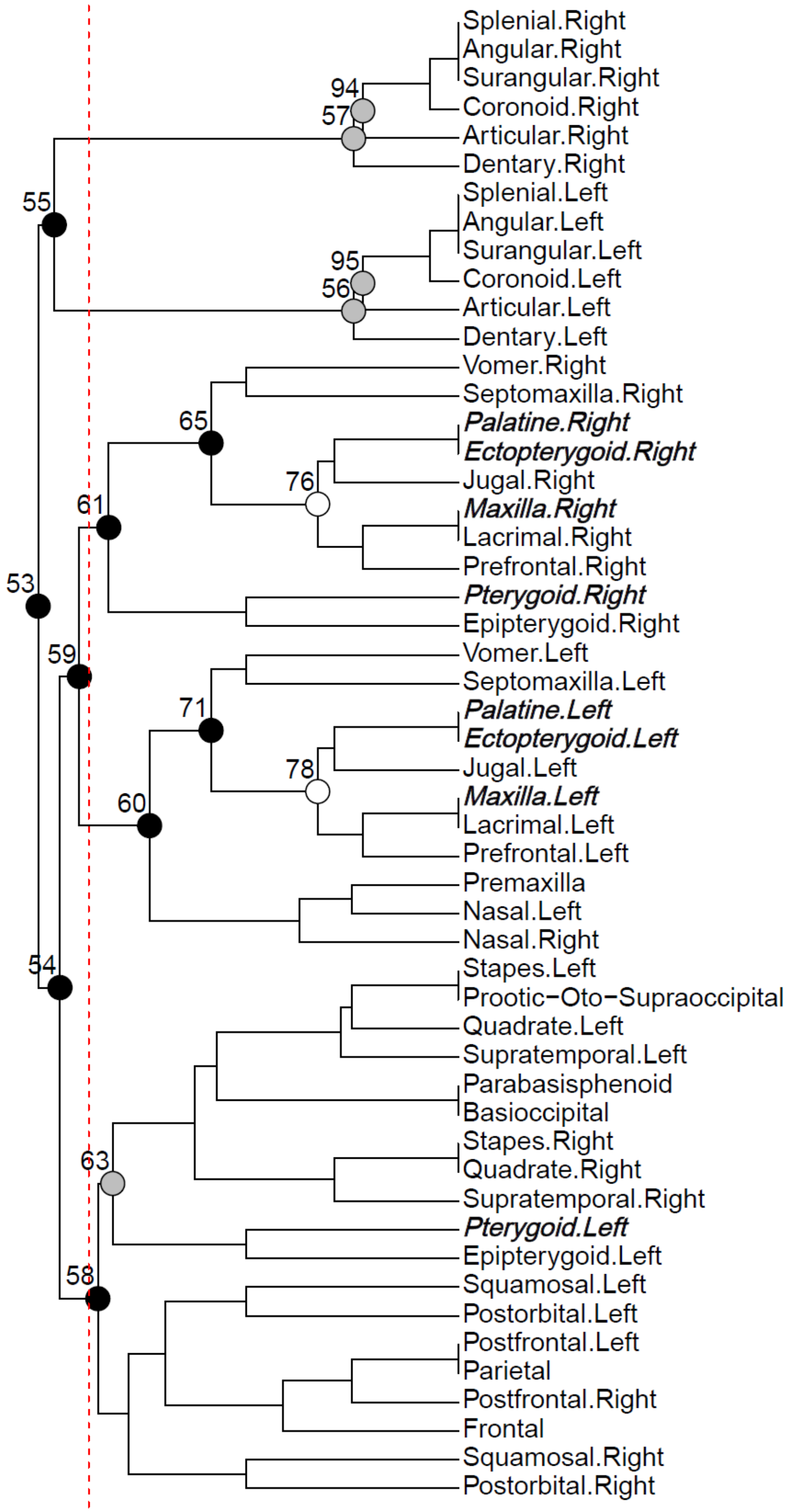

Supplementary Figure S35. Modularity of the skull network of *Dipsosaurus dorsalis* (YPM 14376).

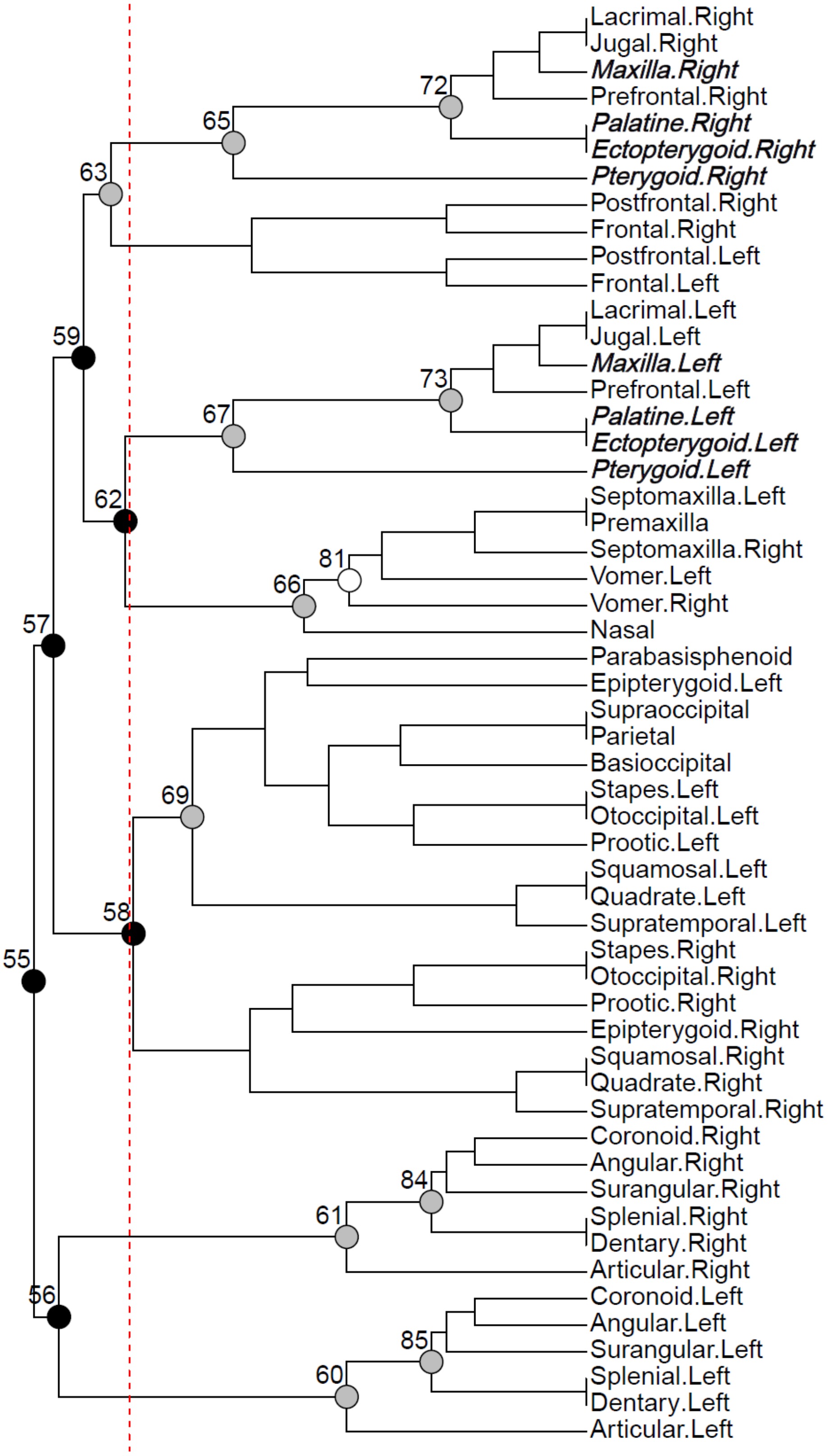

Supplementary Figure S36. Modularity of the skull network of *Lanthanotus borneensis* (FMNH 148589).

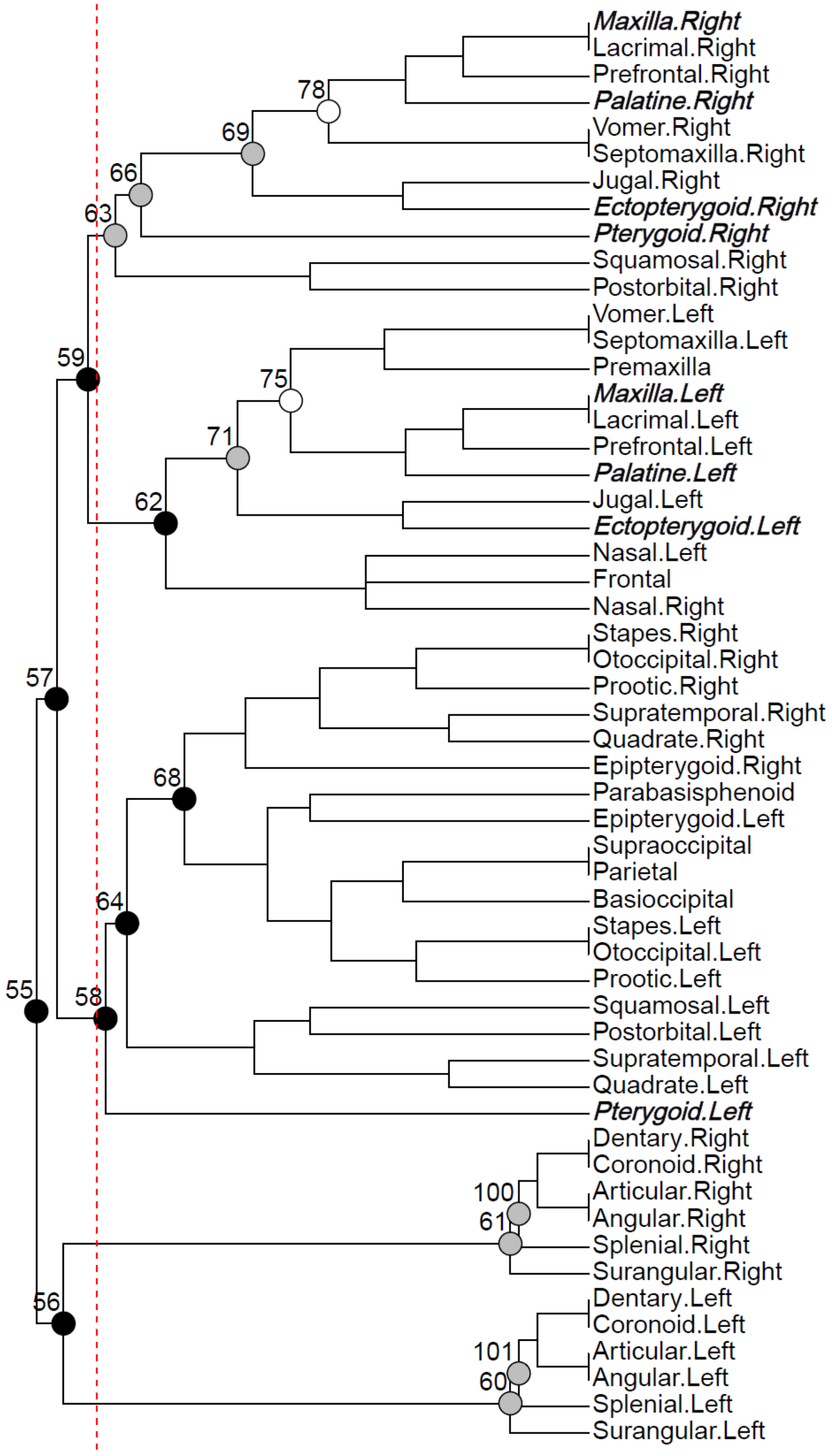

Supplementary Figure S37. Modularity of the skull network of *Physignathus cocincinus* (YPM 14378).

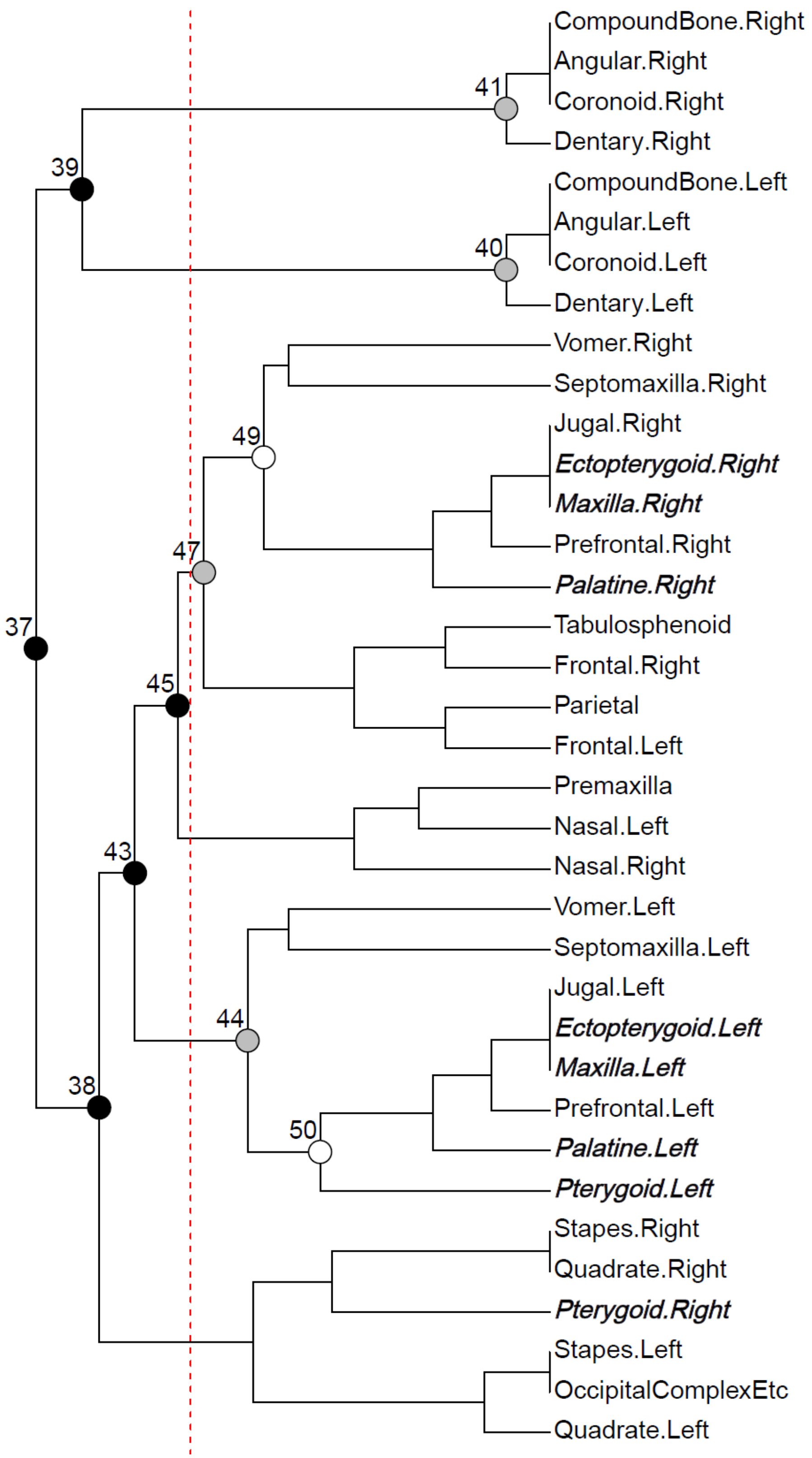

Supplementary Figure S38. Modularity of the skull network of *Rhineura floridana* (FMNH 31774).

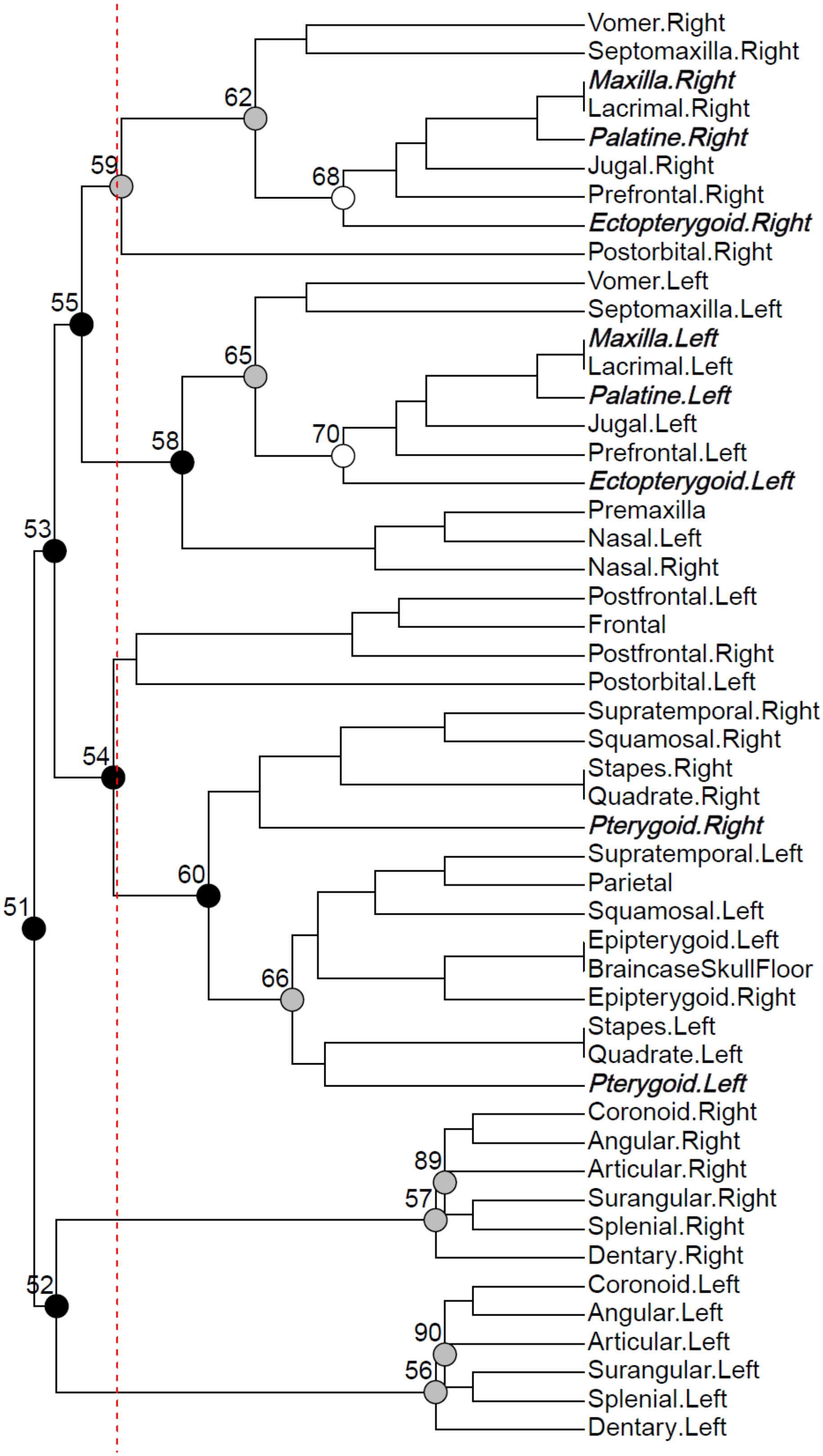

Supplementary Figure S39. Modularity of the skull network of *Sauromalus ater* (TNHC 18483).

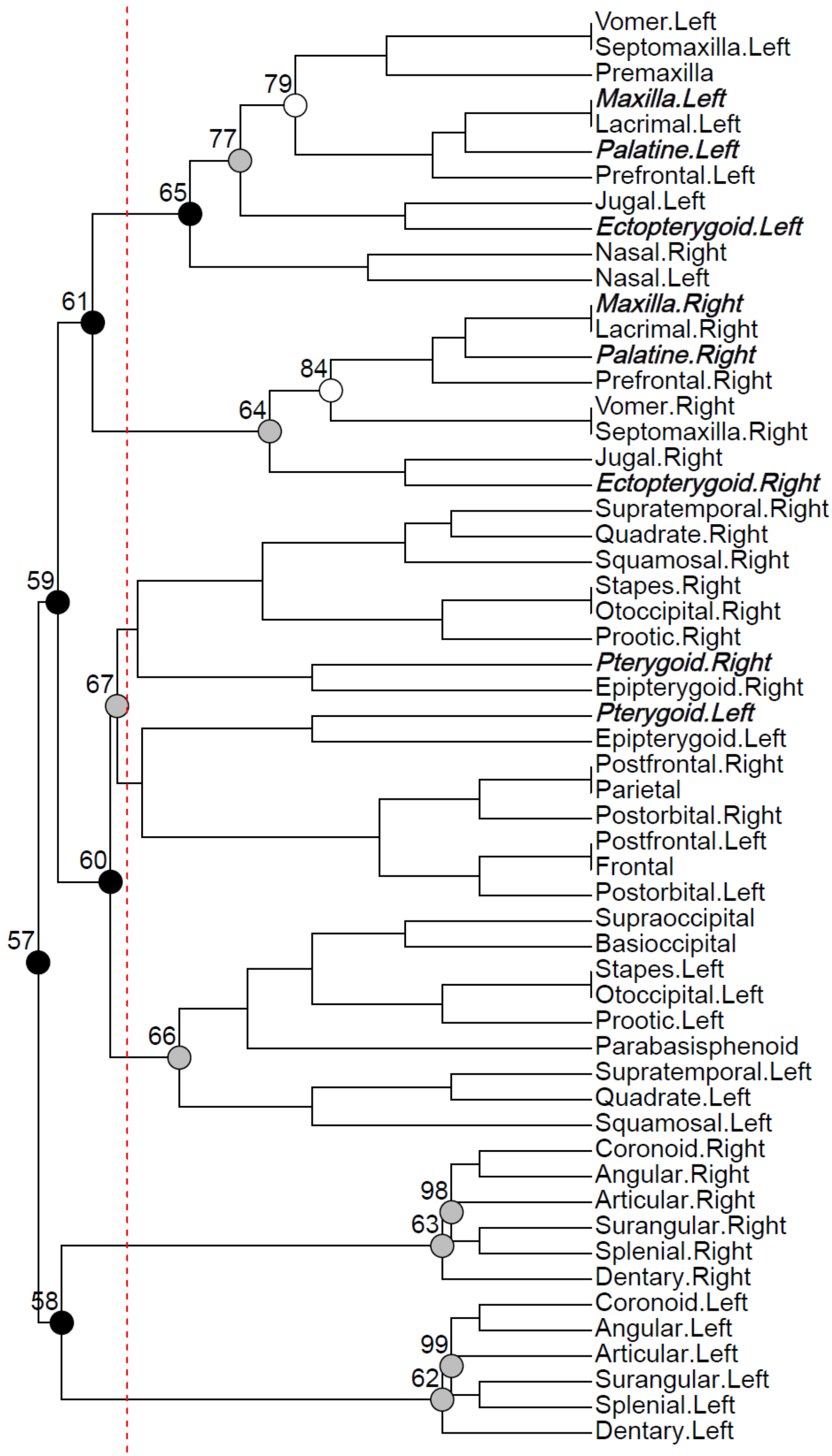

Supplementary Figure S40. Modularity of the skull network of *Uranoscodon superciliosus* (YPM 12871).

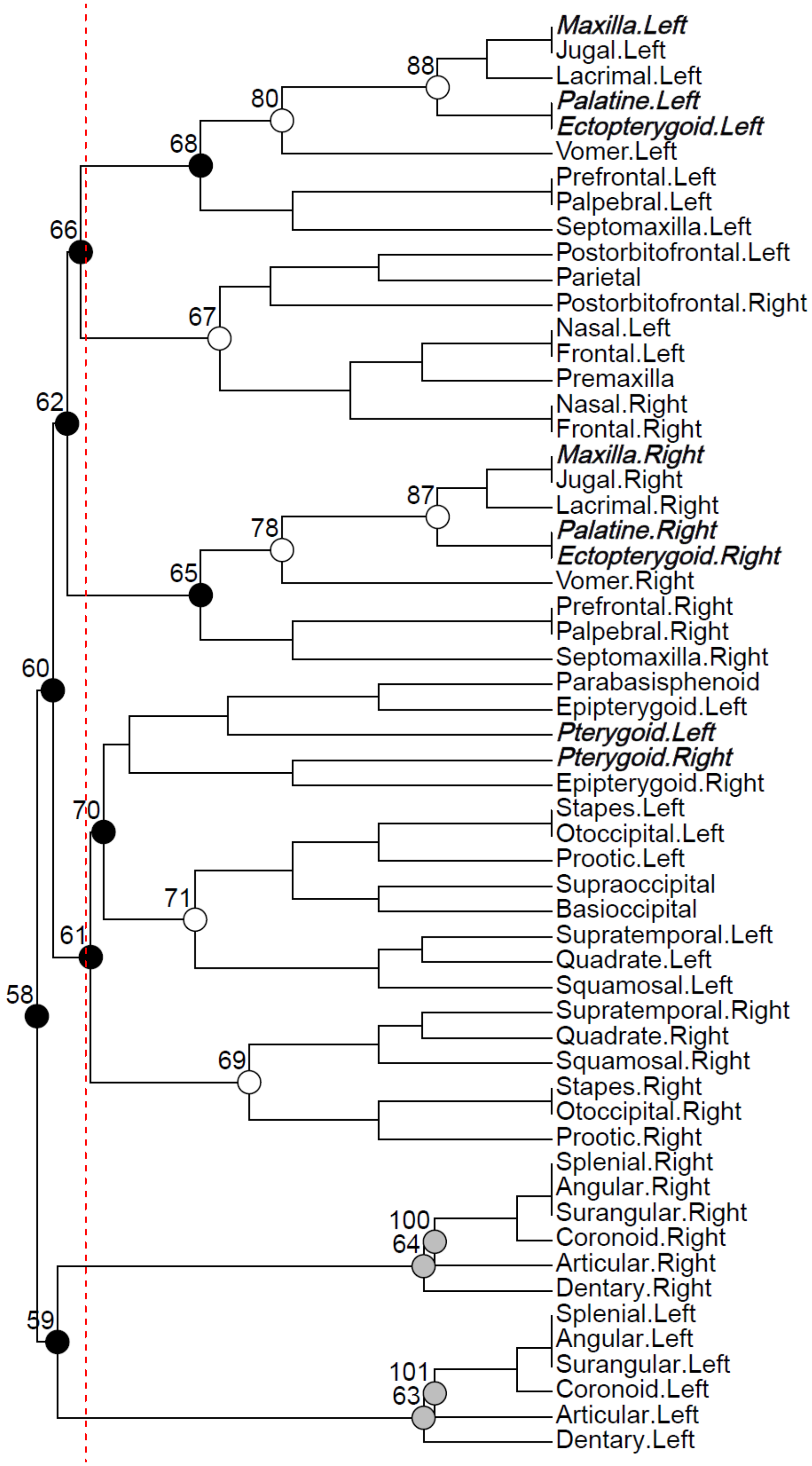

Supplementary Figure S41. Modularity of the skull network of *Varanus exanthematicus* (FMNH 58299).

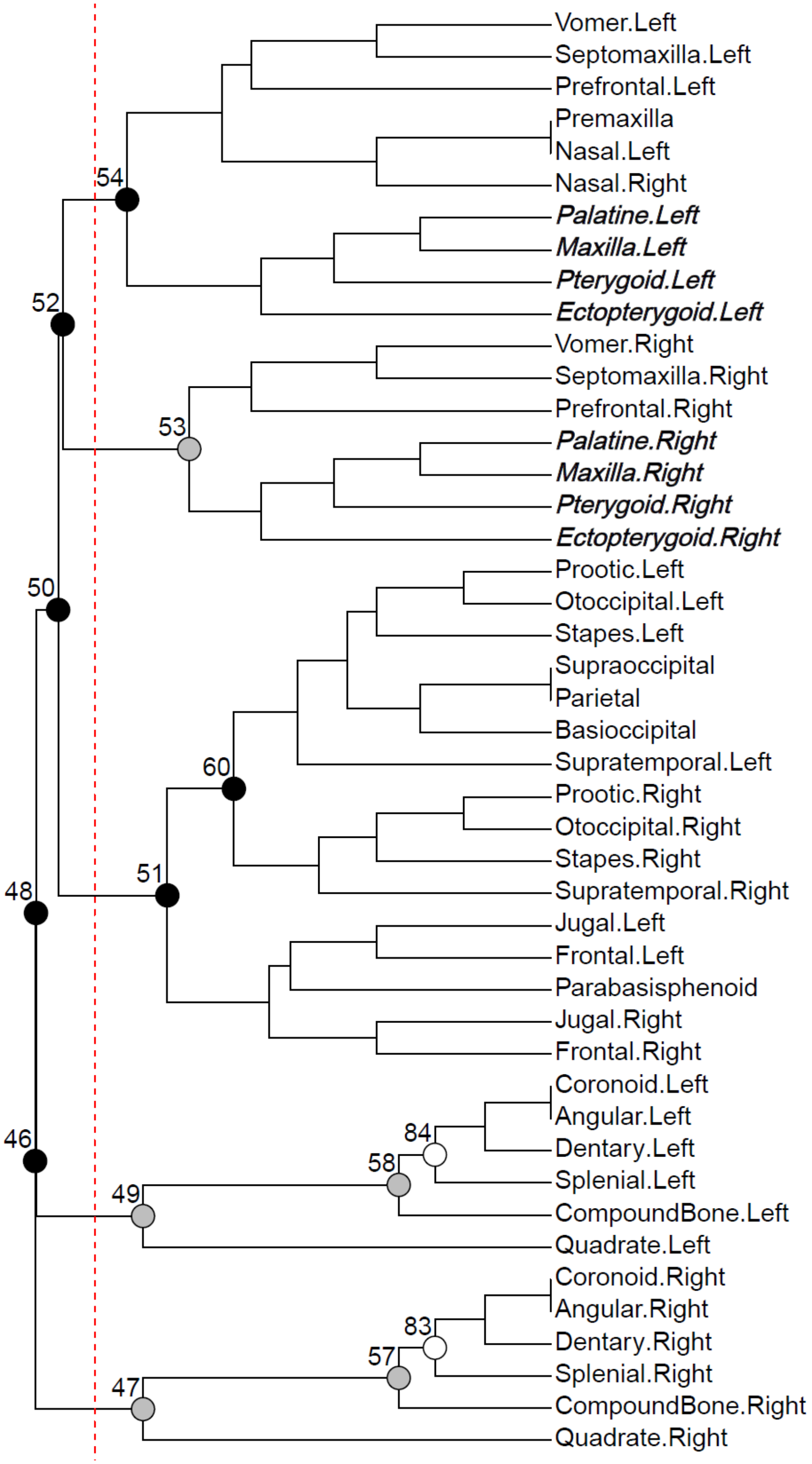

Supplementary Figure S42. Modularity of the skull network of *Boa constrictor* (FMNH 31182).

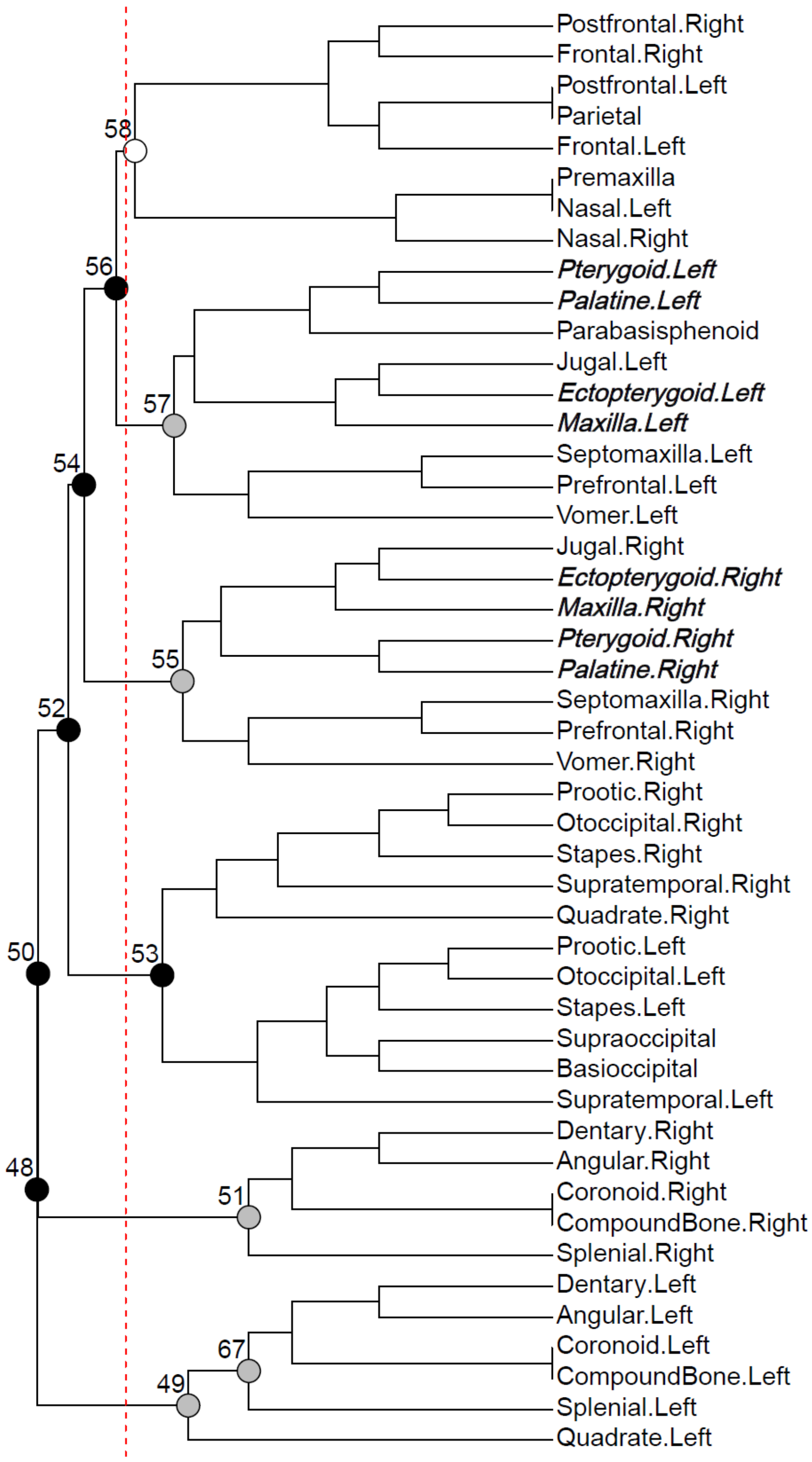

Supplementary Figure S43. Modularity of the skull network of *Calabaria reinhardtii* (FMNH 117833).

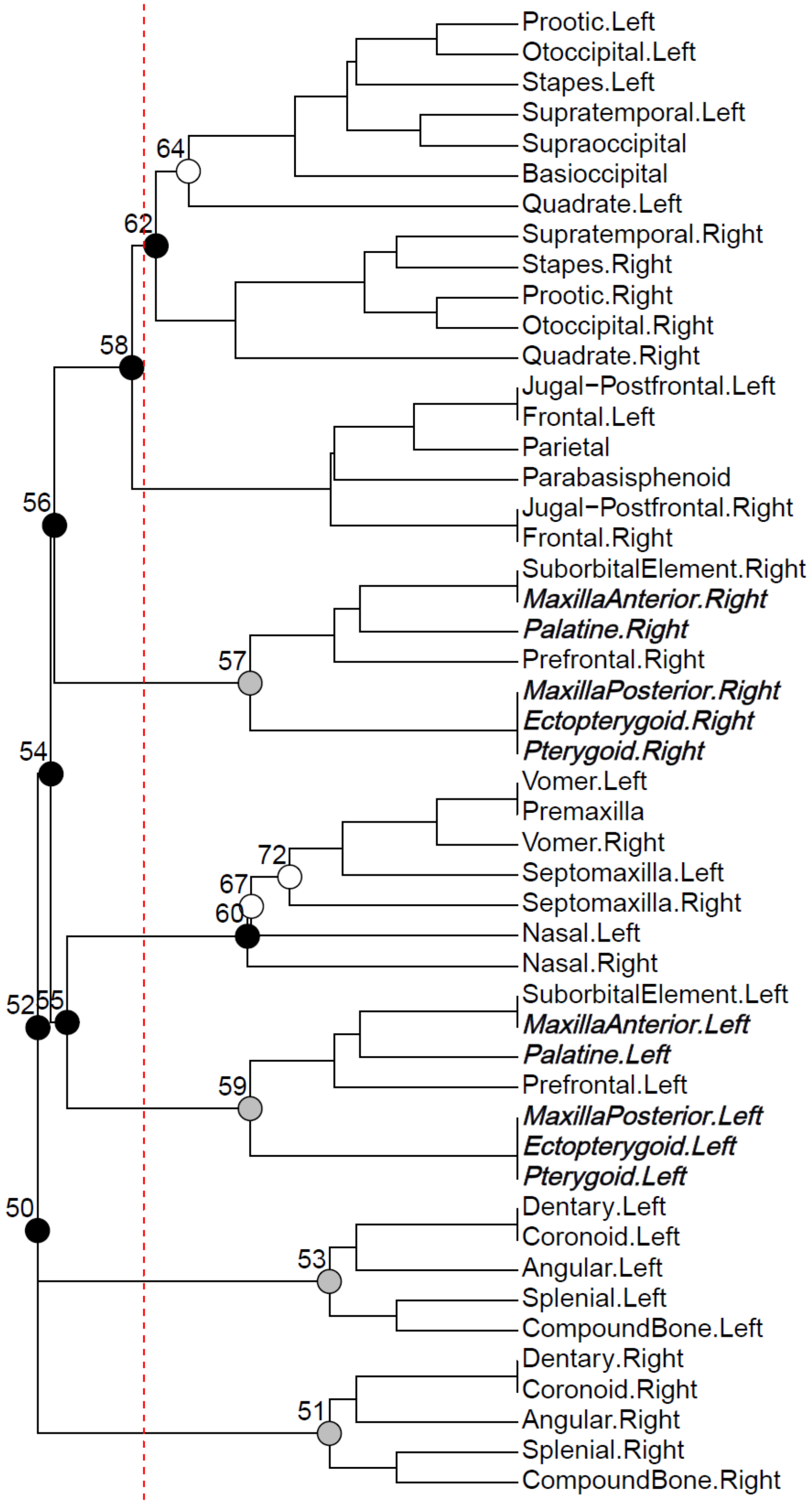

Supplementary Figure S44. Modularity of the skull network of *Casarea dussumieri* (UMMZ 190285).

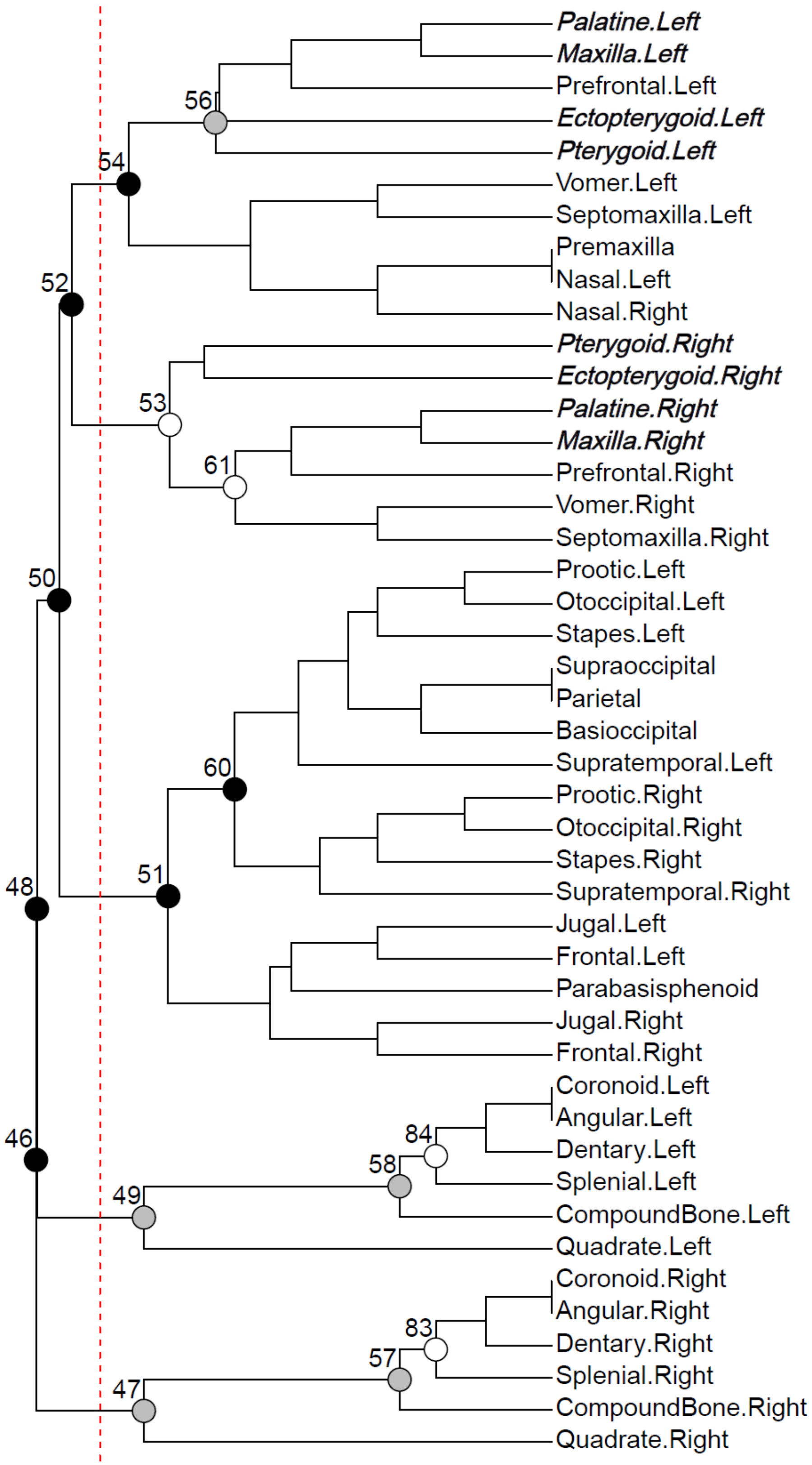

Supplementary Figure S45. Modularity of the skull network of *Eryx colubrinus* (FMNH 63117).

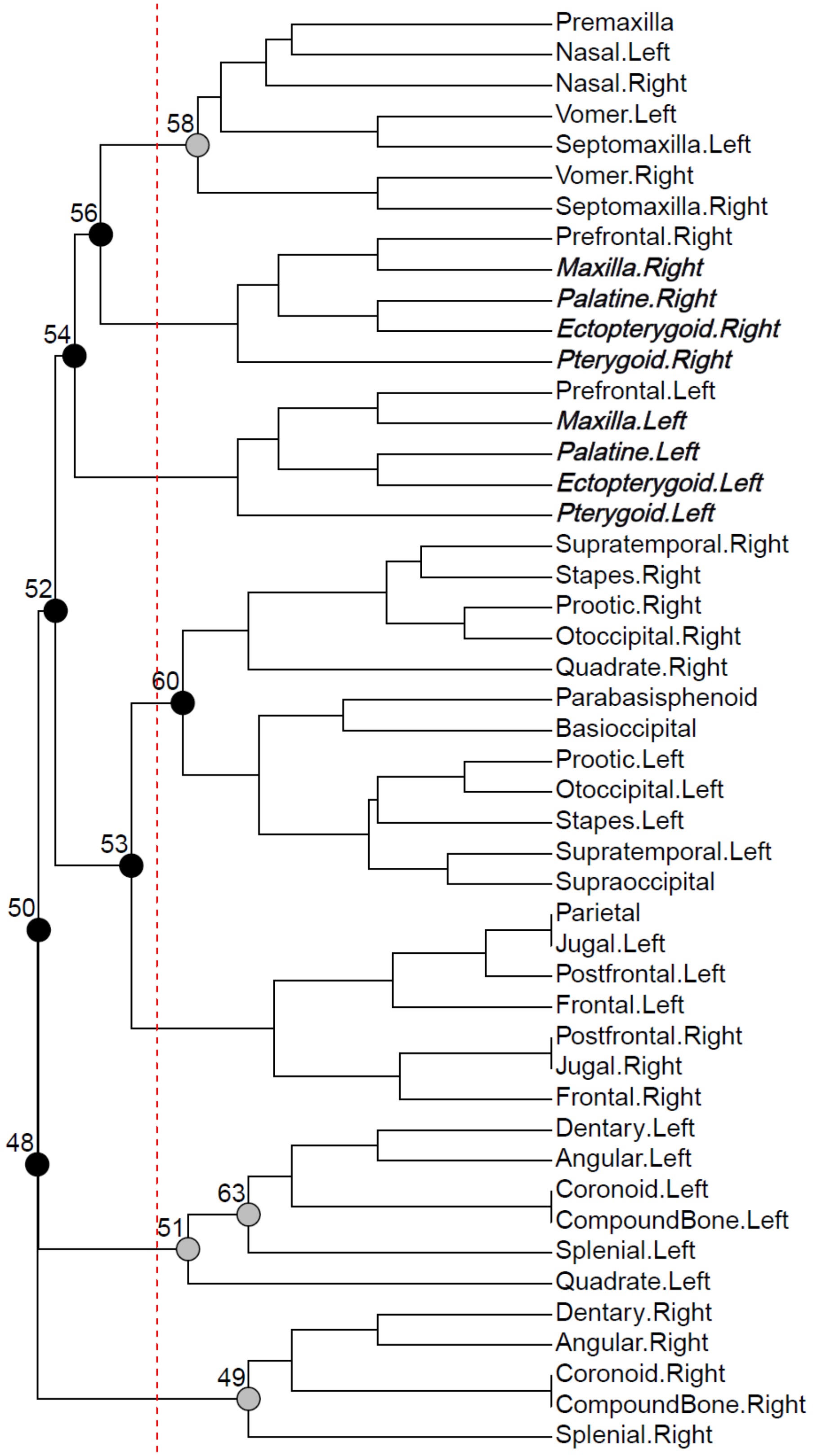

Supplementary Figure S46. Modularity of the skull network of *Loxocemus bicolor* (FMNH 104800).

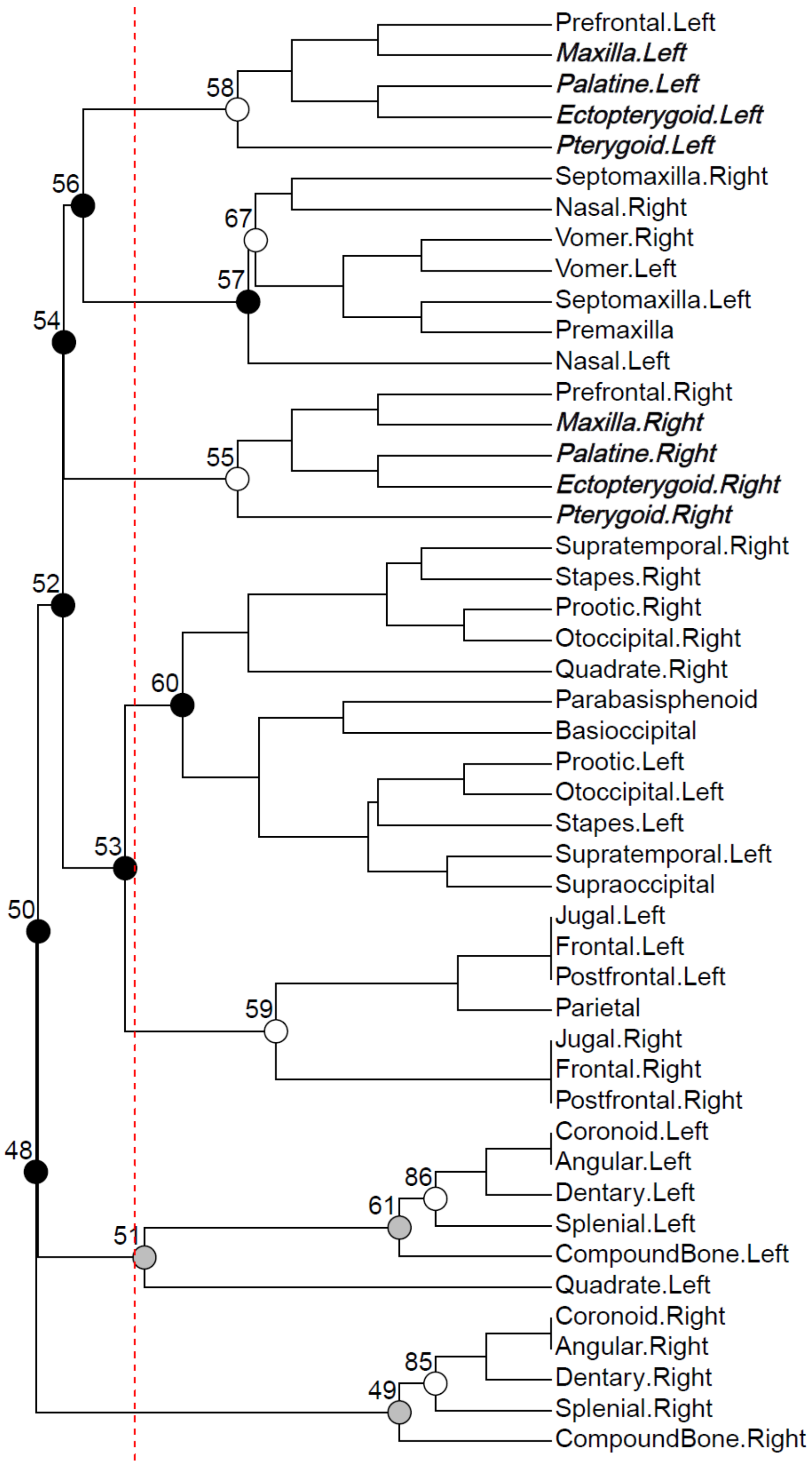

Supplementary Figure S47. Modularity of the skull network of *Python molurus* (TNHC 62769).

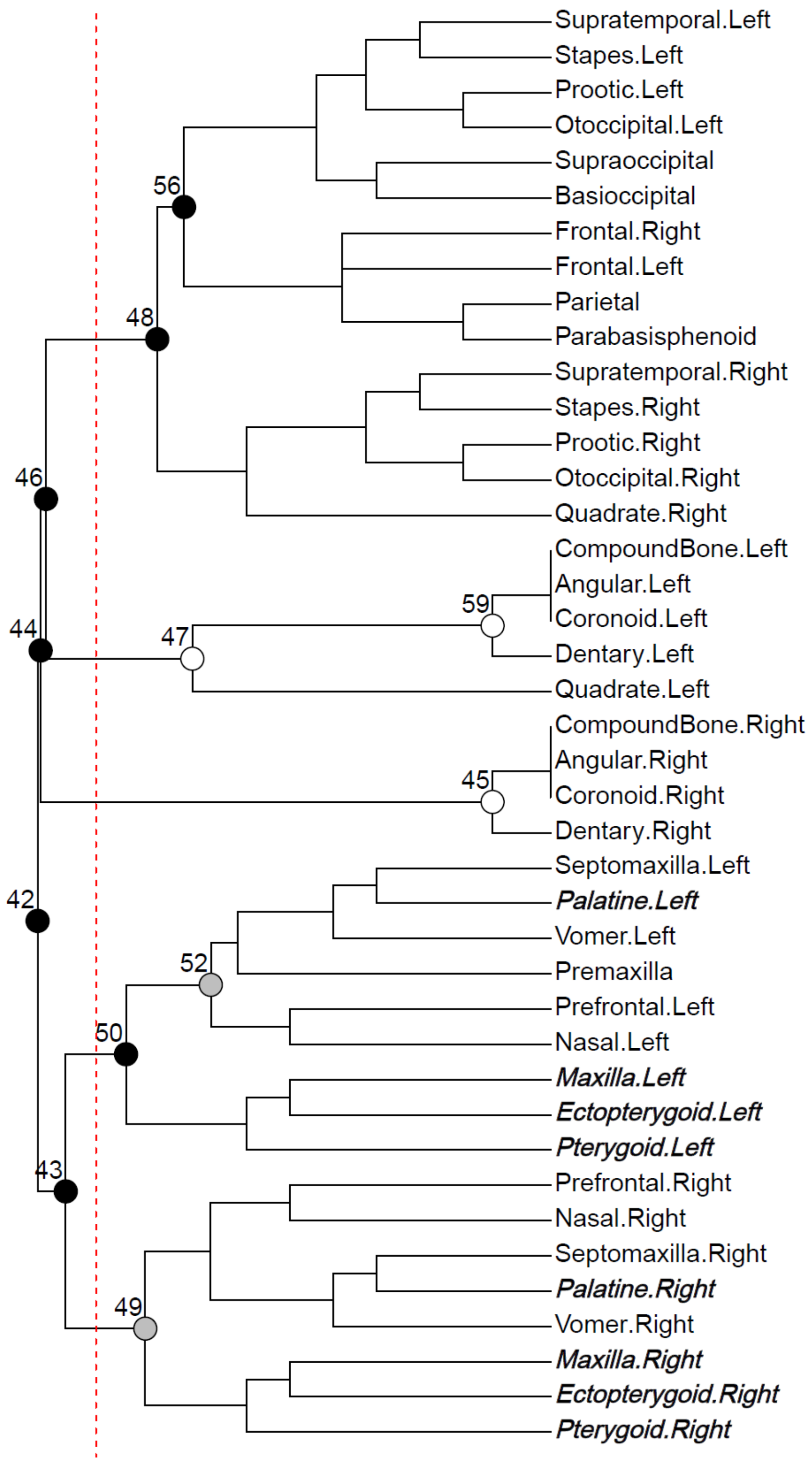

Supplementary Figure S48. Modularity of the skull network of *Xenopeltis unicolor* (FMNH 148900).

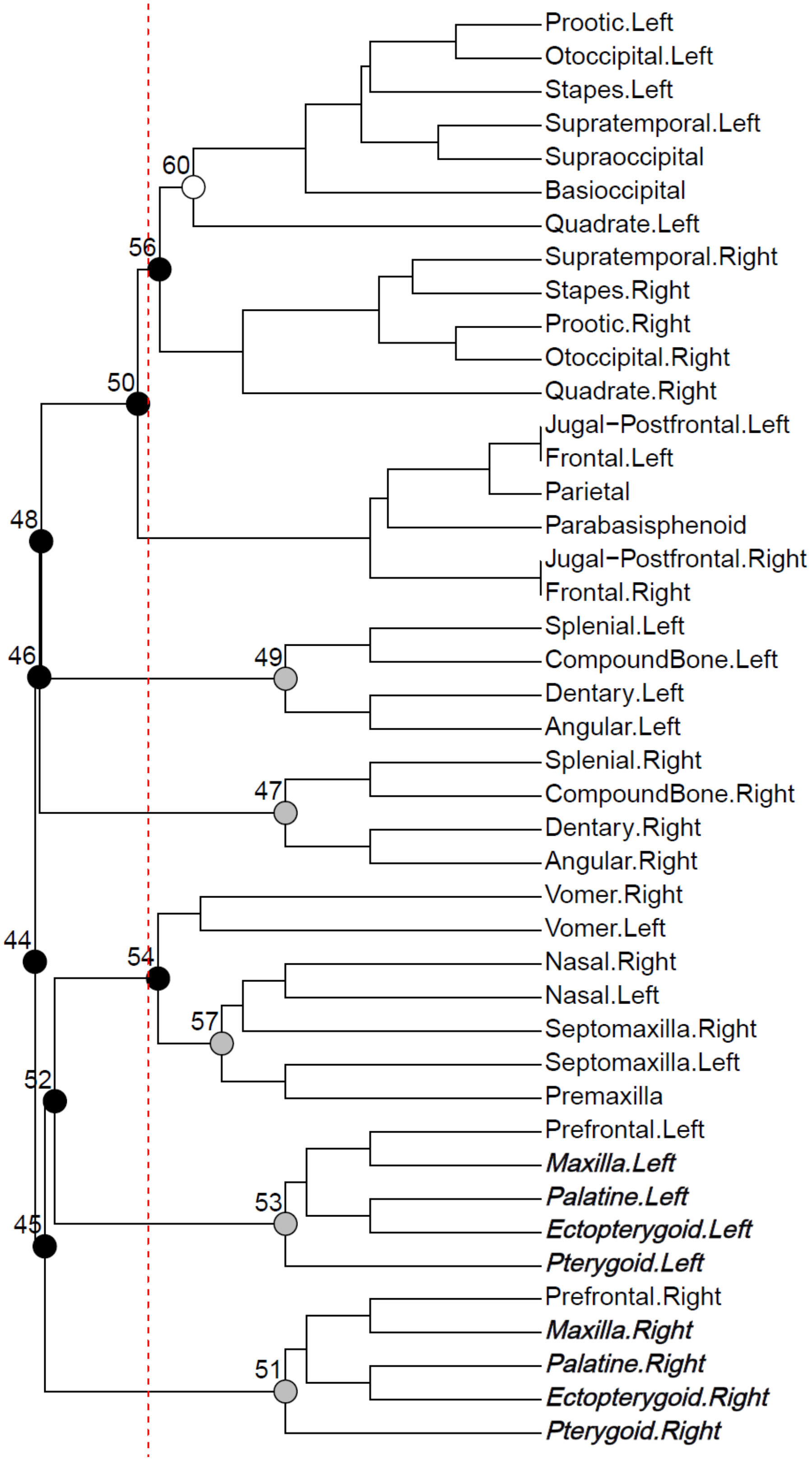

Supplementary Figure S49. Modularity of the skull network of *Acrochordus granulatus* (MCZ R-146128).

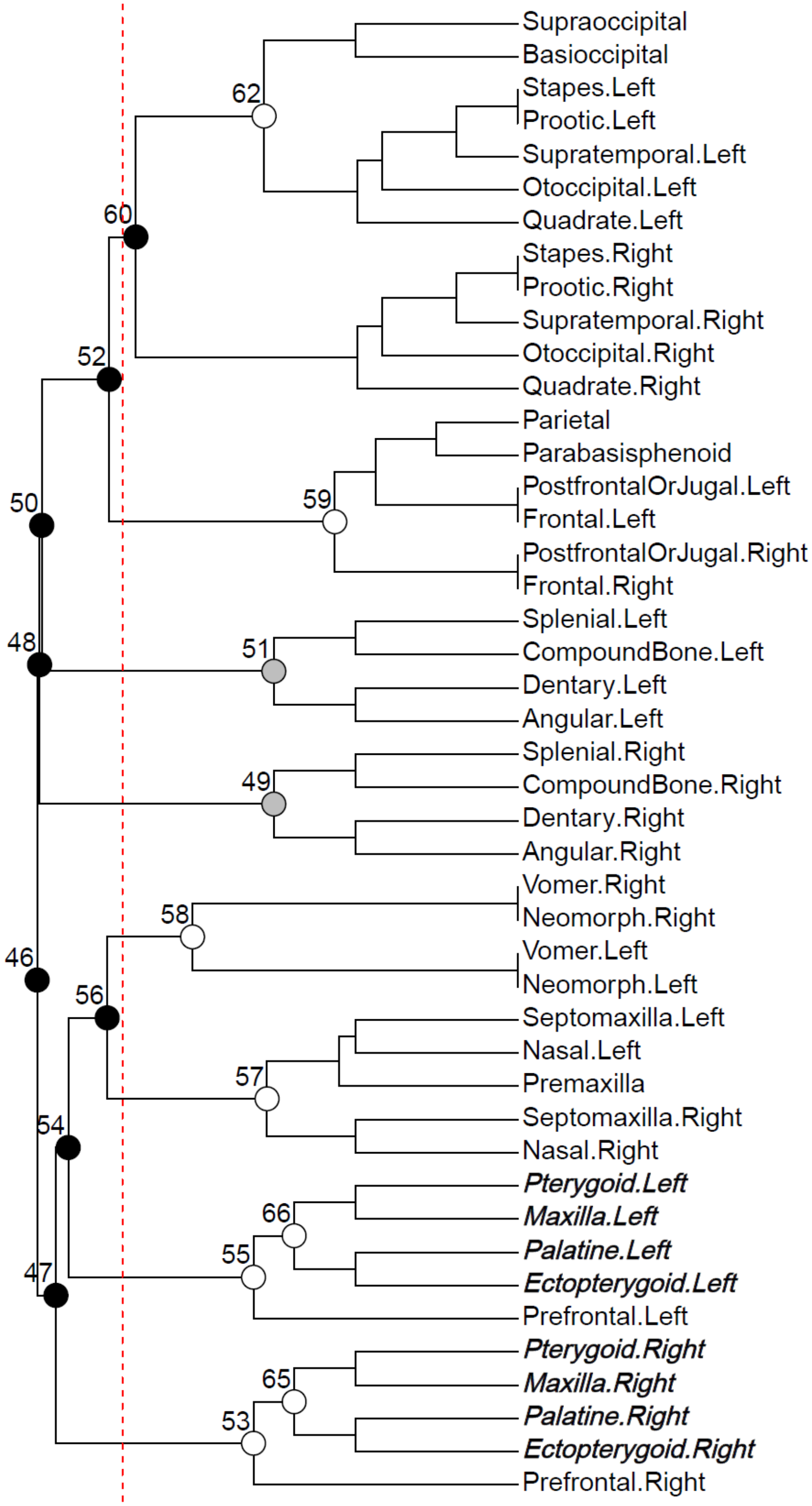

Supplementary Figure S50. Modularity of the skull network of *Aparallactus guentheri* (MCZ R-23363).

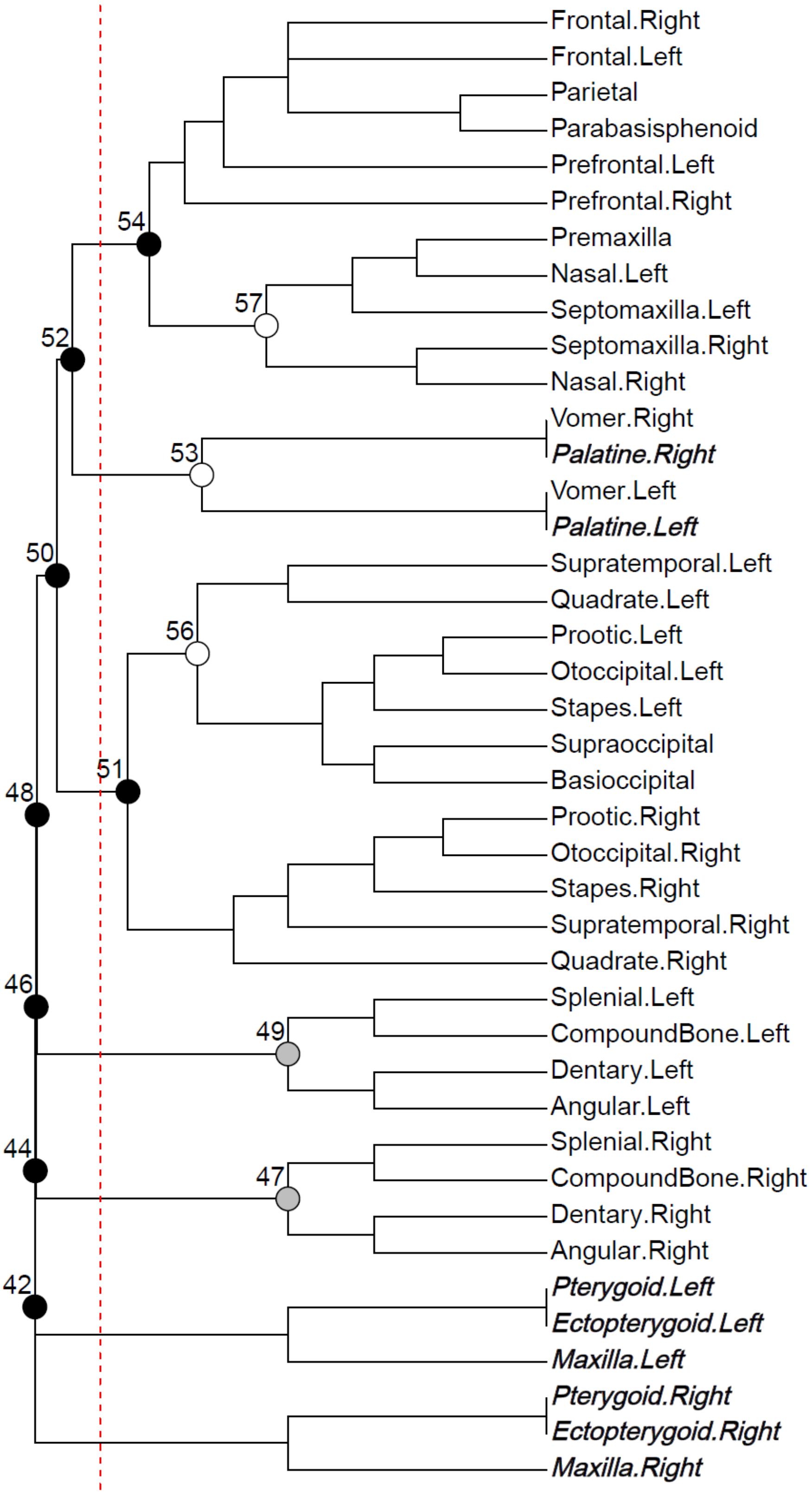

Supplementary Figure S51. Modularity of the skull network of *Atractaspis irregularis* (FMNH 62204, UF 68168).

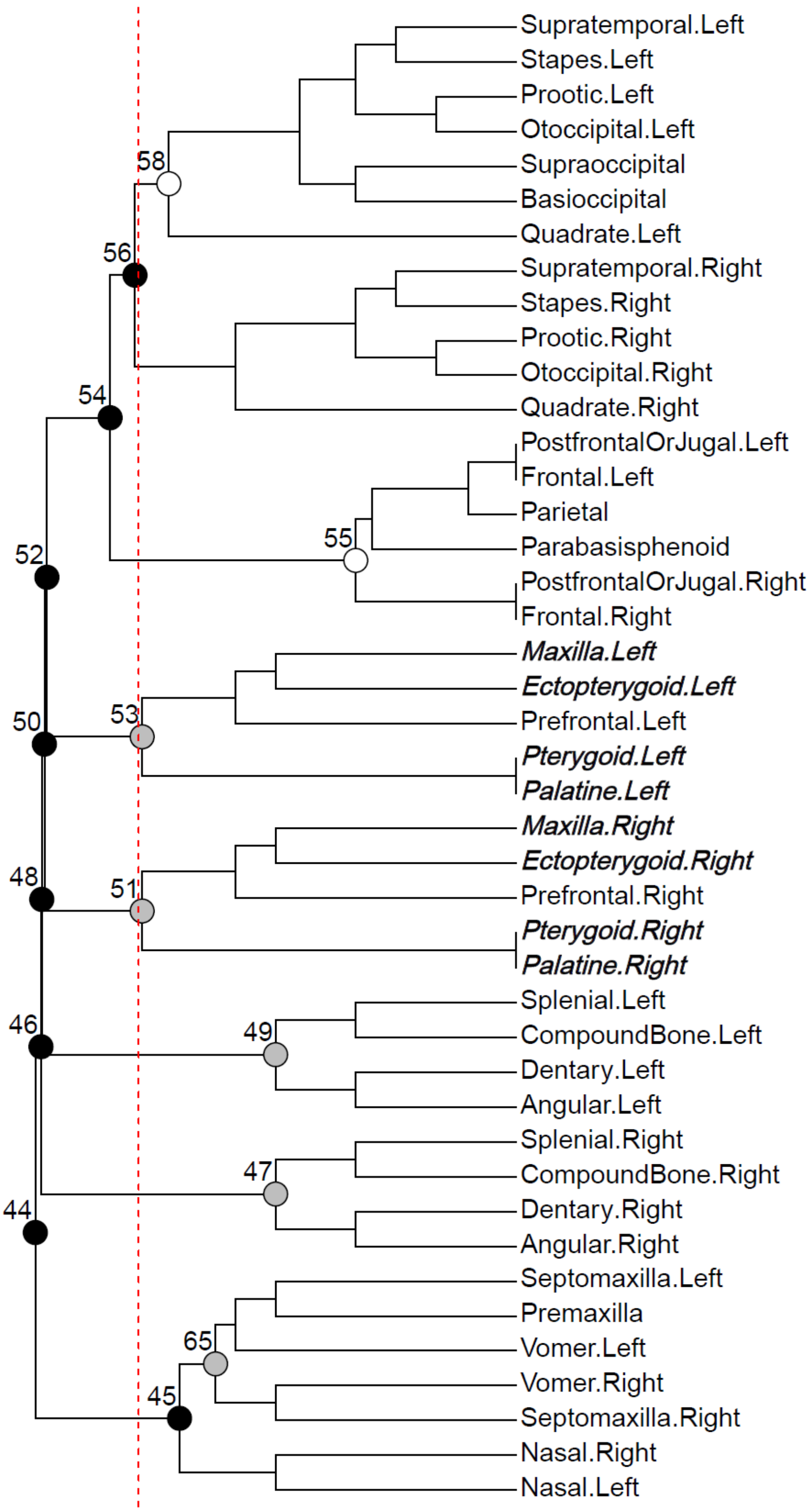

Supplementary Figure S52. Modularity of the skull network of *Crotalus adamanteus* (UF 103268).

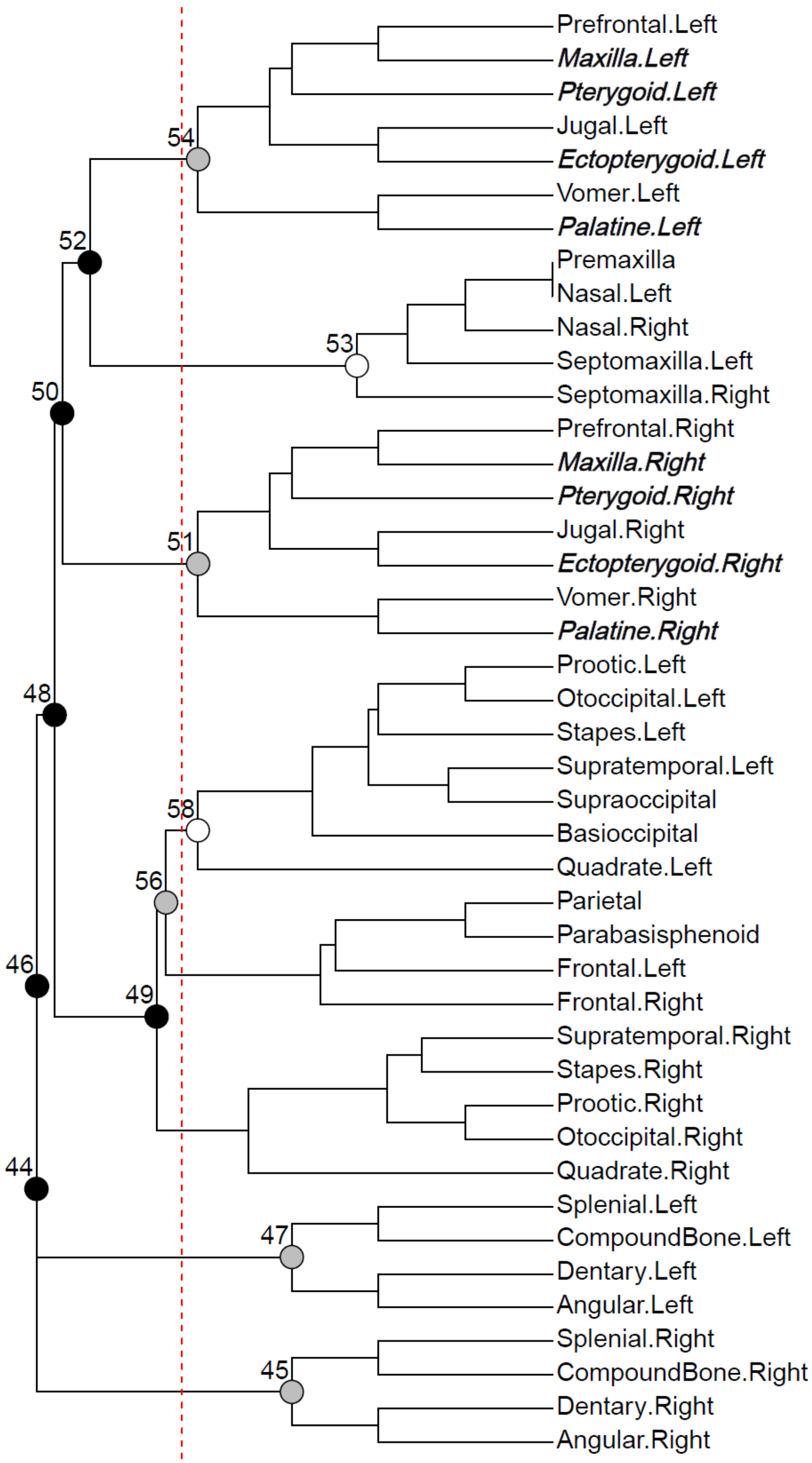

Supplementary Figure S53. Modularity of the skull network of *Homalopsis buccata* (FMNH 259340).

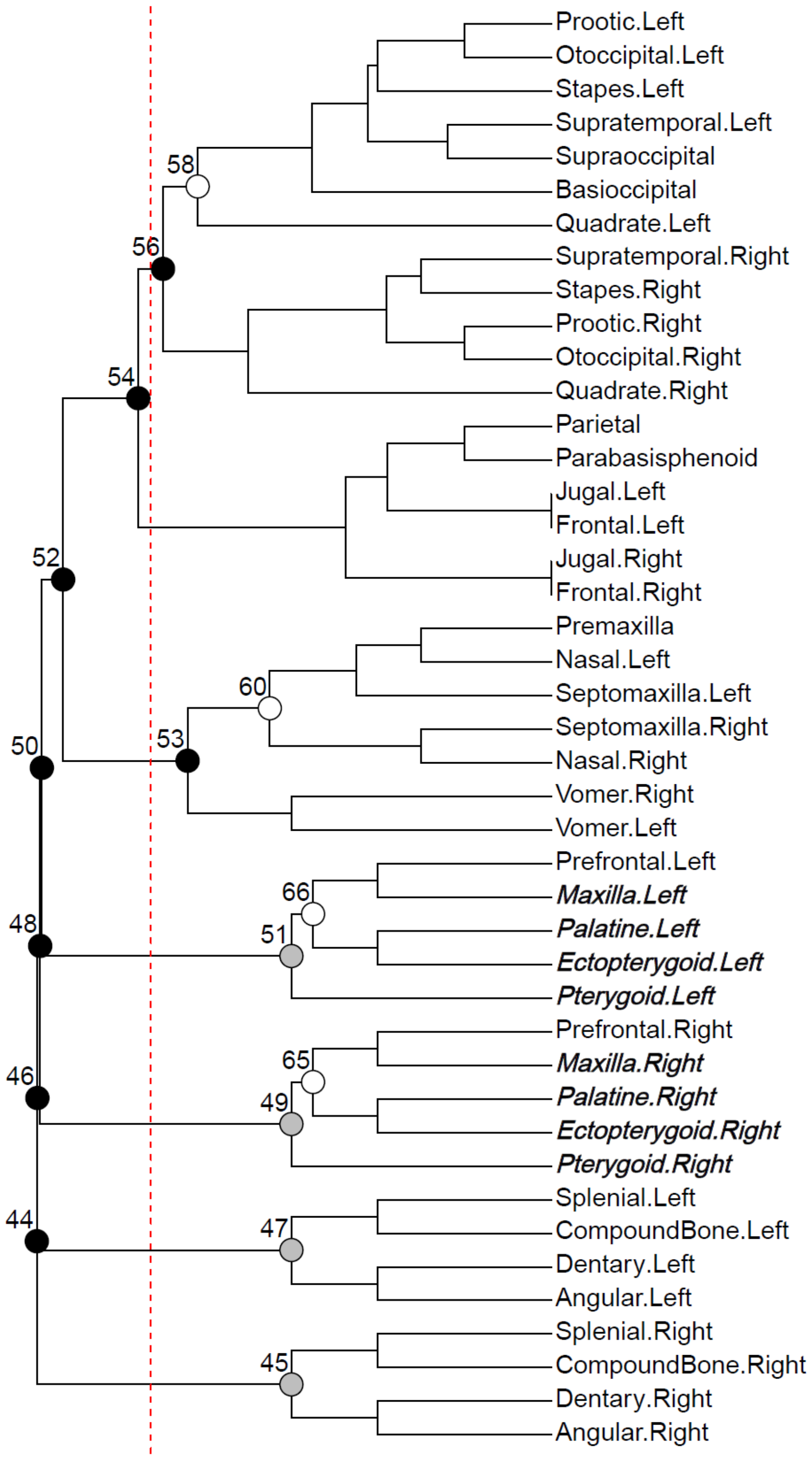

Supplementary Figure S54. Modularity of the skull network of *Lampropeltis getula* (FMNH 95184).

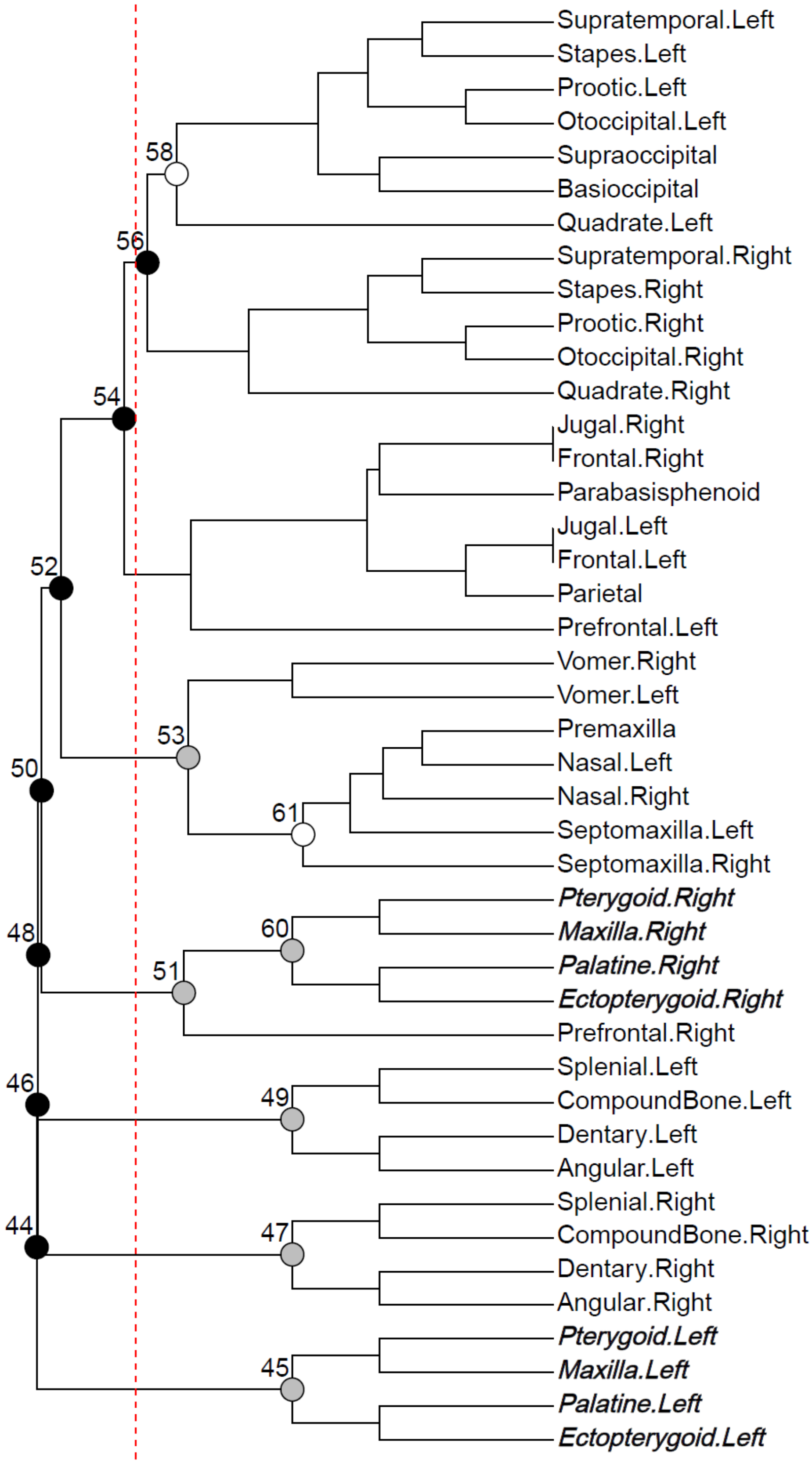

Supplementary Figure S55. Modularity of the skull network of *Naja naja* (FMNH 22468).

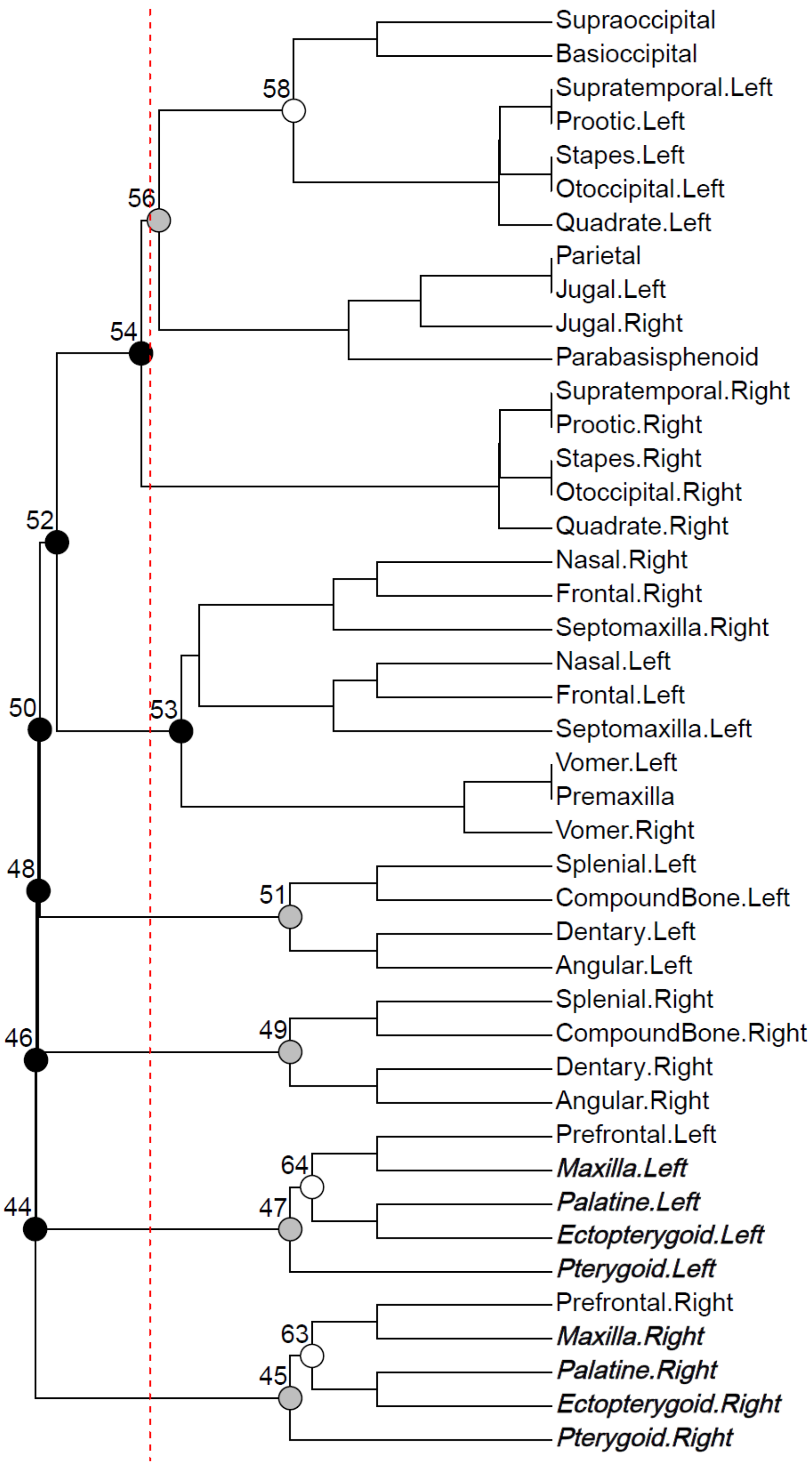

Supplementary Figure S56. Modularity of the skull network of *Pareas hamptoni* (FMNH 128304).

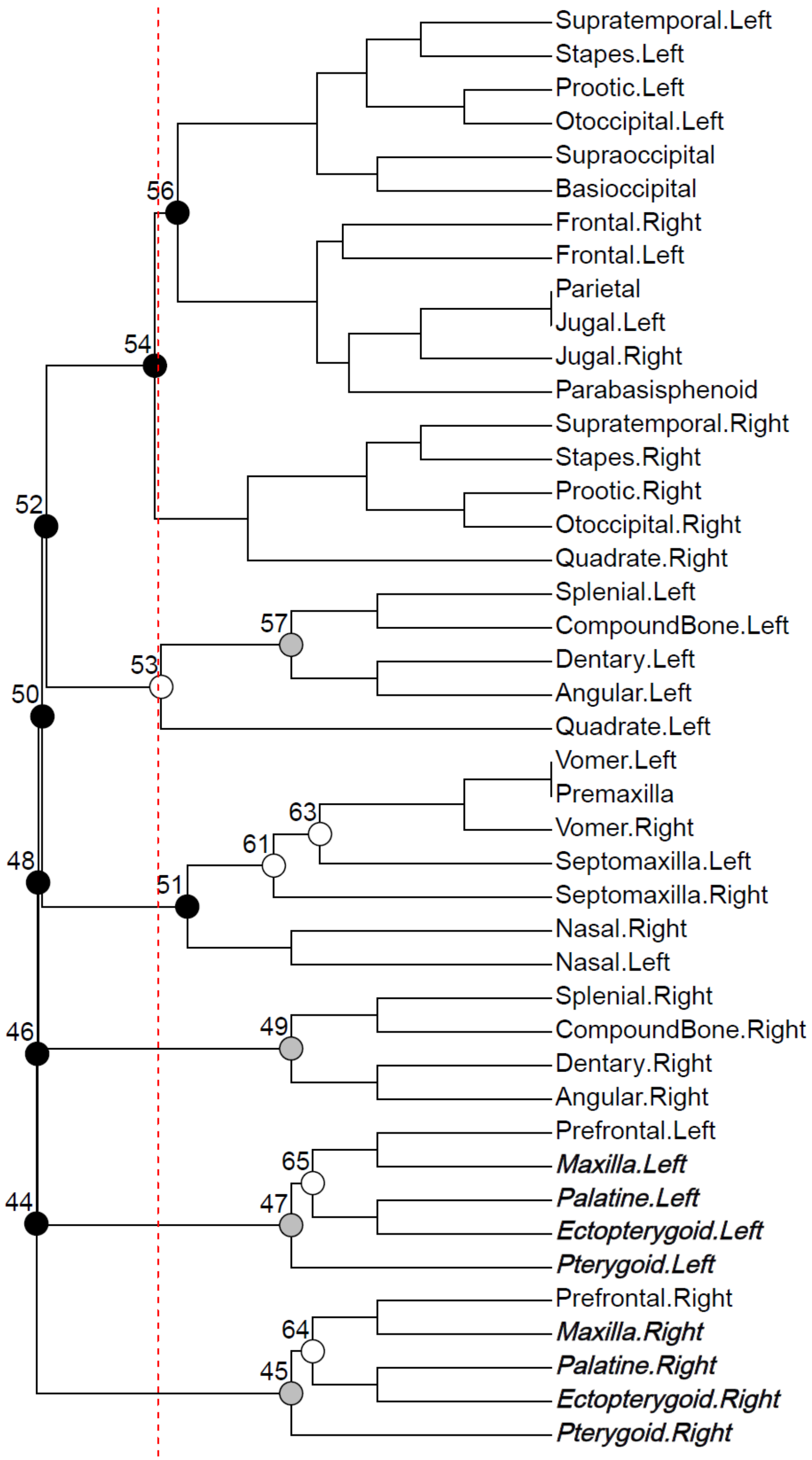

Supplementary Figure S57. Modularity of the skull network of *Thamnophis radix* (UAMZ R636).

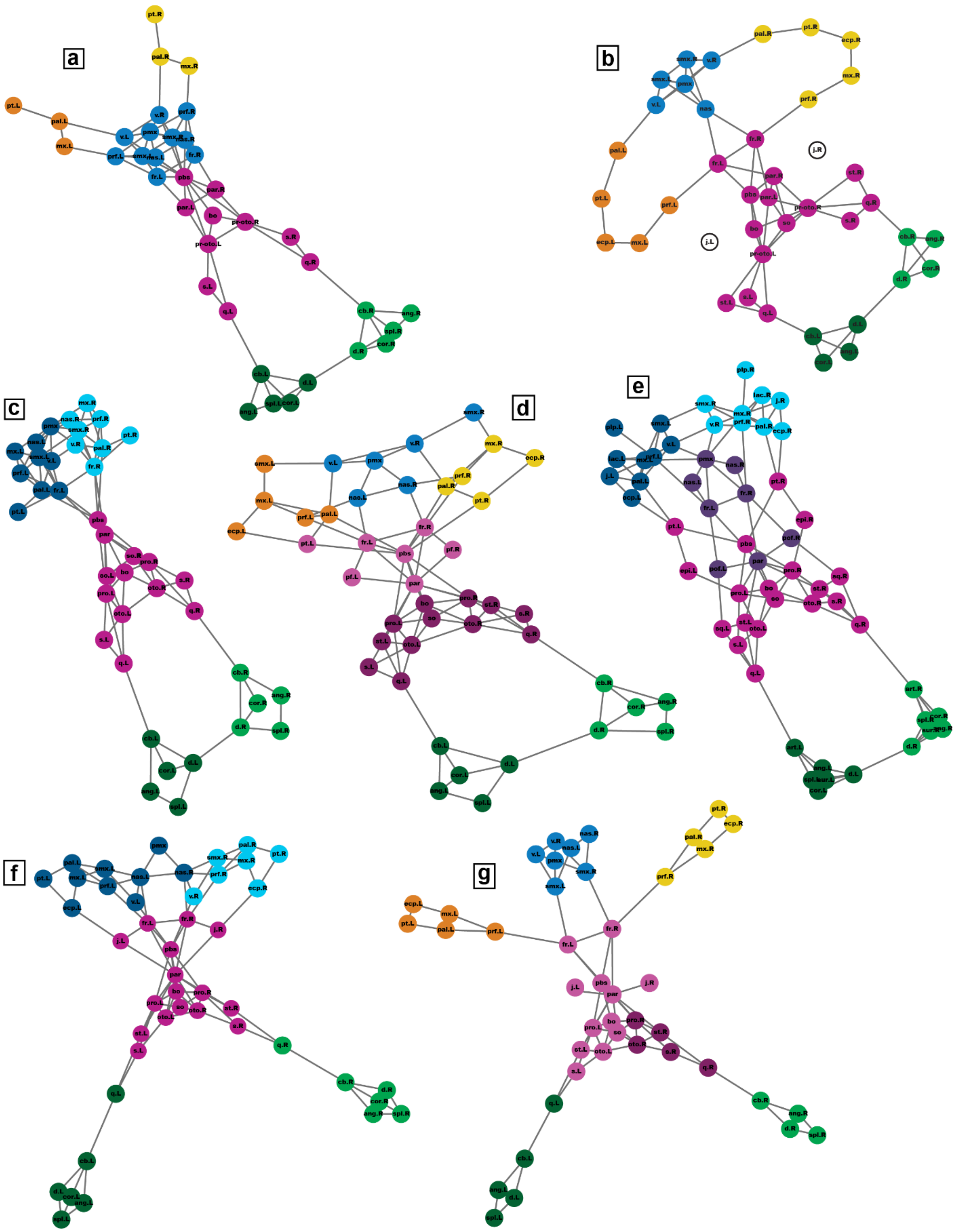
a
b
c
d
e
f
g

Supplementary Figure S58. Anatomical networks of representative squamates. Enlarged versions of the network diagrams shown in Figures 2–8, with nodes annotated. (**a**) *Xenotyphlops grandidieri* (ZSM 2194/2007), representing Typhlopoidea. (**b**) *Liotyphlops argaleus* (MCZ R-67933), representing Anomalepididae. (**c**) *Epictia albifrons* (MCZ R-2885), representing Leptotyphlopidae. (**d**) *Cylindrophis ruffus* (UMMZ 201901), representing 'Anilioidea'. (**e**) *Varanus exanthematicus* (FMNH 58299), representing non-snake lizards. (**f**) *Boa constrictor* (FMNH 31182), representing Booidea-Pythonoidea. (**g**) *Thamnophis radix* (UAMZ R636), representing Caenophidia. Abbreviations: ang, angular; art, articular; bo, basioccipital; cb, compound bone; cor, coronoid; d, dentary; ecp, ectopterygoid; epi, epipterygoid; fr, frontal; j, jugal; lac, lacrimal; mx, maxilla; nas, nasal; oto, otoccipital; pal, palatine; plp, palpebral; pbs, parabasisphenoid; par, parietal; pf, postfrontal; pof, postorbitofrontal; prf, prefrontal; pmx, premaxilla; pro, prootic; pr-oto, prootic-otoccipital; pt, pterygoid; q, quadrate; smx, septomaxilla; spl, splenial; sq, squamosal; s, stapes; so, supraoccipital; st, supratemporal; sur, surangular; v, vomer. Suffixes indicate left and right counterparts.

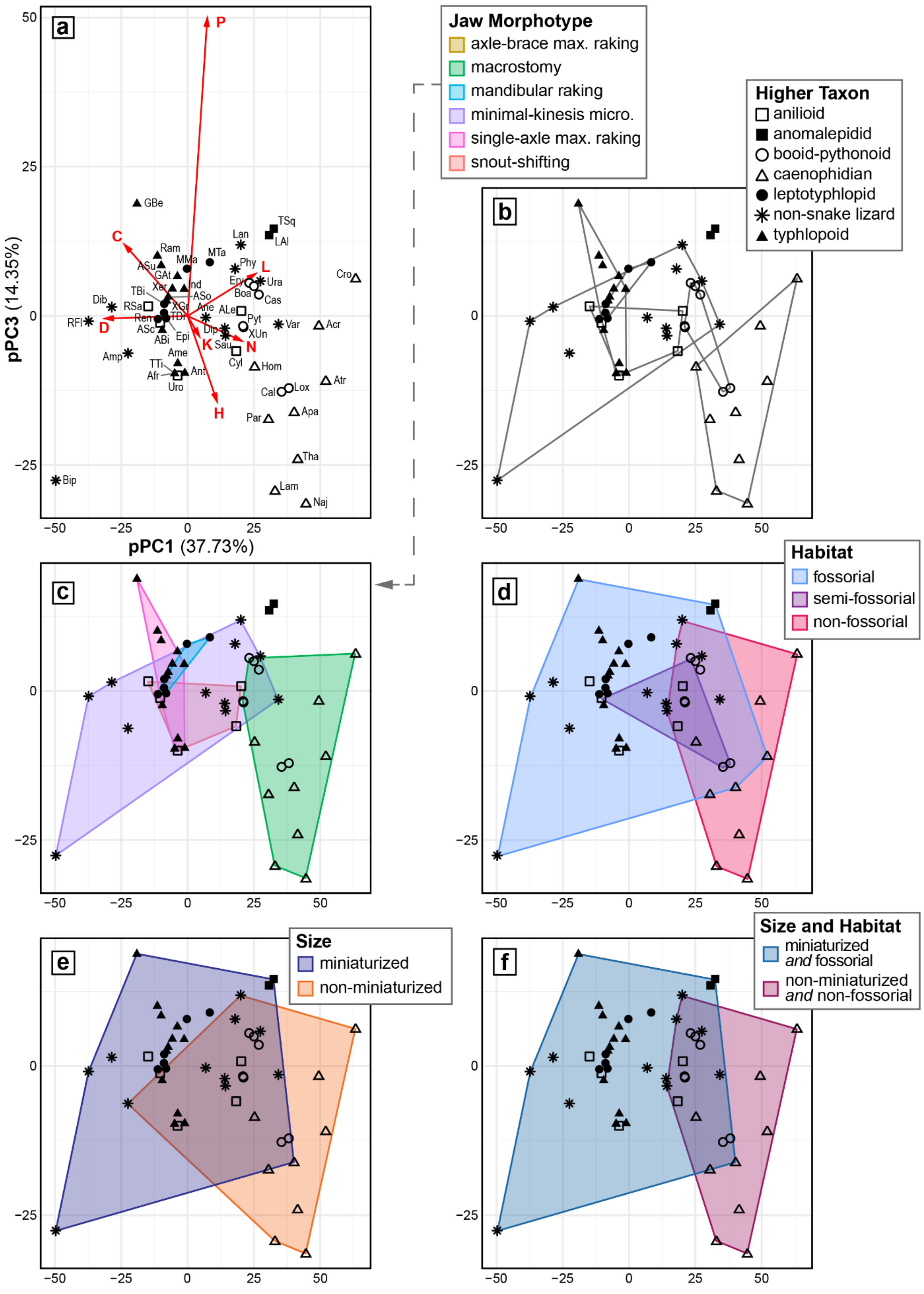
a
b
c
d
e
f
pPC1 (37.73%)
pPC3 (14.35%)
P
C
L
D
K
N
H
Jaw Morphotype
axle-brace max. raking
macrostomy
mandibular raking
minimal-kinesis micro.
single-axle max. raking
snout-shifting
Higher Taxon
anilioid
anomalepidid
booid-pythonoid
caenophidian
leptotyphlopid
non-snake lizard
typhlopoid
Habitat
fossorial
semi-fossorial
non-fossorial
Size
miniaturized
non-miniaturized
Size and Habitat
miniaturized and fossorial
non-miniaturized and non-fossorial

Supplementary Figure S59. Phylogenetic principal component analysis based on anatomical network parameters, depicting pPC1 and pPC3. Patterns of topospace occupation are represented using convex hulls. See legend in (**b**) for symbols used throughout all panels. (**a**) Biplot showing overall topospace composition. Red arrows indicate the contribution of each network parameter to these phylogenetic principal components. Taxa are labelled using the first three letters of their respective genus, or the first letter of the genus and first two letters of the specific epithet (see Supplementary Table S1). (**b**) Distribution of higher taxa across topospace. (**c**) Distribution across topospace of jaw morphotypes proposed by Strong *et al.*[4]. (**d**) Distribution of habitat types across topospace. (**e**) Distribution of size classes across topospace. (**f**) Distribution of taxa when considering size and habitat simultaneously. Abbreviations: N, number of nodes; K, number of connections; D, density of connections; C, mean clustering coefficient; L, mean shortest path length; H, heterogeneity of connections; P, parcellation. Axis labels are consistent throughout all panels.

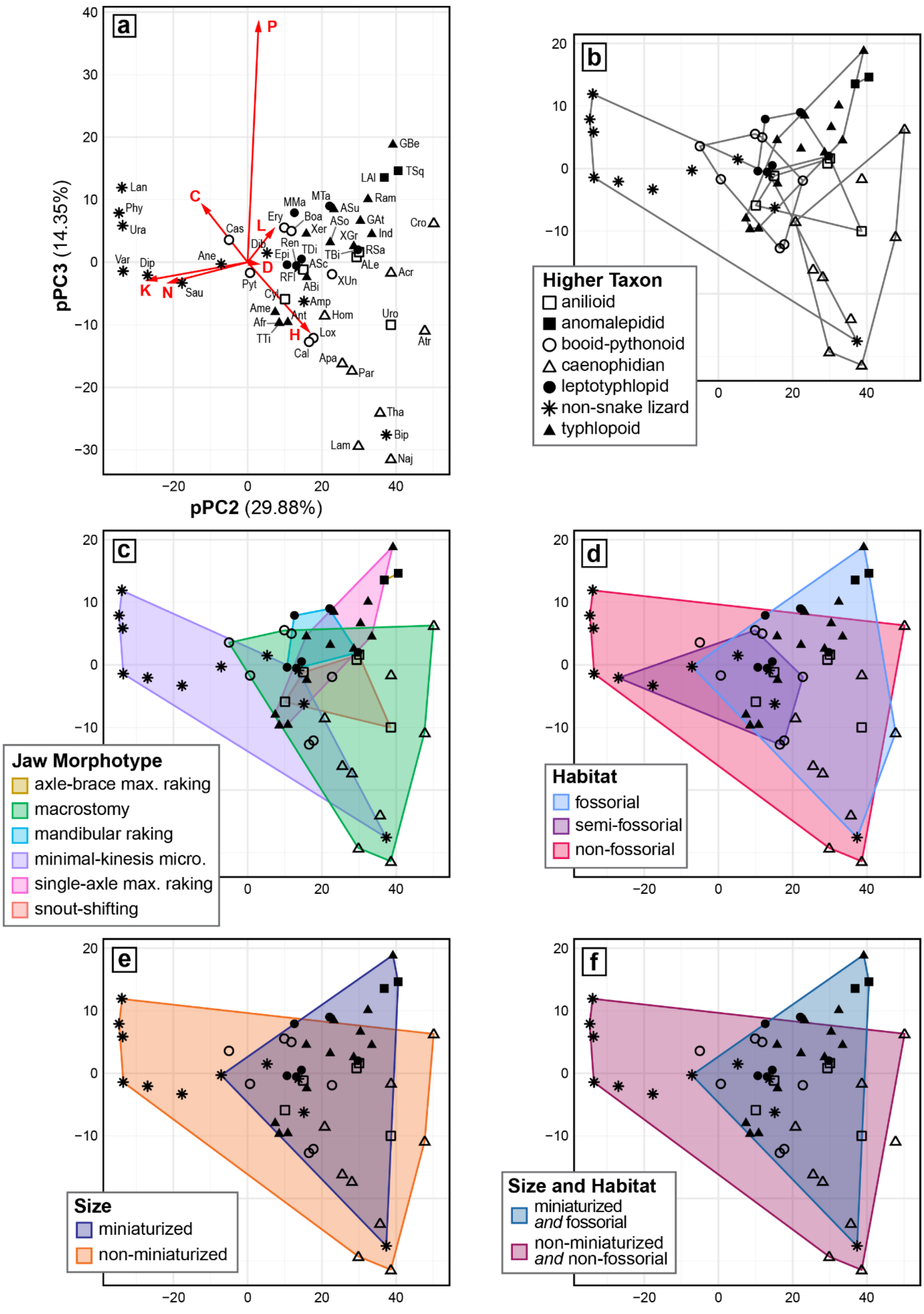
a
P
C
L
D
K
N
H
Lan
Phy
Ura
Var
Dip
Sau
Ane
Cas
Ery
MMa
MTa
Boa
ASu
ASo
Xer
Dib
Epi
Ren
TDi
TBi
XGr
GAt
Ind
RSa
ALe
ASc
RFl
ABi
Pyt
XUn
Cyl
Amp
Ame
Ant
Afr
TTi
Hom
Lox
Cal
Uro
Atr
Acr
Cro
LAl
TSq
GBe
Ram
Apa
Par
Tha
Bip
Lam
Naj
pPC3 (14.35%)
pPC2 (29.88%)
b
Higher Taxon
anilioid
anomalepidid
booid-pythonoid
caenophidian
leptotyphlopid
non-snake lizard
typhlopoid
c
Jaw Morphotype
axle-brace max. raking
macrostomy
mandibular raking
minimal-kinesis micro.
single-axle max. raking
snout-shifting
d
Habitat
fossorial
semi-fossorial
non-fossorial
e
Size
miniaturized
non-miniaturized
f
Size and Habitat
miniaturized and fossorial
non-miniaturized and non-fossorial

Supplementary Figure S60. Phylogenetic principal component analysis based on anatomical network parameters, depicting pPC2 and pPC3. Patterns of topospace occupation are represented using convex hulls. See legend in (**b**) for symbols used throughout all panels. (**a**) Biplot showing overall topospace composition. Red arrows indicate the contribution of each network parameter to these phylogenetic principal components. Taxa are labelled using the first three letters of their respective genus, or the first letter of the genus and first two letters of the specific epithet (see Supplementary Table S1). (**b**) Distribution of higher taxa across topospace. (**c**) Distribution across topospace of jaw morphotypes proposed by Strong *et al.*[4]. (**d**) Distribution of habitat types across topospace. (**e**) Distribution of size classes across topospace. (**f**) Distribution of taxa when considering size and habitat simultaneously. Abbreviations: N, number of nodes; K, number of connections; D, density of connections; C, mean clustering coefficient; L, mean shortest path length; H, heterogeneity of connections; P, parcellation. Axis labels are consistent throughout all panels.

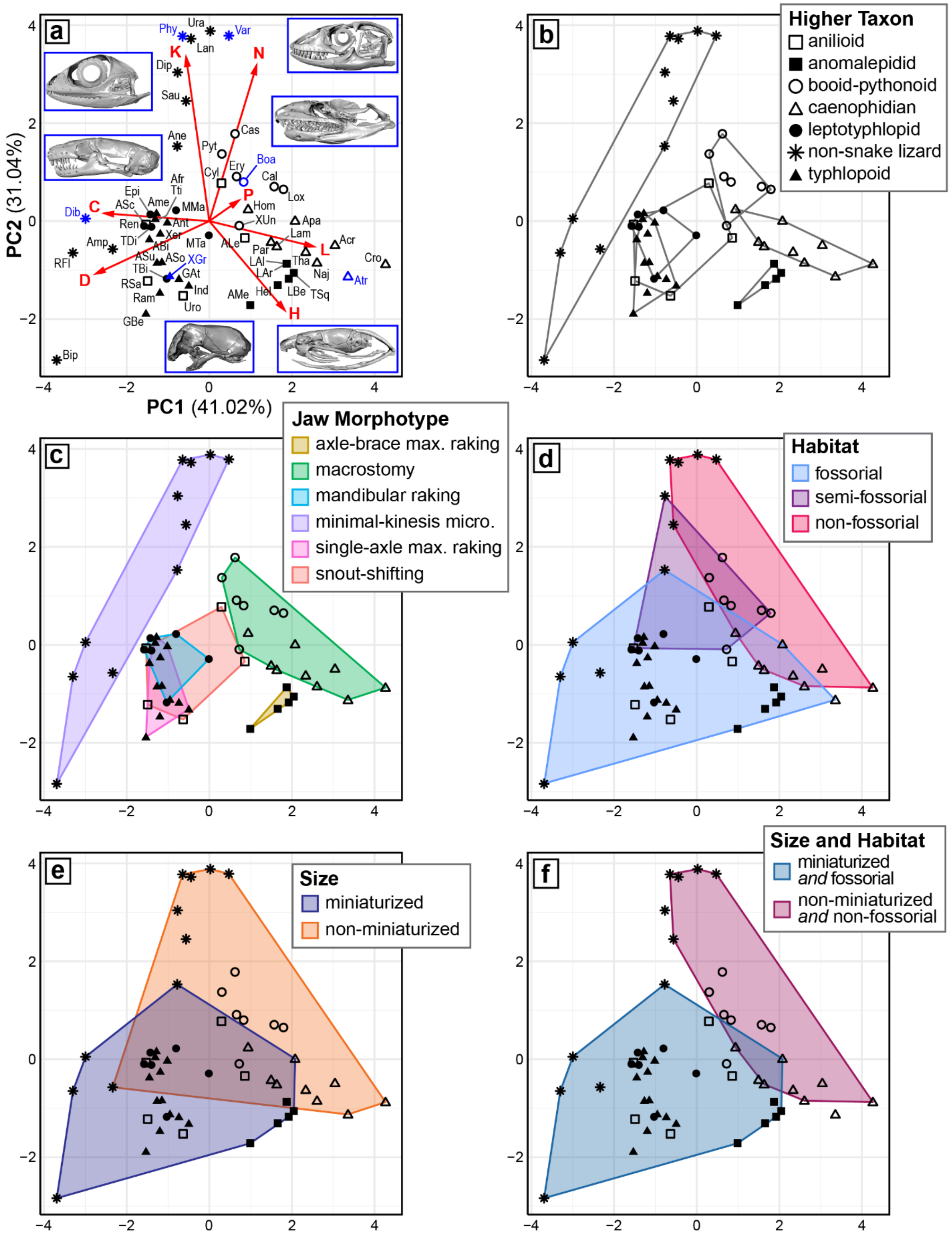
a
b
c
d
e
f
PC1 (41.02%)
PC2 (31.04%)
Higher Taxon
anilioid
anomalepidid
booid-pythonoid
caenophidian
leptotyphlopid
non-snake lizard
typhlopoid
Jaw Morphotype
axle-brace max. raking
macrostomy
mandibular raking
minimal-kinesis micro.
single-axle max. raking
snout-shifting
Habitat
fossorial
semi-fossorial
non-fossorial
Size
miniaturized
non-miniaturized
Size and Habitat
miniaturized and fossorial
non-miniaturized and non-fossorial

Supplementary Figure S61. Principal component analysis based on anatomical network parameters, depicting PC1 and PC2. Patterns of topospace occupation are represented using convex hulls. See legend in (**b**) for symbols used throughout all panels. (**a**) Biplot showing overall topospace composition. Red arrows indicate the contribution of each network parameter to the first two principal components. Specimens are labelled using the first three letters of their respective genus, or the first letter of the genus and first two letters of the specific epithet (see Supplementary Table S1). Representative specimens are indicated in blue. (**b**) Distribution of higher taxa across topospace. Non-snake lizards, anomalepidids, booid-pythonoids, and caenophidians occupy generally distinct regions, whereas typhlopoids, leptotyphlopids, and anilioids overlap extensively. (**c**) Distribution across topospace of jaw morphotypes proposed by Strong *et al.*[4]. (**d**) Distribution of habitat types across topospace. Fossorial and non-fossorial taxa occupy distinct regions, although these regions do overlap somewhat. Semi-fossorial taxa occupy an intermediate region overlapping broadly with both other habitats. (**e**) Distribution of size classes across topospace. Miniaturized and non-miniaturized taxa both occupy large regions of topospace; these regions are generally distinct but do exhibit noticeable overlap. (**f**) Distribution of taxa when considering size and habitat simultaneously. Miniaturization and fossoriality together define a more distinct region of topospace than when either phenomenon is considered individually, reflected by reduced overlap between opposing regions (i.e., miniaturized–fossorial *versus* non-miniaturized–non-fossorial). Abbreviations: N, number of nodes; K, number of connections; D, density of connections; C, mean clustering coefficient; L, mean shortest path length; H, heterogeneity of connections; P, parcellation. Axis labels are consistent throughout all panels.

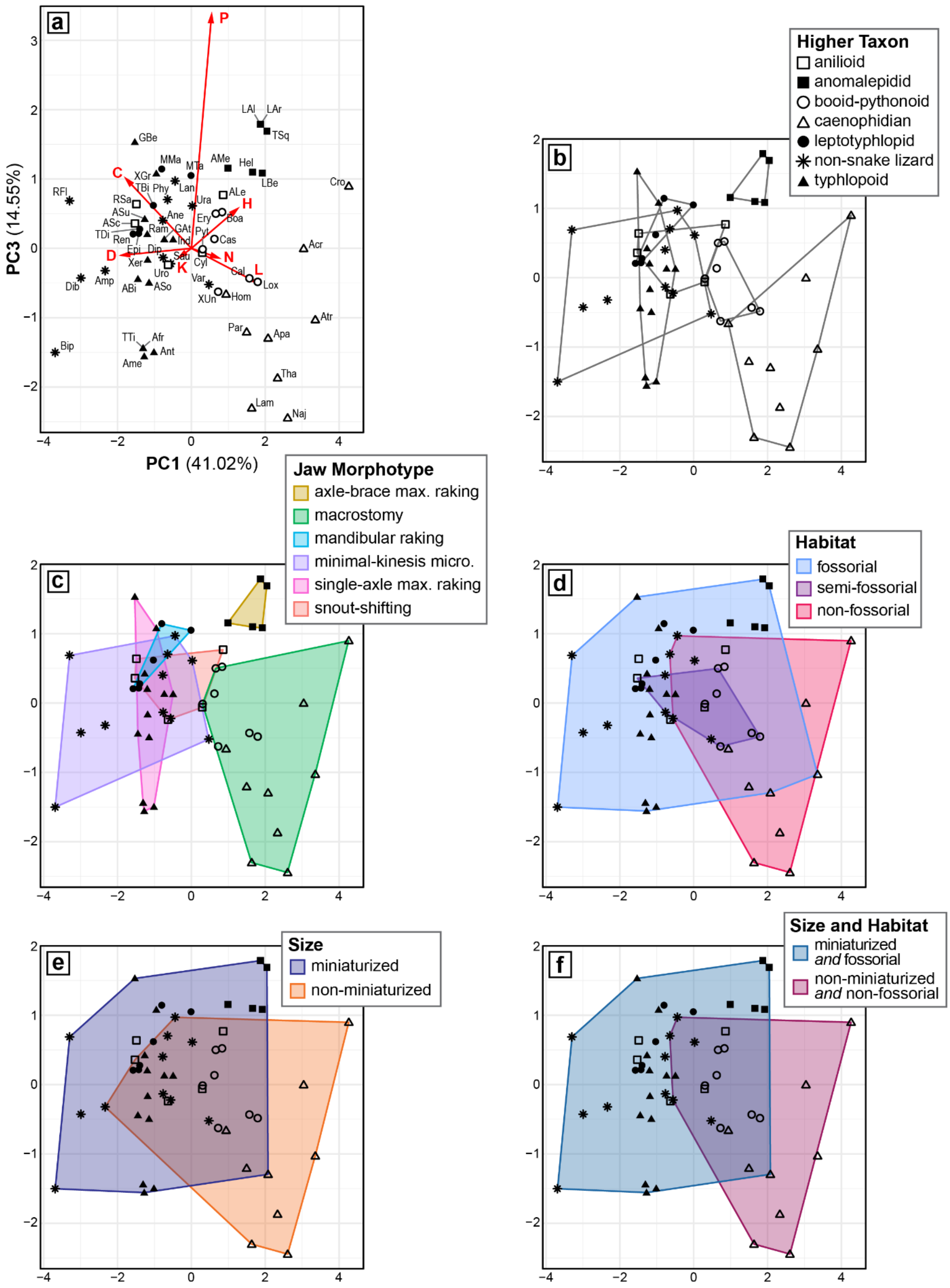
a
PC3 (14.55%)
PC1 (41.02%)
P
C
H
D
K
N
L
b
Higher Taxon
anilioid
anomalepidid
booid-pythonoid
caenophidian
leptotyphlopid
non-snake lizard
typhlopoid
c
Jaw Morphotype
axle-brace max. raking
macrostomy
mandibular raking
minimal-kinesis micro.
single-axle max. raking
snout-shifting
d
Habitat
fossorial
semi-fossorial
non-fossorial
e
Size
miniaturized
non-miniaturized
f
Size and Habitat
miniaturized
and fossorial
non-miniaturized
and non-fossorial

Supplementary Figure S62. Principal component analysis based on anatomical network parameters, depicting PC1 and PC3. Patterns of topospace occupation are represented using convex hulls. See legend in (**b**) for symbols used throughout all panels. (**a**) Biplot showing overall topospace composition. Red arrows indicate the contribution of each network parameter to these principal components. Taxa are labelled using the first three letters of their respective genus, or the first letter of the genus and first two letters of the specific epithet (see Supplementary Table S1). (**b**) Distribution of higher taxa across topospace. (**c**) Distribution across topospace of jaw morphotypes proposed by Strong *et al.*[4]. (**d**) Distribution of habitat types across topospace. (**e**) Distribution of size classes across topospace. (**f**) Distribution of taxa when considering size and habitat simultaneously. Abbreviations: N, number of nodes; K, number of connections; D, density of connections; C, mean clustering coefficient; L, mean shortest path length; H, heterogeneity of connections; P, parcellation. Axis labels are consistent throughout all panels.

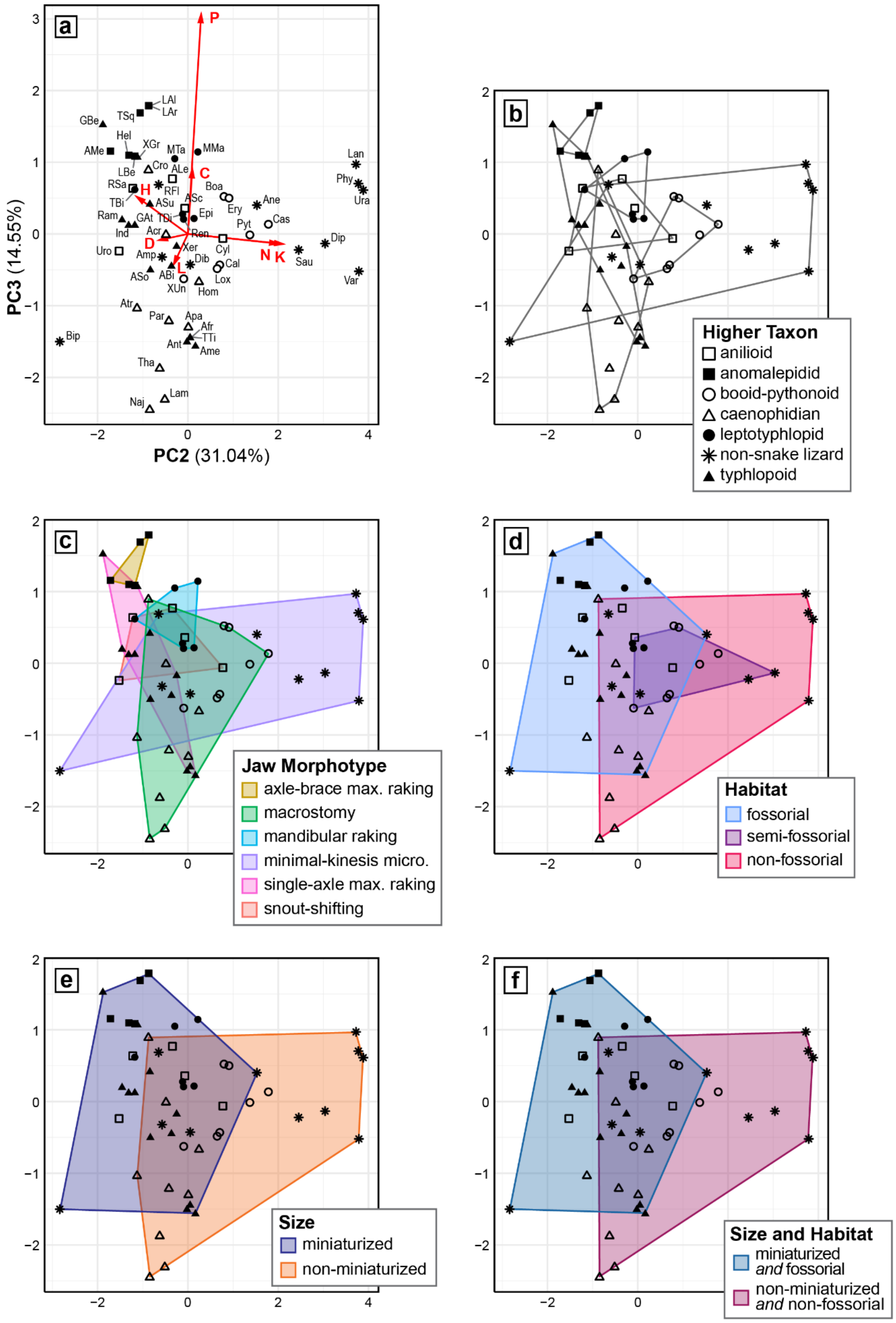
a
PC2 (31.04%)
PC3 (14.55%)
P
C
H
D
L
N K
b
Higher Taxon
anilioid
anomalepidid
booid-pythonoid
caenophidian
leptotyphlopid
non-snake lizard
typhlopoid
c
Jaw Morphotype
axle-brace max. raking
macrostomy
mandibular raking
minimal-kinesis micro.
single-axle max. raking
snout-shifting
d
Habitat
fossorial
semi-fossorial
non-fossorial
e
Size
miniaturized
non-miniaturized
f
Size and Habitat
miniaturized *and* fossorial
non-miniaturized *and* non-fossorial

Supplementary Figure S63. Principal component analysis based on anatomical network parameters, depicting PC2 and PC3. Patterns of topospace occupation are represented using convex hulls. See legend in (**b**) for symbols used throughout all panels. (**a**) Biplot showing overall topospace composition. Red arrows indicate the contribution of each network parameter to these principal components. Taxa are labelled using the first three letters of their respective genus, or the first letter of the genus and first two letters of the specific epithet (see Supplementary Table S1). (**b**) Distribution of higher taxa across topospace. (**c**) Distribution across topospace of jaw morphotypes proposed by Strong *et al.*[4]. (**d**) Distribution of habitat types across topospace. (**e**) Distribution of size classes across topospace. (**f**) Distribution of taxa when considering size and habitat simultaneously. Abbreviations: N, number of nodes; K, number of connections; D, density of connections; C, mean clustering coefficient; L, mean shortest path length; H, heterogeneity of connections; P, parcellation. Axis labels are consistent throughout all panels.

Supplementary Tables

Supplementary Table S1. List of specimens analyzed in this study

Taxonomic assignments are based on Burbrink *et al.*[5]. See Methods and Supplementary Methods for further information regarding sources of micro-CT scan data. See Figure 1 for phylogenetic context. Digital access information is provided via a hyperlink to the repository URL for each specimen, with the associated Archival Resource Key (ARK) identifier indicated for data obtained from MorphoSource. For these latter specimens, specimen numbers are provided in 'Darwin Core Triplet'-format (i.e., [institution code]:[collection code]:[catalog number]). Institutional abbreviations: AMS, Australian Museum, Sydney, Australia; CAS, California Academy of Sciences, San Francisco, USA; FMNH, Field Museum of Natural History, Chicago, USA; FRIM, Forest Research Institute Malaysia, Kuala Lumpur, Malaysia; KUH, University of Kansas Biodiversity Institute and Natural History Museum, Lawrence, USA; MCZ, Museum of Comparative Zoology, Harvard University, Cambridge, USA; SAMA, South Australian Museum, Adelaide, Australia; TCWC, Biodiversity Research and Teaching Collections, Texas A&M University, College Station, USA; TNHC, Texas Natural History Collections, Texas Memorial Museum of Science and History, University of Texas at Austin, Austin, USA; UAMZ, University of Alberta Museum of Zoology, Edmonton, Canada; UF, Florida Museum of Natural History, University of Florida, Gainesville, USA; UMMZ, University of Michigan Museum of Zoology, Ann Arbor, USA; YPM, Yale Peabody Museum, New Haven, USA; ZSM, Zoologische Staatssammlung München, Munich, Germany.

<table>
<tr><th colspan="3">HIGHER TAXON</th><th>SPECIES</th><th>SPECIMEN NUMBER</th><th>DIGITAL ACCESS</th></tr>
<tr><td rowspan="6">Alethinophidia</td><td rowspan="6">'Anilioidea'</td><td>Amerophidia (Aniliidae)</td><td>Anilius scytale</td><td>KU:KUH:125976</td><td>ark:/87602/m4/M75015</td></tr>
<tr><td rowspan="5">Uropeltoidea (Anomochilidae) (Cylindrophiidae) (Uropeltidae x2)</td><td>Anomochilus leonardi</td><td>FRIM 0026</td><td>DigiMorph</td></tr>
<tr><td rowspan="2">Cylindrophis ruffus</td><td>UMMZ:Herps: 201901</td><td>ark:/87602/m4/M70987</td></tr>
<tr><td>UF:Herp:143722</td><td>ark:/87602/m4/445119</td></tr>
<tr><td>Rhinophis sanguineus</td><td>UF:Herp:78397</td><td>ark:/87602/m4/M72220</td></tr>
<tr><td>Uropeltis melanogaster</td><td>FMNH 167048</td><td>DigiMorph</td></tr>
</table>

	**Bolyeriidae**		*Casarea dussumieri*	UMMZ 190285	DigiMorph
	**Booidea**	**Boidae**	*Boa constrictor*	FMNH:amphibians and reptiles:31182	ark:/87602/m4/M98393
		**Calabariidae**	*Calabaria reinhardtii*	FMNH 117833	DigiMorph
		**Erycidae**	*Eryx colubrinus*	FMNH 63117	DigiMorph
	**Caenophidia**	**Acrochordidae**	*Acrochordus granulatus*	MCZ R-146128	Upload pending
		**Atractaspididae**	*Aparallactus guentheri*	MCZ R-23363	Upload pending
			Atractaspis irregularis	FMNH 62204	DigiMorph
				UF:Herp:68168	ark:/87602/m4/M72081
		**Colubridae**	*Lampropeltis getula*	FMNH 95184	DigiMorph
		**Elapidae**	*Naja naja*	FMNH 22468	DigiMorph
		**Homalopsidae**	*Homalopsis buccata*	FMNH:amphibians and reptiles:259340	ark:/87602/m4/M97478
		**Natricidae**	*Thamnophis radix*	UAMZ R636	Available on request
		**Pareidae**	*Pareas hamptoni*	FMNH:amphibians and reptiles:128304	ark:/87602/m4/M98586
		**Viperidae**	*Crotalus adamanteus*	UF:Herp:103268	ark:/87602/m4/M48786
	**Pythonoidea**	**Loxocemidae**	*Loxocemus bicolor*	FMNH 104800	DigiMorph
		**Pythonidae**	*Python molurus*	TNHC 62769	DigiMorph
		**Xenopeltidae**	*Xenopeltis unicolor*	FMNH 148900	DigiMorph
**'Scolecophidia'**	**Anomalepididae**		*Anomalepis mexicanus*	MCZ R-191201	Upload pending
			Helminthophis praeocularis	MCZ R-17960	Upload pending
			Liotyphlops albirostris	FMNH 216257	DigiMorph
				KU:KUH:116885	ark:/87602/m4/M75008
			Liotyphlops argaleus	MCZ R-67933	Upload pending
			Liotyphlops beui	SAMA 40142	Available on request
			Typhlophis squamosus	MCZ R-145403	Upload pending
	**Leptotyphlopidae**		*Epictia albifrons*	MCZ R-2885	Upload pending
			Myriopholis macrorhyncha	MCZ R-9650	Upload pending
			Myriopholis tanae	MCZ R-40099	Upload pending

			Rena dulcis	UAMZ R335	Available on request
			Tricheilostoma bicolor	MCZ R-49718	Upload pending
			Trilepida dimidiata	SAMA 40143	Available on request
	**Typhlopoidea**	**Gerrhopilidae**	*Gerrhopilus ater*	MCZ R-33505	Upload pending
			Gerrhopilus beddomii	MCZ R-22372	Upload pending
		**Typhlopidae**	*Acutotyphlops solomonis*	AMS R11452	Available on request
			Acutotyphlops subocularis	SAMA R64770	Available on request
			Afrotyphlops angolensis	MCZ R-170385	Upload pending
			Amerotyphlops paucisquamus	MCZ R-147336	Upload pending
			Anilios bicolor	SAMA 60626	Available on request
			Antillotyphlops monastus	MCZ R-81112	Upload pending
			Indotyphlops braminus	UAMZ R363	Available on request
			Ramphotyphlops lineatus	MCZ R-37751	Upload pending
			Typhlops titanops	MCZ R-68571	Upload pending
			Xerotyphlops vermicularis	MCZ R-56477	Upload pending
		**Xenotyphlopidae**	*Xenotyphlops grandidieri*	ZSM:2194/2007	ark:/87602/m4/M79510
**'Non-snake lizards'**	**Amphisbaenia**	**Amphisbaenidae**	*Amphisbaena fuliginosa*	FMNH:amphibians and reptiles:22847	ark:/87602/m4/M98383
		**Bipedidae**	*Bipes biporus*	CAS:Herp:126478	ark:/87602/m4/M98391
		**Rhineuridae**	*Rhineura floridana*	FMNH:amphibians and reptiles:31774	ark:/87602/m4/M98600
	**Dibamidae**		*Anelytropsis papillosus*	TCWC:Herpetology:45501	ark:/87602/m4/M113753
			Dibamus novaeguineae	UF:Herp:33488	ark:/87602/m4/M62342
	**Iguania**	**Agamidae**	*Physignathus cocincinus*	YPM 14378	DigiMorph
		**Iguanidae**	*Dipsosaurus dorsalis*	YPM 14376	DigiMorph
			Sauromalus ater	TNHC 18483	DigiMorph
		**Tropiduridae**	*Uranoscodon superciliosus*	YPM 12871	DigiMorph

	**Varanoidea**	**Lanthanotidae**	*Lanthanotus borneensis*	FMNH 148589	DigiMorph
		**Varanidae**	*Varanus exanthematicus*	FMNH 58299	DigiMorph

Supplementary Table S2. Measurements of skull length (mm) in observed taxa

These measurements were used to delimit 'miniaturization' *versus* 'non-miniaturization', with the break in distribution between *Rhineura* (11.74 mm) and *Acrochordus* (14.05 mm) being chosen as the most reasonable cut-off (see Methods; Fig. 9e,f; Supplementary Figs S59–S63; Supplementary Data File 4).

TAXON	SKULL LENGTH (MM)
Myriopholis tanae	2.56
Liotyphlops beui	3.17
Trilepida	3.18
Tricheilostoma	3.62
Gerrhopilus ater	3.82
Myriopholis macrorhyncha	4.02
Liotyphlops albirostris	4.10
Indotyphlops	4.11
Helminthophis	4.17
Gerrhopilus beddomii	4.29
Typhlophis	4.36
Rena	4.39
Epictia	4.67
Liotyphlops argaleus	4.76
Xenotyphlops	5.08
Xerotyphlops	5.14
Anomalepis	5.14
Antillotyphlops	5.23
Acutotyphlops solomonis	6.33
Acutotyphlops subocularis	6.56
Amerotyphlops	6.61
Typhlops	6.63
Anomochilus	6.85
Anilios	7.12
Bipes	7.12
Ramphotyphlops	7.16
Dibamus	8.20
Anelytropsis	8.60
Rhinophis	9.57
Uropeltis	10.48
Afrotyphlops	10.59

Aparallactus	11.54
Pareas	11.60
Rhineura	11.74
Acrochordus	14.05
Atractaspis	14.74
Casarea	16.30
Amphisbaena	17.46
Eryx	18.93
Calabaria	22.91
Cylindrophis	23.3
Dipsosaurus	23.87
Lanthanotus	24.38
Anilius	25.00
Thamnophis	25.50
Crotalus	27.48
Loxocemus	27.64
Uranoscodon	28.04
Physignathus	29.48
Xenopeltis	29.93
Lampropeltis	30.38
Homalopsis	30.70
Naja	31.64
Sauromalus	34.90
Varanus	57.41
Python	68.03
Boa	71.80

Supplementary Table S3. PERMANOVA statistical results for pPCA

As part of a phylogenetically corrected PCA based on the calculated network parameters (see Methods; Table 1; Supplementary Data File 3), we grouped specimens according to several variables, using patterns of topospace occupation to assess phenomena such as convergence (Fig. 9; Supplementary Figs S59 and S60; Supplementary Data Files 4 and 5). We then assessed the statistical significance of each of these grouping methods via PERMANOVA, as reported here. See main text for details. Asterisks mark significance at $\alpha = 0.05$ (*), 0.01 (**), and 0.001 (***). Abbreviations: df, degrees of freedom.

VARIABLE	COMPARISON	F-STATISTIC	$DF_{TREATMENT, RESIDUALS}$	P-VALUE
**Higher Taxon**	Typhlopoidea *vs* Anomalepididae	23.664	1,13	0.0102 *
	Typhlopoidea *vs* Leptotyphlopidae	0.680	1,17	0.4855
	Typhlopoidea *vs* Caenophidia	77.071	1,20	0.0001 ***
	Typhlopoidea *vs* Booidea-Pythonoidea	41.063	1,18	0.0001 ***
	Typhlopoidea *vs* Anilioidea	1.570	1,16	0.2061
	Typhlopoidea *vs* Non-snake lizards	8.495	1,22	0.0052 **
	Anomalepididae *vs* Leptotyphlopidae	29.005	1,6	0.0354 *
	Anomalepididae *vs* Caenophidia	0.973	1,9	0.3522
	Anomalepididae *vs* Booidea-Pythonoidea	10.068	1,7	0.0283 *
	Anomalepididae *vs* Anilioidea	4.735	1,5	0.0952

	Anomalepididae *vs* Non-snake lizards	4.387	1,11	0.0530
	Leptotyphlopidae *vs* Caenophidia	48.374	1,13	0.0001 ***
	Leptotyphlopidae *vs* Booidea-Pythonoidea	26.952	1,11	0.0008 ***
	Leptotyphlopidae *vs* Anilioidea	1.150	1,9	0.3300
	Leptotyphlopidae *vs* Non-snake lizards	3.019	1,15	0.0958
	Caenophidia *vs* Booidea-Pythonoidea	16.309	1,14	0.0010 ***
	Caenophidia *vs* Anilioidea	18.971	1,12	0.0007 ***
	Caenophidia *vs* Non-snake lizards	20.969	1,18	0.0001 ***
	Booidea-Pythonoidea *vs* Anilioidea	9.835	1,10	0.0027 **
	Booidea-Pythonoidea *vs* Non-snake lizards	5.343	1,16	0.0137 *
	Anilioidea *vs* Non-snake lizards	3.682	1,14	0.0558
**Jaw Mechanism**	Single-axle max. raking *vs* Axle-brace max. raking	23.664	1,13	0.0097 **
	Single-axle max. raking *vs* Mandibular raking	0.680	1,17	0.4713
	Single-axle max. raking *vs* Macrostomy	46.052	1,27	0.0001 ***
	Single-axle max. raking *vs* Snout-shifting	1.570	1,16	0.2155
	Single-axle max. raking	8.495	1,22	0.0051 **

	vs Minimal-kinesis micro.			
	Axle-brace max. raking *vs* Mandibular raking	29.005	1,6	0.0382 *
	Axle-brace max. raking *vs* Macrostomy	1.055	1,16	0.3265
	Axle-brace max. raking *vs* Snout-shifting	4.735	1,5	0.0952
	Axle-brace max. raking *vs* Minimal-kinesis micro.	4.387	1,11	0.0487 *
	Mandibular raking *vs* Macrostomy	22.583	1,20	0.0002 ***
	Mandibular raking *vs* Snout-shifting	1.150	1,9	0.3234
	Mandibular raking *vs* Minimal-kinesis micro.	3.019	1,15	0.0897
	Macrostomy *vs* Snout-shifting	10.841	1,19	0.0003 ***
	Macrostomy *vs* Minimal-kinesis micro.	19.873	1,25	0.0001 ***
	Snout-shifting *vs* Minimal-kinesis micro.	3.682	1,14	0.0561
**Habitat**	Fossorial *vs* Non-fossorial	19.028	1,44	0.0001 ***
	Fossorial *vs* Semi-fossorial	6.723	1,36	0.0039 **
	Non-fossorial *vs* Semi-fossorial	0.723	1,20	0.4457
**Size**	Miniaturized *vs* Non-miniaturized	18.038	1,51	0.0001 ***
**Combined Habitat–Size**	Fossorial–Miniaturized *vs*	20.822	1,41	0.0001 ***

	Non-fossorial–Non-mini.			
	Fossorial–Miniaturized *vs* Fossorial or Miniaturized	7.960	1,37	0.0023 **
	Non-fossorial–Non-mini. *vs* Fossorial or Miniaturized	1.416	1,22	0.2414

Supplementary Table S4. PERMANOVA statistical results for PCA

As part of a PCA based on the calculated network parameters (see Methods; Table 1; Supplementary Data File 3), we grouped specimens according to several variables, using patterns of topospace occupation to assess phenomena such as convergence (Supplementary Figs S61–S63; Supplementary Data Files 4 and 6). We then assessed the statistical significance of each of these grouping methods via PERMANOVA, as reported here. See main text for details. Asterisks mark significance at $\alpha = 0.05$ (*), 0.01 (**), and 0.001 (***). Abbreviations: df, degrees of freedom.

VARIABLE	COMPARISON	F-STATISTIC	$DF_{TREATMENT, RESIDUALS}$	P-VALUE
**Higher Taxon**	Typhlopoidea *vs* Anomalepididae	77.141	1,17	0.0001 ***
	Typhlopoidea *vs* Leptotyphlopidae	1.731	1,17	0.1878
	Typhlopoidea *vs* Caenophidia	82.453	1,20	0.0001 ***
	Typhlopoidea *vs* Booidea-Pythonoidea	53.823	1,18	0.0001 ***
	Typhlopoidea *vs* Anilioidea	1.856	1,16	0.1576
	Typhlopoidea *vs* Non-snake lizards	8.970	1,22	0.0051 **
	Anomalepididae *vs* Leptotyphlopidae	61.192	1,10	0.0018 **
	Anomalepididae *vs* Caenophidia	3.749	1,13	0.0434 *
	Anomalepididae *vs* Booidea-Pythonoidea	33.381	1,11	0.0012 **
	Anomalepididae *vs* Anilioidea	14.514	1,9	0.0021 **

	Anomalepididae *vs* Non-snake lizards	13.434	1,15	0.0003 ***
	Leptotyphlopidae *vs* Caenophidia	43.611	1,13	0.0001 ***
	Leptotyphlopidae *vs* Booidea-Pythonoidea	26.533	1,11	0.0005 ***
	Leptotyphlopidae *vs* Anilioidea	0.811	1,9	0.4401
	Leptotyphlopidae *vs* Non-snake lizards	2.713	1,15	0.1088
	Caenophidia *vs* Booidea-Pythonoidea	16.494	1,14	0.0007 ***
	Caenophidia *vs* Anilioidea	18.309	1,12	0.0004 ***
	Caenophidia *vs* Non-snake lizards	20.013	1,18	0.0001 ***
	Booidea-Pythonoidea *vs* Anilioidea	9.561	1,10	0.0038 **
	Booidea-Pythonoidea *vs* Non-snake lizards	5.122	1,16	0.0169 *
	Anilioidea *vs* Non-snake lizards	3.079	1,14	0.0817
**Jaw Mechanism**	Single-axle max. raking *vs* Axle-brace max. raking	77.141	1,17	0.0001 ***
	Single-axle max. raking *vs* Mandibular raking	1.731	1,17	0.1899
	Single-axle max. raking *vs* Macrostomy	48.107	1,27	0.0001 ***
	Single-axle max. raking *vs* Snout-shifting	1.856	1,16	0.1635
	Single-axle max. raking	8.970	1,22	0.0044 **

	vs Minimal-kinesis micro.			
	Axle-brace max. raking *vs* Mandibular raking	61.192	1,10	0.0026 **
	Axle-brace max. raking *vs* Macrostomy	4.407	1,20	0.0287 *
	Axle-brace max. raking *vs* Snout-shifting	14.514	1,9	0.0021 **
	Axle-brace max. raking *vs* Minimal-kinesis micro.	13.434	1,15	0.0001 ***
	Mandibular raking *vs* Macrostomy	21.053	1,20	0.0001 ***
	Mandibular raking *vs* Snout-shifting	0.811	1,9	0.4349
	Mandibular raking *vs* Minimal-kinesis micro.	2.713	1,15	0.1145
	Macrostomy *vs* Snout-shifting	10.372	1,19	0.0009 ***
	Macrostomy *vs* Minimal-kinesis micro.	19.037	1,25	0.0001 ***
	Snout-shifting *vs* Minimal-kinesis micro.	3.079	1,14	0.0851
**Habitat**	Fossorial *vs* Non-fossorial	16.800	1,48	0.0001 ***
	Fossorial *vs* Semi-fossorial	5.880	1,40	0.0073 **
	Non-fossorial *vs* Semi-fossorial	0.680	1,20	0.4634
**Size**	Miniaturized *vs* Non-miniaturized	16.260	1,55	0.0001 ***
**Combined Habitat–Size**	Fossorial–Miniaturized *vs*	18.036	1,45	0.0001 ***

	Non-fossorial–Non-mini.			
	Fossorial–Miniaturized<br>*vs*<br>Fossorial or Miniaturized	5.789	1,41	0.0087 **
	Non-fossorial–Non-mini.<br>*vs*<br>Fossorial or Miniaturized	1.372	1,22	0.2405

References – Supplementary Information

1 Gauthier, J. A., Kearney, M., Maisano, J. A., Rieppel, O. & Behlke, A. D. B. Assembling the squamate tree of life: perspectives from the phenotype and the fossil record. *Bull. Peabody Mus. Nat. Hist.* **53**, 3–308 (2012).

2 Chretien, J., Wang-Claypool, C. Y., Glaw, F. & Scherz, M. D. The bizarre skull of *Xenotyphlops* sheds light on synapomorphies of Typhlopoidea. *J. Anat.* **234**, 637–655 (2019).

3 Esteve-Altava, B., Molnar, J. L., Johnston, P., Hutchinson, J. R. & Diogo, R. Anatomical network analysis of the musculoskeletal system reveals integration loss and parcellation boost during the fins-to-limbs transition. *Evolution* **72**, 601–618 (2018).

4 Strong, C. R. C., Scherz, M. D. & Caldwell, M. W. Deconstructing the Gestalt: new concepts and tests of homology, as exemplified by a re-conceptualization of "microstomy" in squamates. *Anat. Rec.* **304**, 2303–2351 (2021).

5 Burbrink, F. T. *et al.* Interrogating genomic-scale data for Squamata (lizards, snakes, and amphisbaenians) shows no support for key traditional morphological relationships. *Syst. Biol.* **69**, 502–520 (2020).
